# Investigating the Origins of Membrane Phospholipid Biosynthesis Genes Using Outgroup-Free Rooting

**DOI:** 10.1093/gbe/evz034

**Published:** 2019-02-08

**Authors:** Gareth A Coleman, Richard D Pancost, Tom A Williams

**Affiliations:** 1 School of Biological Sciences, University of Bristol, United Kingdom; 2 School of Earth Sciences, University of Bristol, United Kingdom

**Keywords:** lipid divide, lipid evolution, phylogenetics, outgroup-free rooting

## Abstract

One of the key differences between Bacteria and Archaea is their canonical membrane phospholipids, which are synthesized by distinct biosynthetic pathways with nonhomologous enzymes. This “lipid divide” has important implications for the early evolution of cells and the type of membrane phospholipids present in the last universal common ancestor. One of the main challenges in studies of membrane evolution is that the key biosynthetic genes are ancient and their evolutionary histories are poorly resolved. This poses major challenges for traditional rooting methods because the only available outgroups are distantly related. Here, we address this issue by using the best available substitution models for single-gene trees, by expanding our analyses to the diversity of uncultivated prokaryotes recently revealed by environmental genomics, and by using two complementary approaches to rooting that do not depend on outgroups. Consistent with some previous analyses, our rooted gene trees support extensive interdomain horizontal transfer of membrane phospholipid biosynthetic genes, primarily from Archaea to Bacteria. They also suggest that the capacity to make archaeal-type membrane phospholipids was already present in last universal common ancestor.

## Introduction

**Fig. 1.**
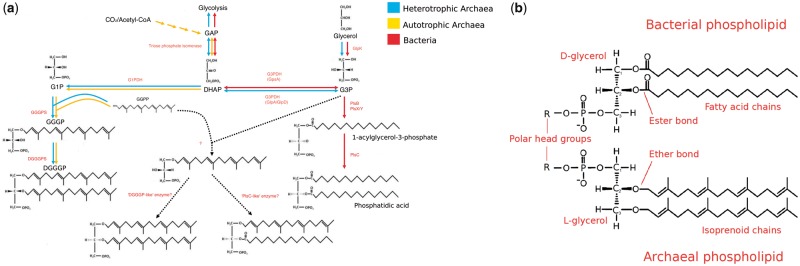
—(*a*) The canonical ether/ester biosynthetic pathways in Archaea and Bacteria and how they relate to glycerol metabolism. Based on figure 1 from Villanueva et al. (2017). Archaeal pathways in blue and yellow (blue = heterotrophic Archaea and yellow = autotrophic Archaea), bacterial pathway in red. Hypothetical biosynthetic pathway, as suggested by Villanueva et al. (2017), in dashed lines. (*b*) Composition of bacterial and archaeal phospholipids. In Archaea, glycerol-1-phosphate (G1P) is synthesized from dihydroxyacetone phosphate (DHAP) using the enzyme glycerol-1-phosphate dehydrogenase (G1PDH). The first and second isoprenoid chains (GGGPs) are added by geranylgeranylglyceryl phosphate synthase (GGGPS) and digeranylgeranylglyceryl phosphate synthase (DGGGPS), respectively. In Bacteria, glycerol-3-phosphate (G3P) is synthesized by glycerol-3-phosphate dehydrogenase (G3PDH) from DHAP. There are two forms of this enzyme, GpsA and GlpA/GlpD, encoded by the *gps* and *glp* genes, respectively. G3P may also be produced from glycerol by glycerol kinase (GlpK). In certain Bacteria, such as Gammaproteobacteria, the first fatty-acid chain is added by a version of glycerol-3-phosphate acyltransferase called PlsB. Other Bacteria, including most gram-positive bacteria, use a system which includes another glycerol-3-phosphate acyltransferase, PlsY, in conjunction with the enzyme PlsX (Yao and Rock 2013; Parsons and Rock 2013). The second fatty-acid chain is attached by 1-acylglycerol-3-phosphate *O*-acyltransferase (PlsC).

Archaea and Bacteria form the two primary domains of life (reviewed in Williams et al. 2013). Although similarities in their fundamental genetics and biochemistry, and evidence of homology in a near-universally conserved core of genes (Weiss et al. 2016) strongly suggest that Archaea and Bacteria descend from a universal common ancestor (LUCA), they also differ in ways that have important implications for the early evolution of cellular life. These differences include DNA replication (Kelman and Kelman 2014), transcription (Bell and Jackson 1998), DNA packaging (Reeve et al. 1997), and cell wall compositions (Kandler 1995). One striking difference is in the phospholipid composition of the cell membranes (fig. 1), which is particularly important for understanding the origin of cellular life. Canonically, Archaea have isoprenoid chains attached to a glycerol-1-phosphate (G1P) backbone via ether bonds and can have either membrane spanning or bilayer-forming phospholipids (Lombard et al. 2012a). Most Bacteria, as well as eukaryotes, classically have acyl (fatty-acid) chains attached to a glycerol-3-phosphate (G3P) backbone via ester bonds and form bilayers (Lombard et al. 2012a), although a number of exceptions have been documented (Sinninghe Damsté et al. 2002, 2007; Weijers et al. 2006; Goldfine 2010). Archaeal and bacterial phospholipids are synthesized by nonhomologous enzymes via different biosynthetic pathways (fig. 1). This so-called “lipid divide” (Koga 2011) raises some important questions regarding the early evolution of cellular life, including the nature of the membrane phospholipids present in LUCA and the number of times cell membranes have evolved.

The observation that phospholipid biosynthesis in Bacteria and Archaea is nonhomologous has motivated various hypotheses on the nature of LUCA’s membrane. The likely presence of some genes for lipid biosynthesis (Lombard and Moreira 2011; Lombard et al. 2012a; Koga 2014; Weiss et al. 2016) and, in particular, a membrane-bound ATPase (Sojo et al. 2014; Weiss 2016) in reconstructions of LUCA’s genome implies that LUCA possessed a membrane, although its properties may have been somewhat different to those of modern, ion-tight prokaryote cell membranes (Lombard et al. 2012a; Koga 2014; Sojo et al. 2014). It has also been suggested that LUCA may have had a heterochiral membrane (Wächtershäuser 2003), with later independent transitions to homochirality in Bacteria and Archaea, driven by increased membrane stability. However, the available experimental evidence—including the recent engineering of an *Escherichia coli* cell with a heterochiral membrane (Caforio et al. 2018)—suggests that homochiral membranes are not necessarily more stable than heterochiral ones (Fan et al. 1995; Shimada and Yamagishi 2011; Caforio et al. 2018), requiring some other explanation for the loss of ancestral heterochirality.

Despite the importance of the lipid divide for our understanding of early cellular evolution, membrane phospholipid stereochemistry of the glycerol moiety has been directly determined for a surprisingly limited range of Bacteria and Archaea. Since the initial full structural characterization of archaeol by Kates (1977), most subsequent studies of ether membrane lipids have assumed their stereochemistry while focusing on other aspects of their structure. Those studies that have determined the glycerol stereochemistry of membrane lipids (i.e., Sinninghe Damsté et al. 2002; Weijers et al. 2006) are largely consistent with the idea that it is a conserved difference between Bacteria and Archaea. Nonetheless, there is evidence that some Bacteria can make G1P-linked ether lipids. For example, the model bacterium *Bacillus subtilis* has been shown to possess homologs of archaeal glycerol-1-phosphate dehydrogenase (G1PDH) and geranylgeranylglyceryl phosphate synthase (GGGPS) (Guldan et al. 2008, 2011). These enzymes allow *B. subtilis* to synthesize a typically archaeal ether link between G1P and HepPP, resulting in a lipid with archaeal characteristics, although there is no evidence that these archaeal-like lipids are used to make phospholipids or are incorporated into the *B. subtilis* membrane.

Apart from stereochemistry, other characteristics of membrane phospholipids appear to be more variable, showing a mixture of archaeal and bacterial features. For example, the plasmalogens of animals and anaerobic Bacteria include an ether bond (Goldfine 2010). Branched glycerol dialkyl glycerol tetra-ether lipids found in the environment have bacterial stereochemistry and branched rather than isoprenoidal alkyl chains, but they also contain ether bonds and span the membrane, as observed for canonical archaeal lipids (Schouten et al. 2000; Weijers et al. 2006). These branched glycerol dialkyl glycerol tetra-ethers are particularly abundant in peat bogs and were thought to be produced by Bacteria as adaptations to low pH environments (Weijers et al. 2006; Sinninghe Damsté et al. 2007), but are now known to occur in a wide range of soils and aquatic settings (Schouten et al. 2013). The enzymes responsible for their synthesis are currently unknown. On the other side of the “lipid divide,” some Archaea have been shown to produce membrane lipids with fatty-acid chains and ester bonds (Gattinger et al. 2002). The biosynthetic pathways for all of these mixed-type membrane lipids remain unclear. However, given the frequency with which prokaryotes undergo horizontal gene transfer (Garcia-Vallvé et al. 2000), one possibility is that these mixed biochemical properties reflect biosynthetic pathways of mixed bacterial and archaeal origin.

A number of previous studies have investigated the evolutionary origins of phospholipid biosynthesis genes in Bacteria and Archaea using phylogenetic approaches, in order to test hypotheses about the nature of membranes in the earliest cellular life-forms (Peretó et al. 2004; Koga 2014; Yokobori et al. 2016; Villanueva et al. 2017). In this study, we build upon that work by performing comprehensive phylogenetic analyses for the core phospholipid biosynthesis genes in Bacteria and Archaea: the enzymes that establish membrane lipid stereochemistry and attach the two carbon chains to the glycerol phosphate backbone (fig. 1), as the histories of these enzymes are key to understanding the evolution of membrane biosynthesis and stereochemistry. Our analyses take advantage of the wealth of new genome data from environmental prokaryotes that has become available recently, and we employ new approaches for rooting single-gene trees in order to circumvent some of the difficulties inherent in traditional outgroup rooting for anciently diverged genes. Our results agree with previous work in suggesting that LUCA likely possessed a cell membrane. Our rooted gene trees indicate that transfers of lipid biosynthetic genes from Archaea to Bacteria have occurred more frequently in evolution, particularly during the early diversification of the two domains.

## Materials and Methods

### Sequence Selection

For Archaea, we selected 43 archaeal genomes, sampled evenly across the archaeal tree. We took corresponding archaeal G1PDH, geranylgeranylglyceryl phosphate synthase (GGGPS), and digeranylgeranylglyceryl phosphate synthase (DGGGPS) amino acid sequences from the data set of Villanueva et al. (2017) and performed BlastP searches to find these sequences in genomes not included in that data set. For Bacteria, we selected 64 bacterial genomes, sampled so as to represent the known genomic diversity of bacterial phyla (Hug et al. 2016). We used GpsA, GlpA/GlpD, and GlpK sequences from Yokobori et al. (2016) and performed BlastP searches to find those sequences in bacterial species not in their data set. For PlsC and PlsY, we took the corresponding sequences from Villanueva et al. (2017) and performed BlastP searches to find these sequences in the remaining genomes. For PlsB and PlsX, we searched for the respective terms in the gene database on the NCBI website, and upon finding well-verified occurrences, performed BlastP searches to find the corresponding amino acid sequences in the remaining genomes. We then used BlastP to look for bacterial orthologs of the archaeal enzymes and vice versa. We selected sequences that had an *E*-value of less the 10e-7 and at least 50% coverage. Accession numbers for sequences used are provided in supplementary table 5, Supplementary Material online.

### Phylogenetics

The sequences were aligned in mafft (Katoh et al. 2002) using the –auto option and trimmed in BMGE (Criscuolo and Gribaldo 2010) using the BLOSUM30 model, which is most suitable for anciently diverged genes. To construct gene trees from our amino acid sequences, we first selected the best-fitting substitution model for each gene according to its Bayesian Information Criterion score using the model selection tool in IQ-Tree (Nguyen et al. 2015). For all the genes we analyzed, the best-fitting model was a mixture model combining the Le and Gascuel (LG) exchangeability matrix (Le and Gascuel 2008) with site-specific composition profiles (the C40, C50, and C60 models [Lartillot and Philippe 2004; Le et al. 2008]) to accommodate across-site variation in the substitution process. LG + C60 was used for G1PDH, DGGGP, GpsA, GlpA/GlpD, GlpK, and PlsC. LG + 50 was used for PlsY. LG + C40 was used for GGGPS. A discretized Gamma distribution (Yang 1994) with four rate categories was used to model across-site rate variation. The trees were inferred with their respective models in PhyloBayes (Lartillot and Philippe 2004; Lartillot et al. 2007); convergence was assessed using the bpcomp and tracecomp programs (maxdiff < 0.1; effective sample sizes > 100), as recommended by the authors. We additionally inferred maximum likelihood (ML) trees in IQ-Tree using the LG + C60 model for each enzyme for comparison. We used heads-or-tails (Landan and Graur 2007) to assess the impact of alignment uncertainty: starting with the reversed alignments, we used the same phylogenetics pipeline as described above. Further testing was carried out by removing the metagenomic data from G1PDH, GGGPS, DGGGPS, GpsA, GlpA/GlpD, and GlpK, creating new alignments as described above, and inferring trees from these alignments in IQ-Tree using the LG + C60 model. We did not remove metagenomic data for PlsC or PlsY, as all of the archaeal sequences for these trees are derived from metagenome bins. In some cases, our trees included highly divergent sequences (sometimes forming distinct clades); we checked the *E*-values for these hits, and if they were close to or at the 10e-7 cut-off, they were removed and the analyses were rerun.

The trees were rooted with an uncorrelated lognormal relaxed molecular clock (RMC), using the LG model with a discretized Gamma distribution (Yang 1994) with four rate categories, and a Yule tree prior (Stadler 2009; Hartmann et al. 2010) in BEAST (Drummond and Rambaut 2007; Drummond et al. 2012). We also rooted the trees using minimal ancestor deviation (MAD) rooting (Tria et al 2017). We used two complementary methods: root posterior probabilities averaged over the trees sampled during the Bayesian molecular clock analysis using RootAnnotator (Calvignac-Spencer et al. 2014), and the ambiguity index (AI) implemented in MAD. The AI is defined as the ratio of the MAD value to the second smallest value. “Ties,” that is, where two or more competing root positions with equal deviations, would obtain a score of 1, with smaller values obtained in proportion to the relative quality of the best root position. See supplementary tables 3 and 4, Supplementary Material online, for AI and MAD scores.

For G1PDH, GpsA, and GlpA/GlpD, we also rooted using a subsample of the outgroup sequences used by Yokobori et al. (2016). The outgroups used were two sequences annotated as 3-dehydroquinate synthase, five as glycerol dehydrogenase, and five as alcohol dehydrogenase for G1PDH; six sequences annotated as hydroxyacyl-CoA dehydrogenase and six as uridine diphosphoglucose 6-dehydrogenase sequences for GpsA; and 12 sequences annotated as flavin adenine dinucleotide dependen oxidoreductase for GlpA/GlpD. All three of these trees were inferred under the LG + C60 model to directly compare to the unrooted trees. Trees were also inferred from best-fit models selected in IQTree (LG + C60 for G1PDH and GlpA/GlpD and LG + C50 for GpsA).

Eukaryotic orthologs of prokaryotic phospholipid biosynthesis genes (GlpA/GlpD, GpsA, and PlsC) were identified by performing BlastP searches on 35 eukaryotic genomes from across eukaryotic diversity using *Homo sapiens* query as the sequence in each case, selecting sequences with an *E*-value of 10e-7 or less, and at least 50% coverage. We then performed model testing in IQTree and inferred trees in PhyloBayes using the selected substitution model (LG + C60 for PlsC and LG + C50 for GlpA/GlpD and GpsA).

All sequences, alignments and trees referred to in this study can be obtained from 10.6084/m9.figshare.6210137.

## Results and Discussion

### Distribution of Core Phospholipid Biosynthesis Genes

We performed BlastP searches for the enzymes of the canonical archaeal and bacterial lipid biosynthesis pathways (fig. 1) against all archaeal and bacterial genomes in the NCBI nr database. Our BLAST searches revealed homologs for all of the core phospholipid biosynthesis genes of both pathways in both prokaryotic domains, with the exception of bacterial enzymes PlsB and PlsX, which we did not find in Archaea. Orthologs of the canonical archaeal genes are particularly widespread in many bacterial lineages (table 1). Of the 52 bacterial phyla surveyed, 8 had no orthologs of the archaeal genes (table 1, indicated in red). Six phyla have orthologs of all three archaeal genes distributed across various genomes (table 1, indicated in yellow and green). Of these phyla, Firmicutes (genera *Bacillus* and *Halanaerobium*), Actinobacteria (genus *Streptomyces*), and Fibrobacteres (genera *Chitinispirillum* and *Chitinivibrio*) contain species which have all three genes in their genomes (table 1, indicated in green). Based on the presence of all three core biosynthetic genes, and given their recognized role in the synthesis of archaeallike lipid components in *B. subtilis* (Guldan et al. 2008, 2011), members of Firmicutes, Actinobacteria, and Fibrobacteres lineages of Bacteria may be capable of making archaeallike lipids, although we cannot determine if these are used in the production of membrane phospholipids. Of the 12 FCB group (Fibrobacteres, Chlorobi, Bacteroidetes and related lineages) phyla we surveyed, all 12 have GGGPS and DGGGPS orthologs, but only Fibrobacteres and Cloacimonetes have G1PDH orthologs (see fig. 1 for overview of pathway). In these species lacking G1PDH, it is unclear whether GGGPS and DGGGPS are active and if so, what they are used for; one possibility is that they catalyze the reverse reaction, catabolizing archaeal lipids as an energy source. However, a very recent report (Villanueva et al. 2018) has shown that the GGGPS and DGGGPS genes from one FCB lineage, Cloacimonetes, support the production of archaeal-type membrane phospholipids and a mixed membrane when heterologously expressed in *E. coli*. This suggests that both *E. coli* and perhaps Cloacimonetes have an alternative, as yet unknown mechanism for making G1P, and that some FCB members may have mixed archaeal and bacterial membranes.

**Table 1:** Distribution of Phospholipid Biosynthesis Genes in Bacterial and Archaeal Phyla

Domain	Superphylum	Phylum	Class	G1PDH	GGGPS	DGGGPS	GpsA	GlpA/GlpD	GlpK	PlsC	PlsY
Archaea		Euryarchaeota	Archaeoglobi	**✓**	**✓**	**✓**	**✓**	**✓**	**✓**		
			Halobacteria	**✓**	**✓**	**✓**		**✓**	**✓**		
			Methanobacteria	**✓**	**✓**	**✓**	**✓**				
			Methanococci	**✓**	**✓**	**✓**					
			Methanomicrobia	**✓**	**✓**	**✓**	**✓**	**✓**			
			Thermococci	**✓**	**✓**	**✓**		**✓**	**✓**		
			Thermoplasmatales	**✓**	**✓**	**✓**	**✓**	**✓**	**✓**	**✓**	
	TACK	Aigarchaeota		**✓**	**✓**	**✓**					
		Crenarchaeota		**✓**	**✓**	**✓**	**✓**	**✓**	**✓**		
		Korarchaeota		**✓**	**✓**	**✓**		**✓**	**✓**		
		Thaumarchaeta		**✓**	**✓**	**✓**					
	Asgard	Heimdallarchaeota		**✓**	**✓**	**✓**				**✓**	**✓**
		Lokiarchaeota		**✓**	**✓**	**✓**		**✓**	**✓**	**✓**	**✓**
		Odinarchaeota		**✓**	**✓**	**✓**					
		Thorarchaeota		**✓**	**✓**	**✓**					**✓**
	DPANN	Aenigmarchaeota		**✓**	**✓**	**✓**					
		Diapherotrites (GW2011_AR10/DUSEL3)			**✓**	**✓**	**✓**				**✓**
		Micrarchaeota (incl. Macid)		**✓**	**✓**	**✓**					
		Nanoarchaeota									
		Nanohaloarchaeota									
		Pacearchaeota									**✓**
		Parvarchaeota						**✓**	**✓**		
		Woesearchaeota					**✓**		**✓**	**✓**	**✓**
Bacteria		Acidobacteria		**✓**			**✓**		**✓**	**✓**	**✓**
		Actinobacteria		**✓**	**✓**	**✓**	**✓**	**✓**	**✓**	**✓**	**✓**
		Aminicenantes		**✓**			**✓**	**✓**	**✓**		**✓**
		Aquificae					**✓**	**✓**	**✓**	**✓**	**✓**
		Armatimonadetes		**✓**			**✓**	**✓**	**✓**	**✓**	**✓**
		Candidate division KSB1			**✓**	**✓**	**✓**	**✓**	**✓**	**✓**	**✓**
		Candidate division NC10					**✓**	**✓**	**✓**	**✓**	**✓**
		Candidate division TA06		**✓**	**✓**	**✓**	**✓**			**✓**	**✓**
		Candidate division WOR-3		**✓**	**✓**	**✓**	**✓**	** **	** **	**✓**	**✓**
		Candidatus Edwardsbacteria			**✓**	**✓**	**✓**			**✓**	**✓**
		Candidatus Handelsmanbacteria			**✓**	**✓**		**✓**	**✓**	**✓**	**✓**
		Candidatus Kerfeldbacteria		**✓**	**✓**		**✓**		**✓**	**✓**	**✓**
		Candidatus Magnetoovum		**✓**	**✓**		**✓**			**✓**	**✓**
		Candidatus Raymondbacteria		**✓**	**✓**	**✓**	**✓**	**✓**	**✓**	**✓**	**✓**
		Chloroflexi		**✓**	**✓**	**✓**	**✓**	**✓**	**✓**	**✓**	**✓**
		Chrysiogenetes		**✓**	**✓**		**✓**			**✓**	**✓**
		Cyanobacteria		**✓**	**✓**	**✓**	**✓**	**✓**	**✓**	**✓**	**✓**
		Deferribacterales					**✓**	**✓**	**✓**	**✓**	**✓**
		Deinococcus-Thermus				**✓**	**✓**	**✓**	**✓**	**✓**	**✓**
		Dictyoglomi		**✓**			**✓**		**✓**	**✓**	**✓**
		Elusimicrobia			**✓**	**✓**	**✓**	**✓**	**✓**	**✓**	**✓**
		Firmicutes		**✓**	**✓**	**✓**	**✓**	**✓**	**✓**	**✓**	**✓**
		Fusobacteria			**✓**		**✓**	**✓**	**✓**		**✓**
		Melainabacteria		**✓**	**✓**		**✓**	**✓**	**✓**	**✓**	**✓**
		Nitrospinae					**✓**	**✓**		**✓**	**✓**
		Nitrospirae		**✓**			**✓**	**✓**	**✓**	**✓**	**✓**
		Parcubacteria			**✓**	**✓**	**✓**			**✓**	**✓**
		Proteobacteria		**✓**	**✓**	**✓**	**✓**	**✓**	**✓**	**✓**	**✓**
		Rhodothermaeota			**✓**	**✓**	**✓**		**✓**	**✓**	**✓**
		Spirochaetes		**✓**	**✓**		**✓**	**✓**	**✓**	**✓**	**✓**
		Synergistetes		**✓**			**✓**		**✓**	**✓**	**✓**
		Tenericutes					**✓**	**✓**	**✓**	**✓**	**✓**
		Thermobaculum					**✓**	**✓**	**✓**	**✓**	**✓**
		Thermodesulfobacteria					**✓**			**✓**	**✓**
		Thermotogae		**✓**	**✓**		**✓**	**✓**	**✓**	**✓**	**✓**
		TMED			**✓**					**✓**	**✓**
	FBC	Bacteroidetes			**✓**	**✓**	**✓**		**✓**	**✓**	**✓**
		Caldithrix		**✓**	**✓**	**✓**	**✓**	**✓**		**✓**	**✓**
		Candidatus Marinimicrobia			**✓**	**✓**	**✓**	**✓**	**✓**	**✓**	**✓**
		Candidatus Kryptonium			**✓**	**✓**	**✓**			**✓**	**✓**
		Candidatus Kryptobacter			**✓**	**✓**	**✓**			**✓**	**✓**
		Candidate division Zixibacteria			**✓**	**✓**	**✓**			**✓**	**✓**
		Chlorobi			**✓**	**✓**	**✓**	**✓**	**✓**	**✓**	**✓**
		Cloacimonetes		**✓**	**✓**	**✓**	**✓**	**✓**	**✓**	**✓**	**✓**
		Fibrobacteres		**✓**	**✓**	**✓**	**✓**	** **	** **	**✓**	**✓**
		Gemmatimonadetes			**✓**	**✓**	**✓**	**✓**	**✓**	**✓**	**✓**
		Ignavibacteria			**✓**	**✓**	**✓**			**✓**	**✓**
		Latescibacteria			**✓**	**✓**	**✓**		**✓**		**✓**
	PVC	Chlamydia					**✓**		**✓**	**✓**	**✓**
		Lentisphaerae		**✓**		**✓**	**✓**	**✓**	**✓**	**✓**	**✓**
		Planctomycetes		**✓**			**✓**	**✓**	**✓**	**✓**	**✓**
		Verrumicrobia					**✓**		**✓**	**✓**	**✓**

Note.—Ticks represent phyla (class level for Euryarchaeota) with at least one genome which has a sequence for the corresponding gene. Bacterial phyla where all three archaeal genes are found are indicated in yellow and green. Those bacterial phyla where all three archaeal genes are found within the same genome in at least one case are indicted in green. Those bacterial phyla with no archaeal genes are found are indicated in red. It should be noted that in the case of environmental lineages, the lack of a tick may not represent absence of genes, given that these represent metagenomics bins, and the lack of said genes may be due to missing data. FCB are Fibrobacteres, Chlorobi, and Bacteroidetes and related lineages. PVC are Planctomycetes, Verrucomicrobia, and Chlamydiae and related lineages. TACK are Thaumarchaeota, Aigarchaeota, Crenarchaeota, and Korarchaeota. DPANN include Diapherotrites, Parvarchaeota, Aenigmarchaeota, Nanoarchaeota, and Nanohaloarchaeota, as well as several other lineages.

Orthologs of the canonical bacterial genes are less widespread in Archaea (table 1). Of all the genomes surveyed, none contained all homologs. Of the 17 phyla shown in table 1, 8 had no bacterial homologs in any of their genomes. Orthologs of GpsA, GplA/GlpD, and Gpk are found in at least one genome of each of the major archaeal clades (Euryarchaeota, Thaumarchaeota, Aigarchaeota, Crenarchaeota, and Korarchaeota [TACK], Asgardarchaeota, and Diapherotrites, Parvarchaeota, Aenigmarchaeota, Nanoarchaeota, and Nanohaloarchaeota, as well as several other lineages [DPANN] [Williams et al. 2017]). However, they appear sporadically. Within Euryarchaeota, of the seven classes surveyed, GpsA and GlpK appear in the genomes of four and GlpA/GlpD in five. Within the TACK superphylum, GlpA/GlpD and GlpK appear in Crenarchaeota and Korarchaeota, whereas GpsA appears only in a single crenarchaeote genome (*Thermofilum*). GpsA and GlpK are also found in at least one genome in two of the eight DPANN phyla surveyed (Woesearchaeota and GW2011, and Woesearchaeota and Parvarchaeota, respectively), whereas GlpA/GlpD is found in a single parvarchaeote genome (Candidatus Parvarchaeum acidiphilum ARMAN-4). Within the Asgardarchaeota superphylum, no orthologs for GpsA are found, and only one of the genomes (*Lokiarchaeum* sp. GC14_75) has GlpA/GlpD or GlpK. PlsC and PlsY are more restricted, being found mainly in environmental lineages within Euryarchaeota (Marine Groups II/III, all in class Thermoplasmatales), DPANN, and Asgardarchaeota (table 1).

### Early Origins of Archaeal-Type Membrane Phospholipid Biosynthesis Genes in Bacteria

To investigate the evolutionary histories of membrane phospholipid biosynthesis, we inferred Bayesian single-gene phylogenies from the amino acid alignments using PhyloBayes 4.1 (Lartillot and Philippe 2004; Lartillot et al. 2007). We selected the best-fitting substitution model for each gene according to its Bayesian Information Criterion score using the model selection tool in IQ-Tree (Nguyen et al. 2015). We used two complementary approaches to root these single-gene trees: a RMC in BEAST (Drummond and Rambaut 2007; Drummond et al. 2012), and the recently described MAD rooting method of Tria et al. (2017). The MAD algorithm finds the root position that minimizes pairwise evolutionary rate variation, averaged over all pairs of taxa in the tree. Many of our single-gene trees were poorly resolved, and we wanted to account for topological uncertainty in our root estimates. To do so, we used two complementary methods: root posterior probabilities averaged over the trees sampled during the Bayesian molecular clock analysis, and the AI implemented in MAD, which is defined by Tria et al. (2017) as the ratio of the MAD value to the second smallest value (for AI scores, see supplementary table 3, Supplementary Material online). For the genes for which an outgroup was available (G1PDH, GpsA, and GlpA/GlpD, following Yokobori et al. 2016), we compared our results to traditional outgroup rooting. For more details, see Materials and Methods.

**Fig. 2.**
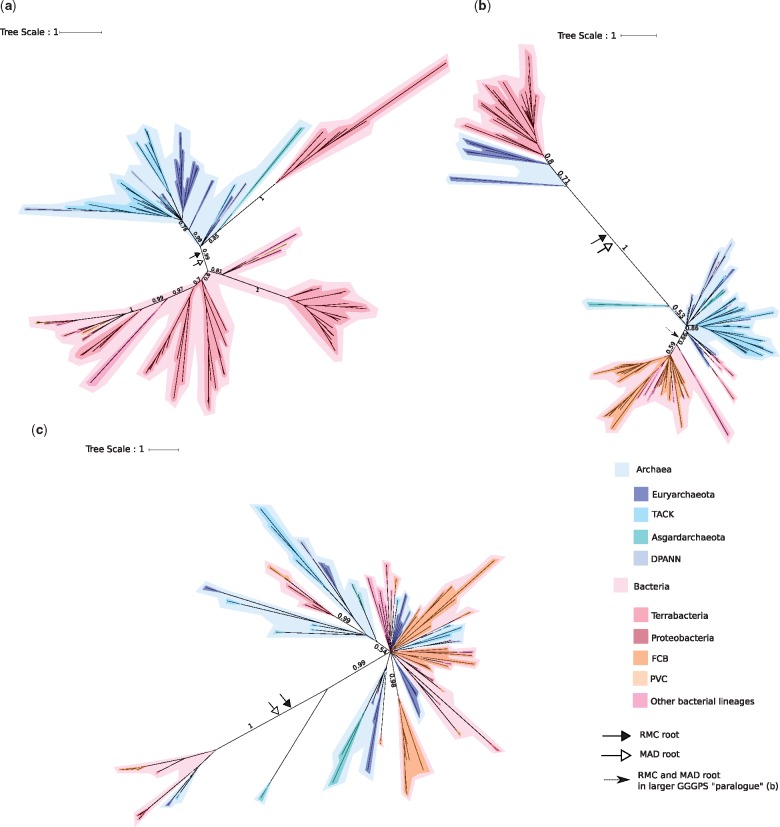
—Bayesian consensus trees of archaeal enzymes. Support values are Bayesian posterior probabilities. The black arrow and the white arrow indicate the modal root positions obtained using the RMC and MAD approaches, respectively. The dashed arrow indicates the RMC and MAD roots for the larger GGGPS subclade. Archaea in blue-tones and Bacteria in red/pink-tones. (*a*) G1PDH tree (111 sequences and 190 positions) inferred under the best-fitting LG + C60 model. (*b*) GGGPS tree (133 sequences and 129 positions) inferred under the best-fitting LG + C40 model. (*c*) DGGGPS tree (97 sequences and 119 positions) inferred under the best-fitting LG + C60 model. Terrabacteria are Firmicutes, Actinobacteria, Cyanobacteria, Chloroflexi, and related lineages. FCB are Fibrobacteres, Chlorobi, and Bacteroidetes and related lineages. PVC are Planctomycetes, Verrucomicrobia, and Chlamydiae and related lineages. TACK are Thaumarchaeota, Aigarchaeota, Crenarchaeota, and Korarchaeota. DPANN include Diapherotrites, Parvarchaeota, Aenigmarchaeota, Nanoarchaeota, and Nanohaloarchaeota, as well as several other lineages. For full trees, see supplementary figures 1–4, Supplementary Material online. For full unrooted trees, see supplementary figures 16–18, Supplementary Material online.

G1PDH is the enzyme that establishes phospholipid stereochemistry in Archaea. Interestingly, the majority of the bacterial G1PDH orthologs do not appear to be recent horizontal acquisitions from Archaea, but instead form a deep-branching clan (Wilkinson et al. 2007) (PP = 1), resolved as sister to an archaeal lineage clan (fig. 2*a*). The relationships within the clans are poorly resolved. The root position that receives the highest posterior support in the RMC analysis is that between the archaeal and bacterial clans, with a marginal posterior probability of 0.68 (supplementary table 1, Supplementary Material online). This is substantially higher than the next most probable position, which places the root within the Bacteria with a posterior probability of 0.1. When rooted using MAD, the same root between the bacterial and archaeal clans is recovered with a marginal posterior probability of 0.62, also substantially higher than the next most probable root of 0.1. Rooting single-gene trees can prove difficult, and this uncertainty is captured in the low root probabilities inferred using both the RMC and MAD methods. However, these analyses can be used to exclude the root from some regions of the trees with a degree of certainty. In the case of G1PDH, a post-LUCA origin of the gene would predict a root on the archaeal stem or within the Archaea. In our analyses, no such root position has a significant probability (i.e., PP > 0.05), and therefore the root is highly unlikely to be within the Archaea. This is similar to topologies recovered by Peretó et al. (2004) and Carbone et al. (2015). The bacterial clan mainly comprises sequences from Firmicutes and Actinobacteria, with most of the other Bacteria grouping together in a single, maximally supported (PP = 1) lineage suggestive of recent horizontal acquisition from the Firmicutes/Actinobacteria clade, followed by further HGT.

This root position is consistent with two scenarios that we cannot distinguish based on the available data. One possibility is an early transfer of G1PDH from stem Archaea into Bacteria, either into the bacterial stem lineage with subsequent loss in later lineages, or into the ancestor of Actinobacteria and Firmicutes, with subsequent transfers to other Bacteria. Alternatively, G1PDH could have already been present in LUCA, and was subsequently inherited vertically in both Archaea and Bacteria, followed by loss in later bacterial lineages. The Firmicute sequences within the archaeal clade appear to be a later transfer into those Firmicutes, apparently from Thorarchaeota.

GGGPS attaches the first isoprenoid chain to G1P. Phylogenetic analysis of GGGPS (fig. 2*b*) evidenced two deeply divergent paralogs, with the tree confidently rooted between them using both the RMC (PP = 0.99) and MAD methods (PP = 1) (supplementary table 1, Supplementary Material online); resolution within each of the paralogs was poor. The recovery of two distinct paralogs has been noted in several previous studies (Nemoto et al. 2003; Boucher et al. 2004; Lombard et al. 2012b; Peterhoff et al. 2014). One of these paralogs comprises sequences from some Euryarchaeota (including members of the Haloarchaea, Methanomicrobia, and Archaeoglobi), along with Firmicutes and Actinobacteria. The other paralog comprises sequences from the rest of the Archaea—including other Euryarchaeota—and a monophyletic bacterial clade largely consisting of members of the FCB lineage. Taken with the root position between the two paralogs, the tree topology implies an ancestral duplication followed by sorting out of the paralogs and multiple transfers into Bacteria. Because genes from both GGGPS paralogous clades have been experimentally characterized as geranylgeranylglyceryl phosphate synthases (Nemoto et al. 2003; Boucher et al 2004), it appears that this activity was already present in LUCA before the radiation of the bacterial and archaeal domains. Payandeh et al. (2006) has suggested, however, that the firmicute sequences (which comprise the majority of the sequences in the smaller paralog) are used in teichoic acid synthesis. In this case, two apparently diverging paralogs may be an artifact due to changes in the sequences during neofunctionalization. Lombard et al. (2012b), who also find two divergent homologs, and homologs in a large diversity of FCB bacteria (mostly Bacteroidetes), suggest that one of these homologs was likely present in the last archaeal common ancestor, whereas the bacterial sequences were likely horizontal transfers. To improve resolution among the deeper branches of the tree, we inferred an additional phylogeny focusing just on the larger of the two clades (supplementary fig. 3, Supplementary Material online). The root of this subtree fell between a clade of monophyletic Bacteria and a clade of Archaea in which six bacterial sequences were interleaved, perhaps as the result of later gene transfer (PP = 0.8 for the root split, much higher than the next most likely root, within the Bacteria, with PP = 0.07). This tree might be interpreted as gene presence in LUCA, followed by some more recent transfers from Archaea to Bacteria. Given that this gene is a hallmark of archaeal membrane phospholipid biosynthesis, our data do not exclude the possibility of a very early gene transfer from the archaeal stem to Bacteria, prior to the radiation of the archaeal domain.

DGGGPS attaches the second isoprenoid chain to G1P. DGGGPS is present in all sampled Archaea, with the exception of three of the DPANN metagenome bins. Although the DGGGPS tree is poorly resolved (fig. 2*c*), both the RMC and MAD root the tree between the same two clades (PP = 0.43 and 0.79, respectively) (supplementary table 1, Supplementary Material online). The smaller clade comprises mostly bacterial sequences from the Actinobacteria and FCB lineages, as well as two archaeal sequences (from the TACK and Euryarchaeota lineages). The larger clade contains sequences from a diversity of Bacteria, particularly FCB (also reported by Villanueva et al. 2018), as well as Archaea. DGGGPS is part of the UbiA protein superfamily, which are involved in a number of different biosynthetic pathways, including the production of photosynthetic pigments, and are therefore widely distributed in Bacteria, and are known to have undergone extensive HGT (Hemmi et al. 2004). Indeed, several of the sequences used in our analyses (and those in previous studies, such as Villanueva et al. 2017) are annotated on NCBI as other proteins within this superfamily (see supplementary table 5, Supplementary Material online). To distinguish orthologs of DGGGPS from other, distantly related members of the UbiA superfamily that might have different functions, we inferred an expanded phylogeny including our initial sequence set and sequences sampled from the other known UbiA subfamilies (supplementary fig. 25, Supplementary Material online). Surprisingly, this analysis indicated that the Thaumarchaeota lack an ortholog of the DGGGPS gene that other Archaea use to attach the second isoprenoid chain; the most closely related Thaumarchaeota sequences branch within another UbiA subfamily with high posterior support (PP = 0.99). Thaumarchaeota may be using this paralog to perform the same function, or may use another unrelated enzyme to catalyze this reaction. The wide distribution of this enzyme across both Archaea and Bacteria, and the occurrence of both domains on either side of the root, for both rooting methods, suggest either multiple transfers into Bacteria from Archaea, or that DGGGPS was present in LUCA and inherited in various archaeal and bacterial lineages, followed by many later losses in and transfers between various lineages.

In sum, our results of archaeal phospholipid biosynthesis genes suggest that there have been repeated, independent inter domain transfers of these genes from Archaea to Bacteria throughout the evolutionary history of life. Furthermore, our phylogenetic analyses do not exclude the possibility that the genes of the archaeal pathway were present in LUCA. If correct, this would imply that LUCA had the capability to make archaeal-type membrane phospholipids.

### Transfers of Bacterial Membrane Phospholipid Genes into Archaea

In contrast to our analyses of proteins of the classical archaeal pathway, phylogenies of proteins of bacterial-type membrane phospholipid biosynthesis pathways are more ambiguous and the root positions are not confidently resolved. Homologs of both forms of glycerol-3-phosphate dehydrogenase (G3PDH) and GlpK are broadly distributed in Archaea, however, these three enzymes are not exclusive to phospholipid synthesis and have been shown to be used in glycerol metabolism in some autotrophic Archaea (Nishihara et al. 1999). Of the enzymes thought to function exclusively in bacterial membrane phospholipid biosynthesis, we did not find any archaeal homologs for PlsB or PlsX, and archaeal PlsC and PlsY homologs are patchily distributed and are found only in metagenomic bins. It therefore seems unlikely that any of these genes function in membrane phospholipid synthesis in Archaea.

**Fig. 3.**
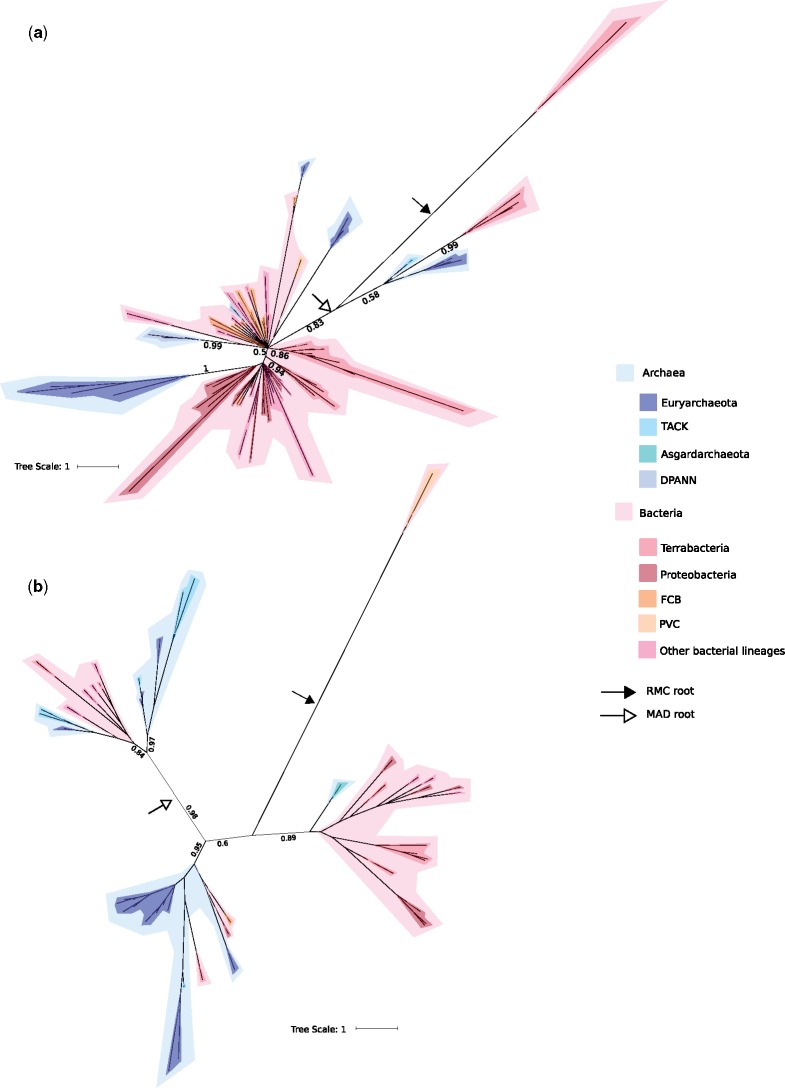
—Bayesian consensus trees of both G3PDH enzymes. Support values are Bayesian posterior probabilities. The black arrow and the white arrow indicate the modal root positions obtained using the RMC and MAD approaches, respectively. Archaea in blue-tones and Bacteria in red/pink-tones. (*a*) GpsA tree (84 sequences and 169 positions) inferred under the best-fitting LG + C60 model. (*b*) GlpA/GlpD tree (51 sequences and 199 positions) inferred under the best-fitting LG + C60 model. Terrabacteria are Firmicutes, Actinobacteria, Cyanobacteria, Chloroflexi, and related lineages. FCB are Fibrobacteres, Chlorobi, and Bacteroidetes and related lineages. PVC are Planctomycetes, Verrucomicrobia, and Chlamydiae and related lineages. TACK are Thaumarchaeota, Aigarchaeota, Crenarchaeota, and Korarchaeota. DPANN include Diapherotrites, Parvarchaeota, Aenigmarchaeota, Nanoarchaeota, and Nanohaloarchaeota, as well as several other lineages. For full trees, see supplementary figures 5 and 6, Supplementary Material online. For full unrooted trees, see supplementary figures 19 and 20, Supplementary Material online.

**Fig. 4.**
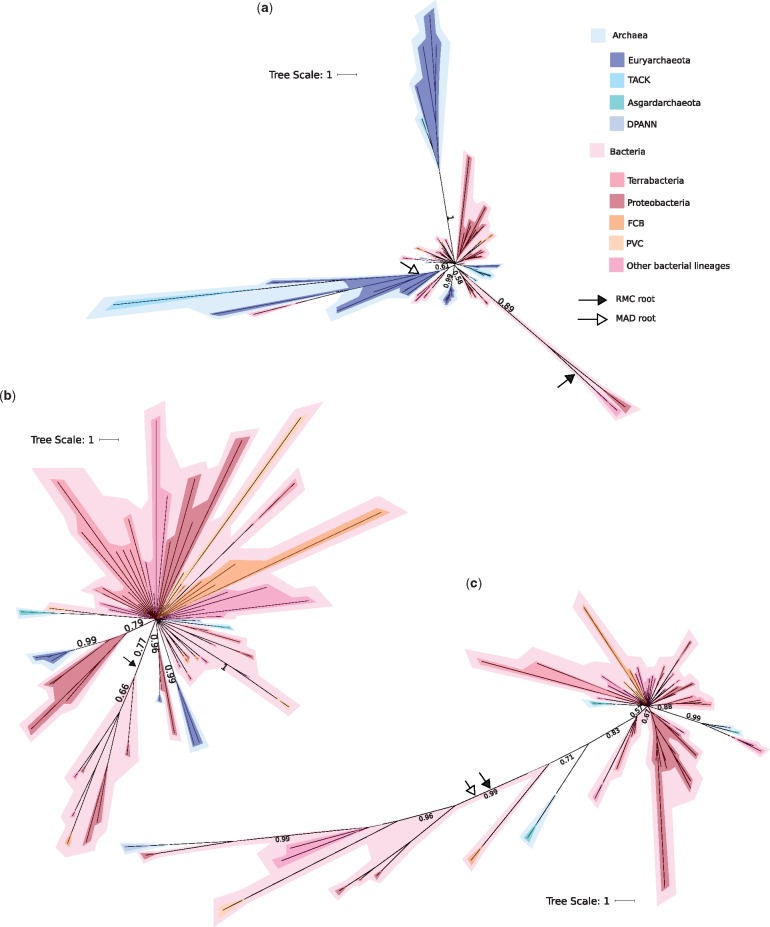
—Bayesian consensus trees of GlpK, PlsC, and PlsY enzymes. Support values are Bayesian posterior probabilities. The black arrow and the white arrow indicate the modal root positions obtained using the RMC and MAD approaches, respectively. Archaea in blue-tones and Bacteria in red/pink-tones. (*a*) GlpK tree (77 sequences and 363 positions) inferred under the best-fitting LG + C60 model. (*b*) PlsC tree (74 sequences and 57 positions) inferred under the best-fitting LG + C60 model. (*c*) PlsY tree (60 sequences and 104 positions) inferred under the best-fitting LG + C50 model. Terrabacteria are Firmicutes, Actinobacteria, Cyanobacteria, Chloroflexi, and related lineages. FCB are Fibrobacteres, Chlorobi, and Bacteroidetes and related lineages. PVC are Planctomycetes, Verrucomicrobia, and Chlamydiae and related lineages. TACK are Thaumarchaeota, Aigarchaeota, Crenarchaeota, and Korarchaeota. DPANN include Diapherotrites, Parvarchaeota, Aenigmarchaeota, Nanoarchaeota, and Nanohaloarchaeota, as well as several other lineages. For full trees, see supplementary figures 7–9, Supplementary Material online. For full unrooted trees, see supplementary figures 20–23, Supplementary Material online.

The root positions for each of the trees using both RMC and MAD have low posterior probabilities (supplementary table 1, Supplementary Material online), so that the exact root positions are unclear. *Gps* and *glp* are two genes that code for two forms of glycerol-3-phosphate (G3PDH), GpsA, and GlpA/GlpD, respectively, which establishes phospholipid stereochemistry in Bacteria. The deep relationships between the archaeal and bacterial sequences in the GpsA tree are poorly resolved (fig. 3*a*), while being better resolved for GlpA/GlpD (fig. 3*b*). The root position in both trees is poorly resolved for both rooting methods (supplementary table 1, Supplementary Material online). The highest marginal posterior probability for the root positions recovered in the GpsA tree are 0.31 and 0.59 and for the RMC and MAD, respectively, and 0.5 and 0.44, respectively, for GlpA/GlpD. The tree inferred for GlpK (glycerol synthase, which can synthesize G3P from glycerol [fig. 4*a*]), shows a similar pattern to the phylogenies of GpsA and GlpA/GlpD. Again, the root positions have low posterior support (0.47 and 0.34 for the RMC and MAD, respectively). However, in each case, there is evidence of recent transfers from Bacteria to Archaea, as we recover several distinct bacterial and archaeal clades with moderate to high support (0.8–1), as also reported by Villanueva et al. (2017). For all three of these enzymes, the differing root positions are resolved either within the Bacteria, or with bacterial and archaeal sequences on both sides of the root. This suggests that these enzymes may have been present in LUCA, or that the archaeal sequences are later transfers from Bacteria. Due to incongruence between the rooting methods and the low supports, our analyses do not robustly reject either of these scenarios.

PlsC and PlsY (which attach fatty acids to G3P) both have many fewer orthologs among archaeal genomes, all of which are derived from environmental samples (Embley and Martin 2006; Martin et al. 2015; Eme et al. 2017). Both trees are poorly resolved (fig. 4). Both are rooted within the Bacteria, with PlsC (fig. 4*b*) having the low posterior of 0.28 (with the next most likely, also within the Bacteria, being 0.1). The PlsY (fig. 4*c*) has a more certain root position, with a posterior of 0.57, and the next most probable being 0.1. For PlsY, MAD recovers the same root as the molecular clock, with a high posterior probability (0.85). When the PlsC tree is rooted using MAD, the root is resolved between two clades, which are not recovered in the inferred tree topology (see supplementary fig. 8, Supplementary Material online) and has a low posterior probability of 0.03. All of the archaeal homologs seem to be horizontal acquisitions from Bacteria.

### Sensitivity to Model Fitting Approach, Alignment Uncertainty, and the Inclusion of Metagenomic Sequences

The deep branches of our trees are in general poorly resolved, a problem that is sometimes encountered when inferring phylogenies for ancient single genes (Williams et al. 2011). We therefore performed sensitivity analyses to evaluate the robustness of our biological conclusions to some of the key decision points in our phylogenetic approach. Our focal analyses are Bayesian, so we also inferred trees using the same models in the maximum likelihood framework using IQ-Tree (see supplementary figs. 30–37, Supplementary Material online, for ML topologies, and supplementary table 3, Supplementary Material online, for MAD AI scores). The topologies were closely similar to the Bayesian trees, with the exception of some poorly support clades that are resolved in the ML tree but are not present in the Bayesian majority rule consensus tree. The root positions on the G1PDH, GGGPS, DGGGPS, GpsA, GlpA/GlpD, and PlsC ML trees were identical to the those on the Bayesian trees. The MAD root positions for GlpK and PlsY ML trees differ from the Bayesian trees, but in both cases the root positions are on adjacent branches and the changes do not substantially alter our interpretations (supplementary figs. 35–37, Supplementary Material online).

We evaluated the impact of alignment uncertainty on our results using heads-or-tails (Landan and Graur 2007). The reverse alignments were used to infer ML trees in IQ-Tree using the LG + C60 model. These were broadly congruent with the ML and Bayesian trees on the original alignments, with only minor topological differences in poorly resolved areas of the trees (supplementary figs. 38–45, Supplementary Material online). The root positions on the G1PDH, GGGPS, DGGGPS, GlpA/GlpD, GpsA, and PlsC ML trees were identical to the those on the Bayesian trees (supplementary figs. 38–40, 42, 44, Supplementary Material online). The MAD root positions for GlpK (supplementary fig. 43, Supplementary Material online) and PlsY (supplementary fig. 45, Supplementary Material online) ML trees differ for the Bayesian trees, but in the case of PlsY, the root positions is on an adjacent branch. The MAD root position for GlpK falls between a *Korarchaeum* sequence and the rest of the tree.

Due to errors in assembly, metagenome bins sometimes incorporate sequences from more than one underlying organismal genome (Parks et al. 2015). To evaluate whether some apparent gene transfers might be artifacts of metagenome assembly, we repeated our analyses without the inclusion of metagenome-derived sequences, where possible. We performed these analyses for G1PDH, GGGPS, DGGGPS, GpsA, GlpA/GlpD, and GlpK, but not for PlsC or PlsY, because all of the archaeal sequences for these trees are derived from metagenome bins (see supplementary table 5, Supplementary Material online). In the six cases where a reasonable comparison can be made, the topologies and roots of the trees were closely similar to those in the full analysis (supplementary figs. 46–52, Supplementary Material online).

These results suggest that, although our analyses do include substantial topological uncertainty, our overall conclusions are not driven by issues with alignment, metagenome-derived sequences, or the choice of model fitting approach (maximum likelihood or Bayesian).

### Comparing Outgroup and Outgroup-Free Rooting for Single-Gene Trees

Evolutionary interpretations typically depend on rooted trees, but rooting single-gene trees can prove difficult. The most widely used approach is to place the root on the branch leading to a predefined outgroup (Penny 1976). However, this can be challenging for ancient genes when closely related outgroups are lacking; either the outgroup method cannot be used at all, or else the long branch leading to the outgroup can induce errors in the ingroup topology (a phenomenon known as long branch attraction [LBA]; see Gouy et al. 2015).

In the case of phospholipid biosynthesis, some of the key genes belong to larger protein families whose other members, although distantly related, have conserved structures and related functions (Peretó et al. 2004). Several previous studies looking at the history of phospholipid biosynthetic genes have used these outgroups for rooting. Due in part to the difficulties of outgroup rooting for ancient genes, these studies have disagreed on the roots for some of these gene trees, leading to very different evolutionary conclusions. Our outgroup-free results are consistent with those of Peretó et al. (2004) and Carbone et al. (2015), but not with those of the recent study of Yokobori et al. (2016). Yokobori et al. used outgroups to root trees for G1PDH, G3PDH (both GpsA and GlpA/GlpD) and GlpK. Their root inferences differed from ours in that they found that bacterial G1PDH sequences formed a monophyletic group that branched from within Archaea, suggesting more recent horizontal transfer from Archaea to Bacteria, as opposed to transfer from stem Archaea or vertical inheritance from LUCA (fig. 2*a*). On the other hand, their analysis of GlpA/GlpD recovered Bacteria on one side of the root, and a clade of Bacteria and Archaea on the other. They interpreted this as evidence for the presence of GlpA/GlpD in LUCA, and therefore that LUCA would have had bacterial-type G3P membrane phospholipids.

Single-matrix models, such as those used by Yokobori et al. (2016), have been shown to be more susceptible to phylogenetic artifacts such as LBA than the profile mixture models used here (Lartillot et al. 2007). To investigate whether the differences in root inference between our analyses and those of Yokobori et al. (2016) might be the result of LBA, we performed outgroup rooting analysis on G1PDH, GpsA, and GlpA/GlpD, augmenting our data sets with a subsample of the outgroups used by Yokobori et al. and using the same models used to infer the unrooted trees (LG + C60 in each case). The resulting trees (supplementary figs. 10–12, Supplementary Material online) show different topologies when compared with the unrooted trees (supplementary figs. 16, 19, and 20, Supplementary Material online). This suggests that the long branch outgroup may be distorting the ingroup topology.

We also performed model testing in IQ-Tree and compared the fit of the chosen models to the models used by Yokobori et al. (see Material and Methods below). LG + C60 was selected for both G1PDH and GlpA/GlpD, whereas LG + C50 was selected for GpsA (supplementary fig. 24, Supplementary Material online). The results of these analyses indicate that the empirical profile mixture models which we have used here fit each of these alignments significantly better than the single-matrix models of Yokobori et al. (supplementary table 2, Supplementary Material online). However, even analyses under the best-fitting available models show distortion of the ingroup topology upon addition of the outgroup (supplementary figs. 10–12 and 24, Supplementary Material online), when compared with the unrooted topologies (supplementary figs. 16, 19, and 20, Supplementary Material online). In each case, we found the root in a different place to those recovered by Yokobori et al. In the G1PDH tree, we find Bacteria (Firmicutes) to be most basal, rather the Crenarchaeota found by Yokobori. In the case of GpsA, Yokobori et al. did not find compelling support for an origin in LUCA, but they did recover one archaeal lineage (the Euryarchaeota) at the base of the ingroup tree with low (bootstrap 48) support. Although our GpsA tree is also poorly resolved, we do not find evidence to support the basal position of the archaeal lineages, and therefore for the presence of GpsA in LUCA. For GlpA/GlpD, which Yokobori et al. trace back to LUCA due to the basal position of the archaeal sequences, the outgroup sequences did not form a monophyletic group, and were instead distributed throughout the tree (supplementary fig. 11, Supplementary Material online). Thus, analyses under the best-fitting available models did not support the presence of bacterial lipid biosynthesis genes in LUCA. Further, the distortion of the ingroup topologies suggests that these outgroups may not be suitable for root inference, at least given current data and methods. The RMC and the MAD methods have their own assumptions and limitations, but these results suggest that they may be useful for rooting trees in other contexts, either as part of a sensitivity test or when suitable outgroups are not available.

### Origin of Eukaryotic Membrane Phospholipid Biosynthesis Genes

Phylogenetics and comparative genomics suggest that eukaryotes arose from a symbiosis between an archaeal host cell and a bacterial endosymbiont that evolved into the mitochondrion (reviewed, from a variety of perspectives, in Embley and Martin 2006; Martin et al. 2015; Eme et al. 2017; Roger et al. 2017). Genomic and phylogenetic evidence indicates that the host lineage belonged to the Asgardarchaeota superphylum (Spang et al. 2015; Zaremba-Niedzwiedzka et al. 2017). The origin of bacterial-type membrane phospholipids in eukaryotes is therefore an important evolutionary question that has received considerable attention (Woese et al. 1990; Kandler 1995; López-García and Moreira 2006; Baum and Baum 2014; Gould et al. 2016). Given the evidence for transfer of bacterial-type phospholipid biosynthesis genes into Archaea, one possibility—also raised by the results of Villanueva et al. (2017)—is that eukaryotes may have inherited their bacterial lipids vertically from the archaeal host cell. Both our study and that of Villanueva et al. (2017) point to the presence of orthologs for bacterial lipid genes in Asgardarchaeota. These include GlpA/GlpD, PlsC, and PlsY orthologs in *Lokiarchaeum* sp. GC14_75, PlsC, and PlsY in Heimdallarchaeota archaeon LC_2, and PlsY in Thorarchaeota archaeon SMTZ1-83 (table 1). However, phylogenies of these genes (supplementary figs. 13–15, Supplementary Material online) do not support a specific relationship between eukaryotes and any of the archaeal sequences, and so do not provide any compelling support for an origin of eukaryotic lipids via the archaeal host cell.

## Conclusions

Our phylogenetic analyses of lipid biosynthesis genes support two main conclusions about prokaryotic cell physiology and early cell evolution. First, our results corroborate previous evidence for extensive horizontal transfer of lipid genes, particularly from Archaea to Bacteria, from potentially very early to more recent evolutionary times. The functions of these genes remain unclear, but in *B. subtilis* (Guldan et al. 2008, 2011) they are involved in making archaeal-type G1P ether-linked lipids, whereas in the FCB lineage Cloacimonetes (Villanueva et al. 2018) they may be involved in synthesizing archaeal-type phospholipids that are incorporated into the bacterial cell membrane. Evidence that these genes have undergone horizontal transfer, both early in evolution and more recently, provides a potential mechanism for the remarkable diversity of membrane lipids, and especially ether lipids, in environmental settings (Schouten et al. 2001). We also note that it is intriguing that bacterial lipids with archaeal features are particularly abundant in settings characterized by high archaeal abundances, including cold seeps, wetlands and geothermal settings (Schouten et al. 2013), potentially providing ecological opportunity for gene transfer. Experimental work to characterize the enzymes that make these environmental lipids will be needed to test this prediction.

A second, and more tentative, result of our study relates to the antiquity of the canonical archaeal and bacterial pathways. Our analyses suggest that the enzymes for making G1P lipids may have been present in the common ancestor of Archaea and Bacteria. Under the consensus view that the root of the tree of life lies between Bacteria and Archaea, this would imply that LUCA could have made archaeal-type membranes. This finding is intriguing in light of previous work suggesting the presence of isoprenoids produced by the mevalonate pathway in LUCA (Lombard and Moreira 2011; Castelle and Banfield 2018). By contrast, we found no positive evidence to suggest that the bacterial pathway was present in LUCA, although our gene trees are poorly resolved and so we cannot exclude this possibility. The consensus universal root between Bacteria and Archaea is supported by analyses of ancient gene duplications (Gogarten et al. 1989; Iwabe et al. 1989; Zhaxybayeva et al. 2005) and genome networks (Dagan et al. 2010), but some analyses have supported an alternative placement of the root within Bacteria (Cavalier-Smith 2006; Lake et al. 2009; Williams et al. 2015). Our trees do not exclude a within-Bacteria root, in which case LUCA would have possessed the bacterial pathway, and the archaeal pathway would have evolved along the archaeal stem, or in a common ancestor of Archaea and Firmicutes (Cavalier-Smith 2006; Lake et al. 2009).

If one membrane lipid pathway evolved before the other, this would imply that one of the two prokaryotic lineages changed its membrane lipid composition during early evolution. The evolutionary processes that drive such changes remain unclear, in part because we still do not fully understand the functional differences between modern archaeal and bacterial membranes. Compared with bacterial-type membranes, archaeal-type membranes maintain their physiochemical properties over a broader range of temperatures and may be more robust to other environmental extremes (van de Vossenberg et al. 1998; Koga 2012). If the archaeal pathway is older than the bacterial pathway, then that could reflect a LUCA adapted to such extreme settings. It is then intriguing to speculate on the evolutionary drivers for subsequent adoption of bacterial-type membranes, especially because the Bacteria appear to be more successful than the Archaea in terms of abundance and genetic diversity (Danovaro et al. 2016; Hug et al. 2016; Castelle and Banfield 2018). Moreover, an analogous change has happened at least once in evolutionary history, during the origin of eukaryotic cells (Martin et al. 2015). Chemical considerations suggest such bonds ought to be energetically cheaper to make and break, although we know of no published experimental data on these relative biosynthetic costs. Alternatively, bacterial-type membrane lipids comprise a variety of fatty acyl moieties, varying in chain length, unsaturation, degree of branching and cyclisation, and these could impart a degree of flexibility and adaptability that provides a marginal benefit in dynamic mesophilic environments. If so, that advantage could translate to bacterial ether lipids that are also widespread in nonextreme settings and also characterized by a variety of alkyl forms (Pancost et al. 2001). Conversely, if bacterial-type membranes were ancestral, the transition to archaeal-type membranes could have been driven by adaptation to high environmental temperatures: ether bonds are more thermostable than esters (van de Vossenberg et al. 1998; Koga 2012) and are also found in the membranes of thermophilic Bacteria (Kaur et al. 2015). In any case, the widespread occurrence of bacterial-type, archaeal-type, and mixed-type membrane lipids in a range of environments, as well as the widespread occurrence of the associated biosynthetic genes across both domains, suggests that except for high temperature and low pH settings, the advantages of either membrane type is marginal.

## Supplementary Material

Supplementary data are available at *Genome Biology and Evolution* online.

## Supplementary Material

Supplementary Data

## References

[evz034-B1] Baum DA, Baum B. 2014 An inside-out origin for the eukaryotic cell. BMC Biol. 12:1–22. 25350791 10.1186/s12915-014-0076-2 PMC4210606

[evz034-B2] Bell SD, Jackson SP. 1998 Transcription and translation in Archaea: a mosaic of eukaryal and bacterial features. Trends Microbiol. 6(6):222–227. 9675798 10.1016/s0966-842x(98)01281-5

[evz034-B3] Boucher Y, Kamekura M, Doolittle WF. 2004 Origins and evolution of isoprenoid lipid biosynthesis in archaea. Mol Microbiol. 52(2):515–527. 15066037 10.1111/j.1365-2958.2004.03992.x

[evz034-B4] Caforio A, Siliakus MF, Exterkate M, Jain S, Jumde VR. 2018 Converting *Escherichia coli* into an archaebacterium with a hybrid heterochiral membrane. Proc Natl Acad Sci U S A. 115:1–6. 10.1073/pnas.1721604115 PMC5889666 29555770

[evz034-B5] Calvignac-Spencer S, Schulze JM, Zickmann F, Renard BY. 2014 Clock rooting further demonstrates that guinea 2014 EBOV is a member of the Zaïre lineage. PLoS Curr. 6: 1–8. 10.1371/currents.outbreaks.c0e035c86d721668a6ad7353f7f6fe86 PMC4073806 24987574

[evz034-B6] Carbone V, et al 2015 Structure and evolution of the archaeal lipid synthesis enzyme sn-glycerol-1-phosphate dehydrogenase. J Biol Chem. 290: 21690–21704. 26175150 10.1074/jbc.M115.647461 PMC4571891

[evz034-B7] Castelle CJ, Banfield JF. 2018 Major new microbial groups expand diversity and alter our understanding of the tree of life. Cell 172(6):1181–1197. 29522741 10.1016/j.cell.2018.02.016

[evz034-B8] Cavalier-Smith T. 2006 Rooting the tree of life by transition analyses. Biol Direct 1(1):19. 16834776 10.1186/1745-6150-1-19 PMC1586193

[evz034-B9] Criscuolo A, Gribaldo S. 2010 BMGE (Block Mapping and Gathering with Entropy): a new software for selection of phylogenetic informative regions from multiple sequence alignments. BMC Evol Biol. 10:210. 20626897 10.1186/1471-2148-10-210 PMC3017758

[evz034-B10] Dagan T, Roettger M, Bryant D, Martin W. 2010 Genome networks root the tree of life between prokaryotic domains. Genome Biol Evol. 2:379–392. 20624742 10.1093/gbe/evq025 PMC2997548

[evz034-B11] Danovaro R, Molari M, Corinaldesi C, Dell’Anno A. 2016 Macroecological drivers of archaea and bacteria in benthic deep-sea ecosystems. Sci Adv. 2:1–12. 10.1126/sciadv.1500961 PMC4928989 27386507

[evz034-B12] Drummond AJ, Rambaut A. 2007 BEAST: Bayesian evolutionary analysis by sampling trees. BMC Evol Biol. 7:1–8. 17996036 10.1186/1471-2148-7-214 PMC2247476

[evz034-B13] Drummond AJ, Suchard MA, Xie D, Rambaut A. 2012 Bayesian phylogenetics with BEAUti and the BEAST 1.7. Mol Biol Evol. 29(8):1969–1973. 22367748 10.1093/molbev/mss075 PMC3408070

[evz034-B14] Embley TM, Martin W. 2006 Eukaryotic evolution, changes and challenges. Nature 440(7084):623–630. 16572163 10.1038/nature04546

[evz034-B15] Eme L, Spang A, Lombard J, Stairs CW, Ettema TJG. 2017 Archaea and the origin of eukaryotes. Nat Rev Microbiol. 15(12):711–723. 29123225 10.1038/nrmicro.2017.133

[evz034-B16] Fan Q, Relini A, Cassinadri D, Gambacorta A, Gliozzi A. 1995 Stability against temperature and external agents of vesicles composed of archaeal bolaform lipids and egg PC. Biochim Biophys Acta Biomembr. 1240(1):83–88. 10.1016/0005-2736(95)00157-x 7495852

[evz034-B17] Garcia-Vallvé S, Romeu A, Palau J. 2000 Horizontal gene transfer in bacterial and archaeal complete genomes. Genome Res. 10(11):1719–1725. 11076857 10.1101/gr.130000 PMC310969

[evz034-B18] Gattinger A, Schloter M, Munch JC. 2002 Phospholipid etherlipid and phospholipid fatty acid fingerprints in selected euryarchaeotal monocultures for taxonomic profiling. FEMS Microbiol Lett. 213(1):133–139. 12127500 10.1111/j.1574-6968.2002.tb11297.x

[evz034-B19] Gogarten JP, Rausch T, Bernasconi P, Kibak H, Taiz L. 1989 Molecular evolution of H^+^-ATPases. I. Methanococcus and sulfolobus are monophyletic with respect to eukaryotes and eubacteria. Zeitschrift fur Naturforsch Sect C J Biosci. 44(7–8):641–650. 10.1515/znc-1989-7-816 2528356

[evz034-B20] Goldfine H. 2010 The appearance, disappearance and reappearance of plasmalogens in evolution. Prog Lipid Res. 49(4):493–498. 20637230 10.1016/j.plipres.2010.07.003

[evz034-B21] Gould SB, Garg SG, Martin WF. 2016 Bacterial vesicle secretion and the evolutionary origin of the eukaryotic endomembrane system. Trends Microbiol. 24(7):525–534. 27040918 10.1016/j.tim.2016.03.005

[evz034-B22] Gouy R, Baurain D, Philippe H. 2015 Rooting the tree of life: the phylogenetic jury is still out. Philos Trans R Soc B Biol Sci. 370(1678):20140329. 10.1098/rstb.2014.0329 PMC4571568 26323760

[evz034-B23] Guldan H, Matysik FM, Bocola M, Sterner R, Babinger P. 2011 Functional assignment of an enzyme that catalyzes the synthesis of an archaea-type ether lipid in bacteria. Angew Chemie Int Ed. 50(35):8188–8191. 10.1002/anie.201101832 21761520

[evz034-B24] Guldan H, Sterner R, Babinger P. 2008 Identification and characterization of a bacterial glycerol-1-phosphate dehydrogenase: Ni^2+^-dependent AraM from *Bacillus subtilis*. Biochemistry 47(28):7376–7384. 18558723 10.1021/bi8005779

[evz034-B25] Hartmann K, Wong D, Stadler T. 2010 Sampling trees from evolutionary models. Syst Biol. 59(4):465–476. 20547782 10.1093/sysbio/syq026

[evz034-B26] Hemmi H, Shibuya K, Takahashi Y, Nakayama T, Nishino T. 2004 (*S*)-2,3-Di-*O*-geranylgeranylglyceryl phosphate synthase from the thermoacidophilic archaeon *Sulfolobus solfataricus*: molecular cloning and characterization of a membrane-intrinsic prenyltransferase involved in the biosynthesis of archaeal ether-linked membrane lipids. J Biol Chem. 279(48):50197–50203. 15356000 10.1074/jbc.M409207200

[evz034-B27] Hug LA, et al 2016 A new view of the tree of life. Nautre 1:16048. 10.1038/nmicrobiol.2016.48 27572647

[evz034-B28] Iwabe N, Kuma KI, Hasegawa M, Osawa S, Miyata T. 1989 Evolutionary relationship of archaebacteria, eubacteria, and eukaryotes inferred from phylogenetic trees of duplicated genes. Proc Natl Acad Sci U S A. 86(23):9355–9359. 2531898 10.1073/pnas.86.23.9355 PMC298494

[evz034-B29] Kandler O. 1995 Cell wall biochemistry in Archaea and its phylogenetic implications. J Biol Phys. 20(1–4):165–169.

[evz034-B30] Kates M. 1977 The phytanyl ether-linked polar lipids and isoprenoid neutral lipids of extremely halophilic bacteria. Prog Chem. 15(4):301–342. 10.1016/0079-6832(77)90011-8 358256

[evz034-B31] Katoh K, Misawa K, Kuma K, Miyata T. 2002 MAFFT: a novel method for rapid multiple sequence alignment based on fast Fourier transform. Nucleic Acids Res. 30(14):3059–3066. 12136088 10.1093/nar/gkf436 PMC135756

[evz034-B32] Kaur G, Mountain BW, Stott MB, Hopmans EC, Pancost RD. 2015 Temperature and pH control on lipid composition of silica sinters from diverse hot springs in the Taupo Volcanic Zone, New Zealand. Extremophiles 19(2):327–344. 25515367 10.1007/s00792-014-0719-9 PMC4339782

[evz034-B33] Kelman LM, Kelman Z. 2014 Archaeal DNA replication. Annu Rev Genet. 48(1):71–97. 25421597 10.1146/annurev-genet-120213-092148

[evz034-B34] Koga Y. 2011 Early evolution of membrane lipids: how did the lipid divide occur? J Mol Evol. 72(3):274–282. 21259003 10.1007/s00239-011-9428-5

[evz034-B35] Koga Y. 2012 Thermal adaptation of the archaeal and bacterial lipid membranes. Archaea 2012:1. 10.1155/2012/789652 PMC3426160 22927779

[evz034-B36] Koga Y. 2014 From promiscuity to the lipid divide: on the evolution of distinct membranes in archaea and bacteria. J Mol Evol. 78(3–4):234–242. 24573438 10.1007/s00239-014-9613-4

[evz034-B37] Lake JA, Skophammer RG, Herbold CW, Servin JA. 2009 Genome beginnings: rooting the tree of life. Philos Trans R Soc Lond B Biol Sci. 364(1527):2177–2185. 19571238 10.1098/rstb.2009.0035 PMC2873003

[evz034-B38] Landan G, Graur D. 2007 Heads or tails: a simple reliability check for multiple sequence alignments. Mol Biol Evol. 24(6):1380–1383. 17387100 10.1093/molbev/msm060

[evz034-B39] Lartillot N, Brinkmann H, Philippe H. 2007 Suppression of long-branch attraction artefacts in the animal phylogeny using a site-heterogeneous model. BMC Evol Biol. 7:1–14. 17288577 10.1186/1471-2148-7-S1-S4 PMC1796613

[evz034-B40] Lartillot N, Philippe H. 2004 A Bayesian mixture model for across-site heterogeneities in the amino-acid replacement process. Mol Biol Evol. 21(6):1095–1109. 15014145 10.1093/molbev/msh112

[evz034-B41] Le SQ, Gascuel O. 2008 An improved general amino acid replacement matrix. Mol Biol Evol. 25(7):1307–1320. 18367465 10.1093/molbev/msn067

[evz034-B42] Le SQ, Lartillot N, Gascuel O. 2008 Phylogenetic mixture models for proteins. Philos Trans R Soc B Biol Sci. 363(1512):3965–3976. 10.1098/rstb.2008.0180 PMC2607422 18852096

[evz034-B43] Lombard J, López-García P, Moreira D. 2012a The early evolution of lipid membranes and the three domains of life. Nat Rev Microbiol. 10(7):507–515. 22683881 10.1038/nrmicro2815

[evz034-B44] Lombard J, López-García P, Moreira D. 2012b Phylogenomic investigation of phospholipid synthesis in archaea. Archaea 2012:630910. 23304072 10.1155/2012/630910 PMC3533463

[evz034-B45] Lombard J, Moreira D. 2011 Origins and early evolution of the mevalonate pathway of isoprenoid biosynthesis in the three domains of life. Mol Biol Evol. 28(1):87–99. 20651049 10.1093/molbev/msq177

[evz034-B46] López-García P, Moreira D. 2006 Selective forces for the origin of the eukaryotic nucleus. BioEssays 28(5):525–533. 16615090 10.1002/bies.20413

[evz034-B47] Martin WF, Garg S, Zimorski V. 2015 Endosymbiotic theories for eukaryote origin. Philos Trans R Soc B Biol Sci. 370(1678):20140330. 10.1098/rstb.2014.0330 PMC4571569 26323761

[evz034-B48] Nemoto N, Oshima T, Yamagishi A. 2003 Purification and characterization of geranylgeranylglyceryl phosphate synthase from a thermoacidophilic archaeon, *Thermoplasma acidophilum*. J Biochem. 133(5):651–657. 12801917 10.1093/jb/mvg083

[evz034-B49] Nguyen LT, Schmidt HA, Von Haeseler A, Minh BQ. 2015 IQ-TREE: a fast and effective stochastic algorithm for estimating maximum-likelihood phylogenies. Mol Biol Evol. 32(1):268–274. 25371430 10.1093/molbev/msu300 PMC4271533

[evz034-B50] Nishihara M, Yamazaki T, Oshima T, Koga Y. 1999 *sn*-Glycerol-1-phosphate-forming activities in Archaea: separation of archaeal phospholipid biosynthesis and glycerol catabolism by glycerophosphate enantiomers. J Bacteriol. 181(4):1330–1333. 9973362 10.1128/jb.181.4.1330-1333.1999 PMC93513

[evz034-B51] Pancost RD, Bouloubassi I, Aloisi G, Sinninghe Damsté JS; the Medinaut ShipboardScientific Party. 2001 Three series of non-isoprenoidal dialkyl glycerol diethers in cold-seep carbonate crusts. Org Geochem. 32(5):695–707.

[evz034-B52] Parks DH, Imelfort M, Skennerton CT, Hugenholtz P, Tyson GW. 2015 CheckM: assessing the quality of microbial genomes recovered from isolates, single cells, and metagenomes. Genome Res. 25(7):1043–1055. 25977477 10.1101/gr.186072.114 PMC4484387

[evz034-B53] Parsons JB, Rock CO. 2013 Bacterial lipids: metabolism and membrane homeostasis. Prog Lipid Res. 52(3):249–276. 23500459 10.1016/j.plipres.2013.02.002 PMC3665635

[evz034-B54] Payandeh J, Fujihashi M, Gillon W, Pai EF. 2006 The crystal structure of (*S*)-3-*O*-geranylgeranylglyceryl phosphate synthase reveals an ancient fold for an ancient enzyme. J Biol Chem. 281(9):6070–6078. 16377641 10.1074/jbc.M509377200

[evz034-B55] Penny D. 1976 Criteria for optimising phylogenetic trees and the problem of determining the root of a tree. J Mol Evol. 8(2):95–116. 966292 10.1007/BF01739097

[evz034-B56] Peretó J, López-García P, Moreira D. 2004 Ancestral lipid biosynthesis and early membrane evolution. Trends Biochem Sci. 29(9):469–477. 15337120 10.1016/j.tibs.2004.07.002

[evz034-B57] Peterhoff D, et al 2014 A comprehensive analysis of the geranylgeranylglyceryl phosphate synthase enzyme family identifies novel members and reveals mechanisms of substrate specificity and quaternary structure organization. Mol Microbiol. 92(4):885–899. 24684232 10.1111/mmi.12596

[evz034-B58] Reeve JN, Sandman K, Daniels CJ. 1997 Archaeal histones, nucleosomes, and transcription initiation. Cell 89(7):999–1002. 9215621 10.1016/s0092-8674(00)80286-x

[evz034-B59] Roger AJ, Muñoz-Gómez SA, Kamikawa R. 2017 The origin and diversification of mitochondria. Curr Biol. 27(21):R1177–R1192. 29112874 10.1016/j.cub.2017.09.015

[evz034-B60] Schouten S, Hopmans EC, Pancost RD, Sinninghe DJS. 2000 Widespread occurrence of structurally diverse tetraether membrane lipids: evidence for the ubiquitous presence of low-temperature relatives of hyperthermophiles. Proc Natl Acad Sci U S A. 97(26):14421–14426. 11121044 10.1073/pnas.97.26.14421 PMC18934

[evz034-B61] Schouten S, Hopmans EC, Sinninghe Damsté JS. 2013 The organic geochemistry of glycerol dialkyl glycerol tetraether lipids: a review. Org Geochem. 54:19–61.

[evz034-B62] Schouten S, Wakeham SG, Sinninghe Damsté JS. 2001 Evidence for anaerobic methane oxidation by archea in euxinic waters of the Black Sea. Org Geochem. 32(10):1277–1281.

[evz034-B63] Shimada H, Yamagishi A. 2011 Stability of heterochiral hybrid membrane made of bacterial *sn*-G3P lipids and archaeal *sn*-G1P lipids. Biochemistry 50(19):4114–4120. 21473653 10.1021/bi200172d

[evz034-B64] Sinninghe Damsté JS, et al 2002 Linearly concatenated cyclobutane lipids form a dense bacterial membrane. Nature 419(6908):708–712. 12384695 10.1038/nature01128

[evz034-B65] Sinninghe Damsté JS, et al 2007 Structural characterization of diabolic acid-based tetraester, tetraether and mixed ether/ester, membrane-spanning lipids of bacteria from the order Thermotogales. Arch Microbiol. 188(6):629–641. 17643227 10.1007/s00203-007-0284-z PMC2111041

[evz034-B66] Sojo V, Pomiankowski A, Lane N. 2014 A bioenergetic basis for membrane divergence in archaea and bacteria. PLoS Biol. 12:e1001926. 25116890 10.1371/journal.pbio.1001926 PMC4130499

[evz034-B67] Spang A, et al 2015 Complex archaea that bridge the gap between prokaryotes and eukaryotes. Nature 521(7551):173–179. 25945739 10.1038/nature14447 PMC4444528

[evz034-B68] Stadler T. 2009 On incomplete sampling under birth-death models and connections to the sampling-based coalescent. J Theor Biol. 261(1):58–66. 19631666 10.1016/j.jtbi.2009.07.018

[evz034-B69] Tria FDK, Landan G, Dagan T. 2017 Phylogenetic rooting using minimal ancestor deviation. Nat Ecol Evol. 1:193. 29388565 10.1038/s41559-017-0193

[evz034-B70] van de Vossenberg JLCM, Driessen AJM, Konings WN. 1998 The essence of being extremophilic: the role of the unique archaeal membrane lipids. Extremophiles 2(3):163–170. 9783161 10.1007/s007920050056

[evz034-B71] Villanueva L, Schouten S, Sinninghe Damsté JS. 2017 Phylogenomic analysis of lipid biosynthetic genes of Archaea shed light on the ‘lipid divide’. Environ Microbiol. 19(1):54–69. 27112361 10.1111/1462-2920.13361

[evz034-B72] Villanueva L, et al 2018 Bridging the divide: bacteria synthesizing archaeal membrane lipids. bioRxiv 448035.

[evz034-B73] Wächtershäuser G. 2003 From pre-cells to Eukarya—a tale of two lipids. Mol Microbiol. 47(1):13–22. 12492850 10.1046/j.1365-2958.2003.03267.x

[evz034-B74] Weijers JWH, et al 2006 Membrane lipids of mesophilic anaerobic bacteria thriving in peats have typical archaeal traits. Environ Microbiol. 8(4):648–657. 16584476 10.1111/j.1462-2920.2005.00941.x

[evz034-B75] Weiss MC, et al 2016 The physiology and habitat of the last universal common ancestor. Nat Microbiol 1:1–8. 10.1038/nmicrobiol.2016.116 27562259

[evz034-B76] Wilkinson M, McInerney JO, Hirt RP, Foster PG, Embley TM. 2007 Of clades and clans: terms for phylogenetic relationships in unrooted trees. Trends Ecol Evol. 22(3):114–115. 17239486 10.1016/j.tree.2007.01.002

[evz034-B77] Williams TA, Embley TM, Heinz E. 2011 Informational gene phylogenies do not support a fourth domain of life for nucleocytoplasmic large DNA viruses. PLoS One 6(6):e21080. 21698163 10.1371/journal.pone.0021080 PMC3116878

[evz034-B78] Williams TA, Foster PG, Cox CJ, Embley TM. 2013 An archaeal origin of eukaryotes supports only two primary domains of life. Nature 504(7479):231–236. 24336283 10.1038/nature12779

[evz034-B79] Williams TA, et al 2015 New substitution models for rooting phylogenetic trees. Philos Trans R Soc B Biol Sci. 370(1678):20140336. 10.1098/rstb.2014.0336 PMC4571574 26323766

[evz034-B80] Williams TA, et al 2017 Integrative modeling of gene and genome evolution roots the archaeal tree of life. Proc Natl Acad Sci U S A. 114(23):E4602–E4611. 28533395 10.1073/pnas.1618463114 PMC5468678

[evz034-B81] Woese CR, Kandler O, Wheelis ML. 1990 Towards a natural system of organisms: proposal for the domains Archaea, Bacteria, and Eucarya. Proc Natl Acad Sci U S A. 87(12):4576–4579. 2112744 10.1073/pnas.87.12.4576 PMC54159

[evz034-B82] Yang Z. 1994 Maximum likelihood phylogenetic estimation from DNA sequences with variable rates over sites: Approximate methods. J Mol Evol. 39(3):306–314. 7932792 10.1007/BF00160154

[evz034-B83] Yao J, Rock CO. 2013 Phosphatidic acid synthesis in bacteria. Biochim Biophys Acta 1831(3):495–502. 22981714 10.1016/j.bbalip.2012.08.018 PMC3548993

[evz034-B84] Yokobori S, Nakajima Y, Akanuma S, Yamagishi A. 2016 Birth of archaeal cells: molecular phylogenetic analyses of G1P dehydrogenase, G3P dehydrogenases, and glycerol kinase suggest derived features of archaeal membranes having G1P polar lipids. Archaea 2016:1–16. 10.1155/2016/1802675 PMC5059525 27774041

[evz034-B85] Zaremba-Niedzwiedzka K, et al 2017 Asgard archaea illuminate the origin of eukaryotic cellular complexity. Nature 541(7637):353–358. 28077874 10.1038/nature21031

[evz034-B86] Zhaxybayeva O, Lapierre P, Gogarten JP. 2005 Ancient gene duplications and the root(s) of the tree of life. Protoplasma 227(1):53–64. 16389494 10.1007/s00709-005-0135-1

