# Supplementary material for: Investigating the Origins of Membrane Phospholipid Biosynthesis Genes Using Outgroup-Free Rooting
Source: Genome Biol Evol. 2019 Feb 8;11(3):883–98. doi: 10.1093/gbe/evz034 (PMC6431249; doi:10.1093/gbe/evz034)
Supplement: Supplementary Data [file evz034_supp.zip › legends_for_supplementary_figures_and_tables.rtf]

Supplementary FiguresSupplementary Figure 1. G1PDH full tree, 111 sequences, 190 positions, inferred under LG+C60 model. Root position inferred using relaxed uncorrelated lognormal clock model with a Yule prior and LG substitution model. MAD root indicated with asterisk.  Supplementary Figure 2. GGGPS full tree, 133 sequences, 129 positions, inferred under LG+C40 model. Root position inferred using relaxed uncorrelated lognormal clock model with a Yule prior and LG substitution model. MAD root indicated with asterisk.   Supplementary Figure 3. GGGPS large subclade, 98 sequences, 166 positions, inferred under LG+C40 model. Root position inferred using relaxed uncorrelated lognormal clock model with a Yule prior and LG substitution model. MAD root indicated with asterisk.   Supplementary Figure 4. DGGGPS full tree, 97 sequences, 119 positions, inferred under LG+C60 model. Root position inferred using relaxed uncorrelated lognormal clock model with a Yule prior and LG substitution model. MAD root indicated with asterisk.   Supplementary Figure 5. GpsA full tree, 84 sequences, 169 positions, inferred under LG+C60 model. Root position inferred using relaxed uncorrelated lognormal clock model with a Yule prior and LG substitution model. MAD root indicated with asterisk.   Supplementary Figure 6. Glp full tree, 51 sequences, 199 positions, inferred under LG+C40 model. Root position inferred using relaxed uncorrelated lognormal clock model with a Yule prior and LG substitution model. MAD root indicated with asterisk.   Supplementary Figure 7. GlpK full tree, 77 sequences, 363 positions, inferred under LG+C60 model. Root position inferred using relaxed uncorrelated lognormal clock model with a Yule prior and LG substitution model. MAD root indicated with asterisk.   Supplementary Figure 8. PlsC full tree, 74 sequences, 57 positions, inferred under LG+C60 model. Root position inferred using relaxed uncorrelated lognormal clock model with a Yule prior and LG substitution model. MAD root between clade comprising of sequences in red and other sequences.   Supplementary Figure 9. PlsY full tree, 60 sequences, 104 positions, inferred under LG+C50 model. Root position inferred using relaxed uncorrelated lognormal clock model with a Yule prior and LG substitution model. MAD root indicated with asterisk.   Supplementary Figure 10. G1PDH full tree, 123 sequences, 173 positions, inferred under LG+C60 model. Root position inferred using  3-dehydroquinate synthase (DHQS), five glucerol dehydrogenase (GDH) and five alcohol dehydrogenase (ALDH) sequences as an outgroup  Supplementary Figure 11. Glp full tree, 63 sequences, 183 positions, inferred under LG+C60 model. Root position inferred using 12 FAD-dependent oxidoreductase sequences  as an outgroup Supplementary Figure 12. Gpsa full tree, 96 sequences, 148 positions, inferred under LG+C60 model. Root position inferred using six hydroxyacyl-CoA dehydrogenase (HACDH) and 6 UDP-glucose 6-dehydrogenase (UDPGDH) sequences  as an outgroup Supplementary Figure 13. Gpsa full tree with eukaryotic sequences, 113 sequences, 159 positions, inferred under LG+C50 model.Supplementary Figure 14. Glp full tree with eukaryotic sequences, 80 sequences, 190 positions, inferred under LG+C50 model.Supplementary Figure 15. PlsC full tree with eukaryotic sequences, 96 sequences, 54 positions, inferred under LG+C60 model.Supplementary Figure 16. Unrooted G1PDH full tree, 111 sequences, 190 positions, inferred under LG+C60 model. Supplementary Figure 17. Unrooted GGGPS full tree, 133 sequences, 129 positions, inferred under LG+C40 model. Supplementary Figure 18. Unrooted DGGGPS full tree, 97 sequences, 119 positions, inferred under LG+C60 model. Supplementary Figure 19. Unrooted GpsA full tree, 84 sequences, 169 positions, inferred under LG+C60 model.  Supplementary Figure 20. Unrooted Glp full tree, 51 sequences, 199 positions, inferred under LG+C40 model.  Supplementary Figure 21. Unrooted GlpK full tree, 77 sequences, 363 positions, inferred under LG+C60 model. Supplementary Figure 22. Unrooted PlsC full tree, 74 sequences, 57 positions, inferred under LG+C60 model.  Supplementary Figure 23. Unrooted PlsY full tree, 60 sequences, 104 positions, inferred under LG+C50 model Supplementary Figure 24. Gpsa full tree, 96 sequences, 148 positions, inferred under LG+C50 model. Root position inferred using six hydroxyacyl-CoA dehydrogenase (HACDH) and 6 UDP-glucose 6-dehydrogenase (UDPGDH) sequences  as an outgroup Supplementary Figure 25. UbiA full tree, 227 sequences, 69 positions, inferred under LG+C60 model. DGGGP sequences in blue, chlorophyll a synthase in green, protoheme IX farnesyltransferase in red, and 4-hydroxybenzoate octaprenyltransferase in black.  Supplementary Figure 26. Unrooted GGGPS full tree, 133 sequences, 129 positions, inferred under LG+C60 model.  Supplementary Figure 27. Unrooted PlsY full tree, 60 sequences, 104 positions, inferred under LG+C60 model Supplementary Figure 28. GlpA/GlpD full tree with eukaryotic sequences, 80 sequences, 190 positions, inferred under LG+C60 model Supplementary Figure 29. Gpsa full tree with eukaryotic sequences, 113 sequences, 159 positions, inferred under LG+C60 model Supplementary Figure 30. Maximum likelihood G1PDH tree inferred in IQ-Tree under the LG+C60 model. Rooted using MAD Supplementary Figure 31. Maximum likelihood GGGPS tree inferred in IQ-Tree under the LG+C60 model. Rooted using MADSupplementary Figure 32. Maximum likelihood DGGGPS tree inferred in IQ-Tree under the LG+C60 model. Rooted using MADSupplementary Figure 33. Maximum likelihood GpsA tree inferred in IQ-Tree under the LG+C60 model. Rooted using MADSupplementary Figure 34. Maximum likelihood GlpA/GlpD tree inferred in IQ-Tree under the LG+C60 model. Rooted using MAD Supplementary Figure 35. Maximum likelihood GlpK tree inferred in IQ-Tree under the LG+C60 model. Rooted using MADSupplementary Figure 36. Maximum likelihood PlsC tree inferred in IQ-Tree under the LG+C60 model. Rooted using MADSupplementary Figure 37. Maximum likelihood PlsY tree inferred in IQ-Tree under the LG+C60 model. Rooted using MADSupplementary Figure 38. Maximum likelihood G1PDH tree inferred in IQ-Tree under the LG+C60 model from HoT alignments. Rooted using MADSupplementary Figure 39. Maximum likelihood GGGPS tree inferred in IQ-Tree under the LG+C60 model from HoT alignments. Rooted using MADSupplementary Figure 40. Maximum likelihood DGGGPS tree inferred in IQ-Tree under the LG+C60 model from HoT alignments. Rooted using MADSupplementary Figure 41. Maximum likelihood GpsA tree inferred in IQ-Tree under the LG+C60 model from HoT alignments. Rooted using MADSupplementary Figure 42. Maximum likelihood GlpA/GlpD tree inferred in IQ-Tree under the LG+C60 model from HoT alignments. Rooted using MADSupplementary Figure 43. Maximum likelihood GlpK tree inferred in IQ-Tree under the LG+C60 model from HoT alignments. Rooted using MADSupplementary Figure 44. Maximum likelihood PlsC tree inferred in IQ-Tree under the LG+C60 model from HoT alignments. Rooted using MADSupplementary Figure 45. Maximum likelihood PlsY tree inferred in IQ-Tree under the LG+C60 model from HoT alignments. Rooted using MADSupplementary Figure 46. Maximum likelihood G1PDH tree inferred in IQ-Tree under the LG+C60 model from alignments with metagenomic data removed. Rooted using MAD, with lognormal relaxed molecular clock show with an asteriskSupplementary Figure 47. Maximum likelihood GGGPS tree inferred in IQ-Tree under the LG+C60 model from alignments with metagenomic data removed. Rooted using MAD, with lognormal relaxed molecular clock show with an asteriskSupplementary Figure 48. Maximum likelihood reduce GGGPS tree inferred in IQ-Tree under the LG+C60 model from alignments with metagenomic data removed. Rooted using MAD, with lognormal relaxed molecular clock show with an asterisk Supplementary Figure 49. Maximum likelihood DGGGPS tree inferred in IQ-Tree under the LG+C60 model from alignments with metagenomic data removed. Rooted using MAD, with lognormal relaxed molecular clock show with an asteriskSupplementary Figure 50.  Maximum likelihood GpsA tree inferred in IQ-Tree under the LG+C60 model from alignments with metagenomic data removed. Rooted using MAD, with lognormal relaxed molecular clock show with an asteriskSupplementary Figure 51.  Maximum likelihood GlpA/GlpD tree inferred in IQ-Tree under the LG+C60 model from alignments with metagenomic data removed. Rooted using MAD, with lognormal relaxed molecular clock show with an asteriskSupplementary Figure 52.  Maximum likelihood GlpK tree inferred in IQ-Tree under the LG+C60 model from alignments with metagenomic data removed. Rooted using MAD, with lognormal relaxed molecular clock show with an asteriskSupplementary Table 1. Highest marginal posterior probabilities for each gene tree for both the molecular clock and MAD rooting methods.Supplementary Table 2. Comparison of Bayesian Information Criterion (BIC) scores for the models selected by IQTree compared to the model used by Yokobori et al. (LG+gamma) for the outgrip rooting analyses. Lower BIC scores indicate better fit of model to data. Supplementary Table 3. Ambiguity Index (AI) scores for the MAD roots for each gene tree. Supplementary Table 4. MAD scores for the MAD roots for each gene tree.Supplementary Table 5. Accession numbers for sequences used in phylogenetic analyses. 
