# Supplementary material for: Investigating the Origins of Membrane Phospholipid Biosynthesis Genes Using Outgroup-Free Rooting
Source: Genome Biol Evol. 2019 Feb 8;11(3):883–98. doi: 10.1093/gbe/evz034 (PMC6431249; doi:10.1093/gbe/evz034)

Supplementary Figure 1

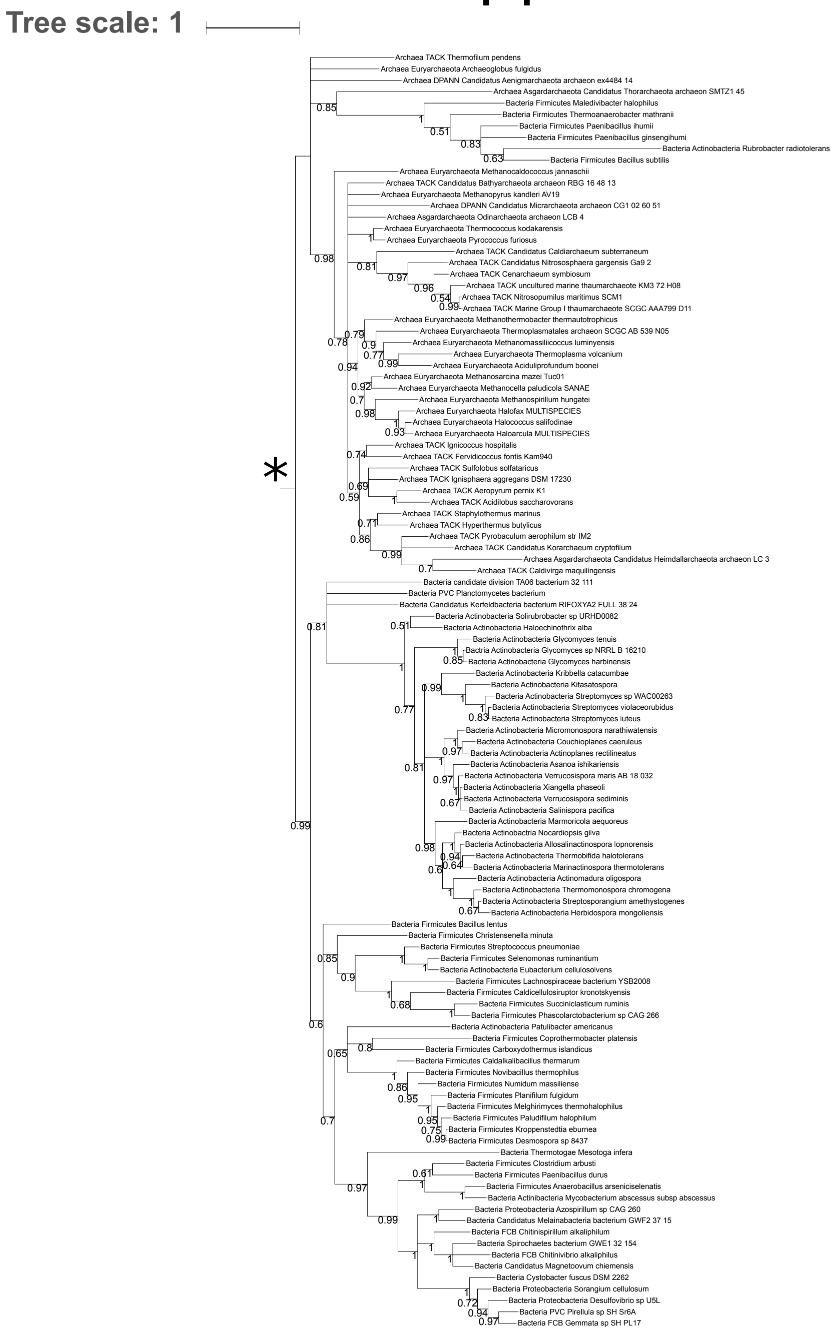

Supplementary Figure 2

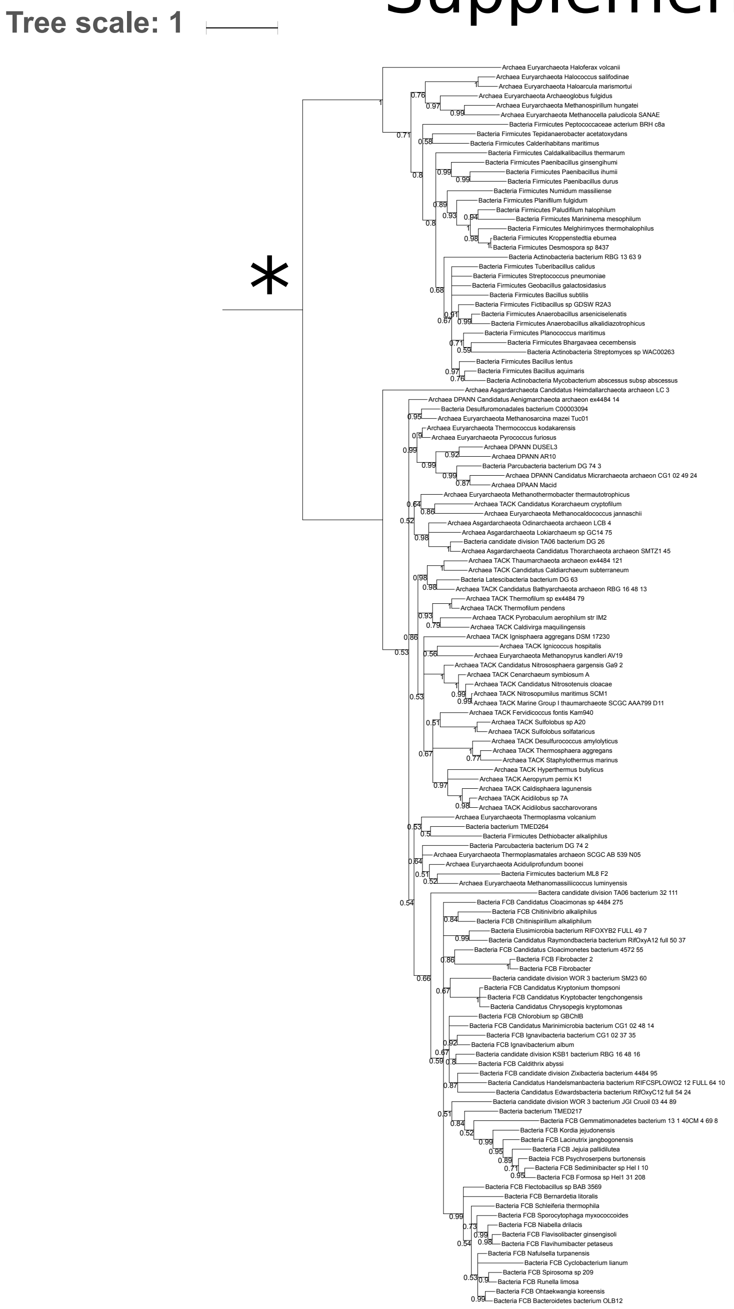

Supplementary Figure 3

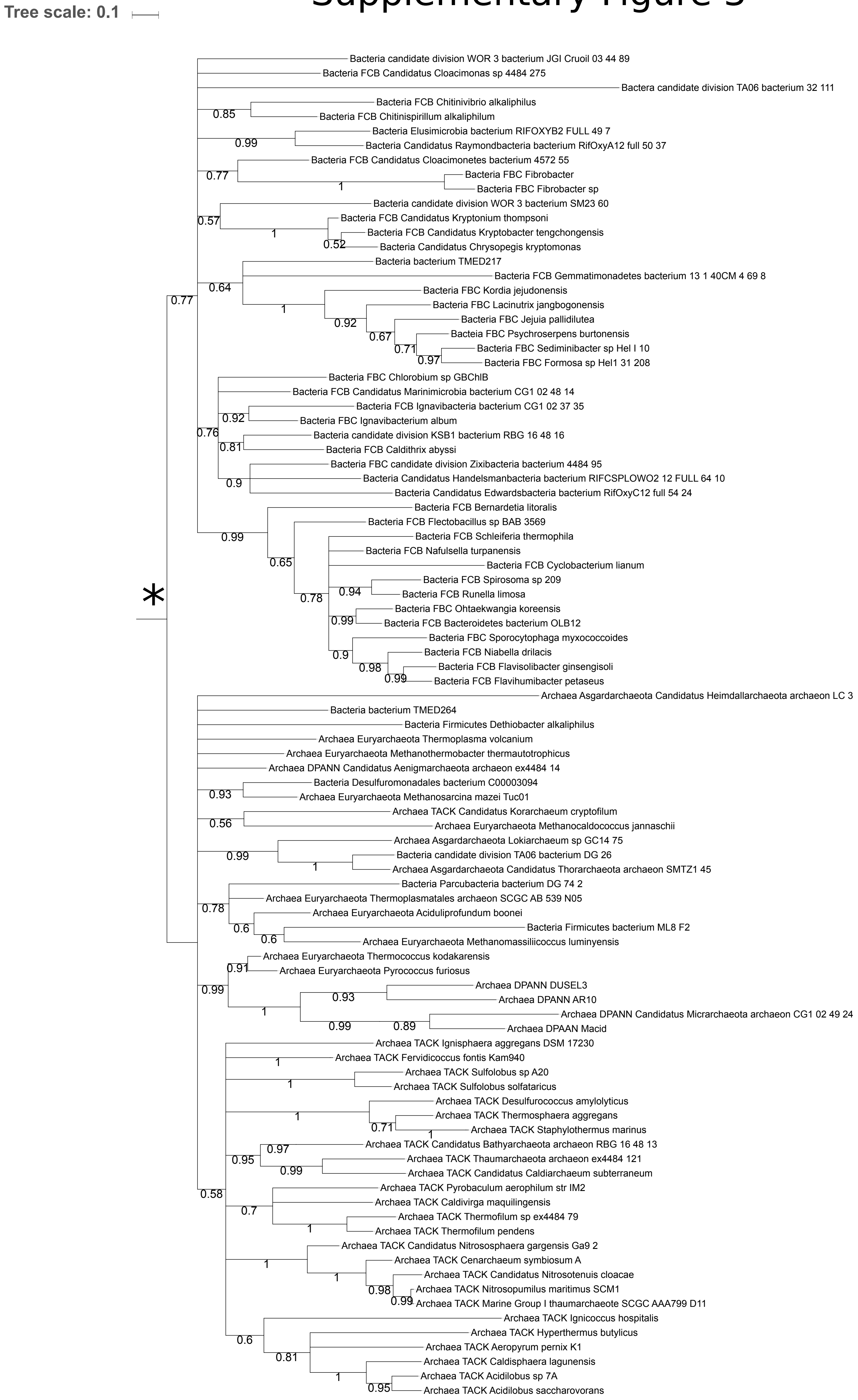

Supplementary Figure 4

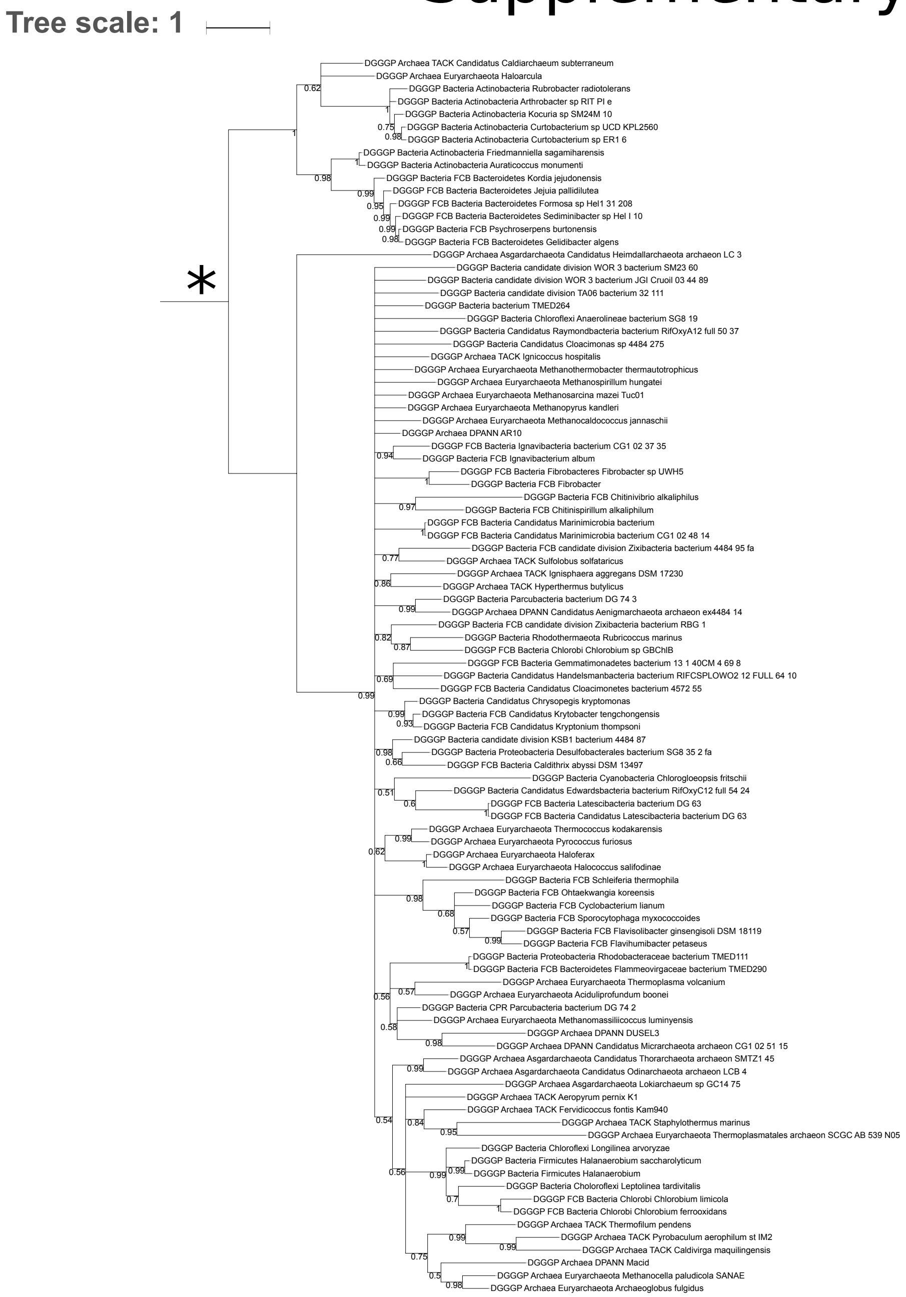

Supplementary Figure 5

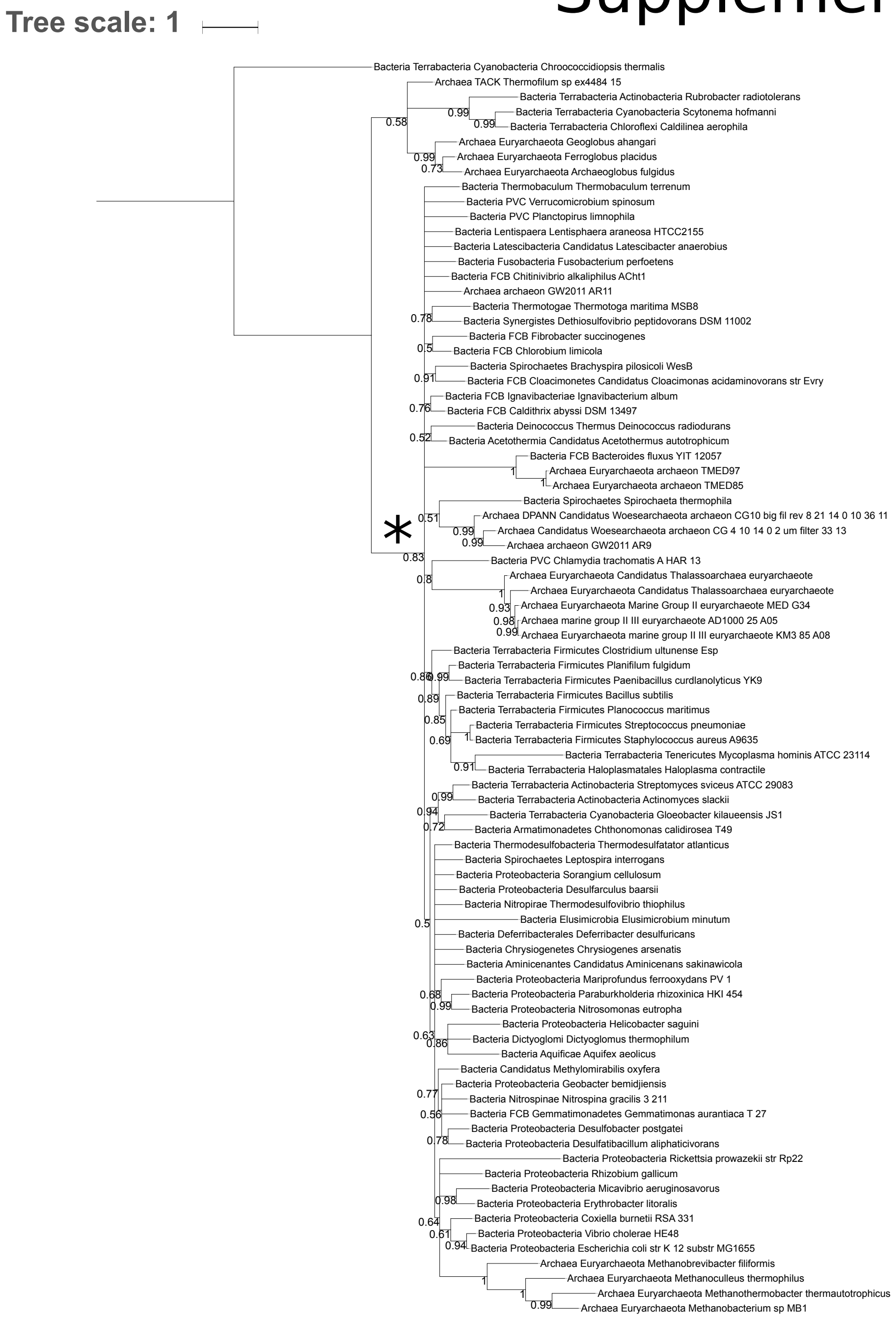

Supplementary Figure 6

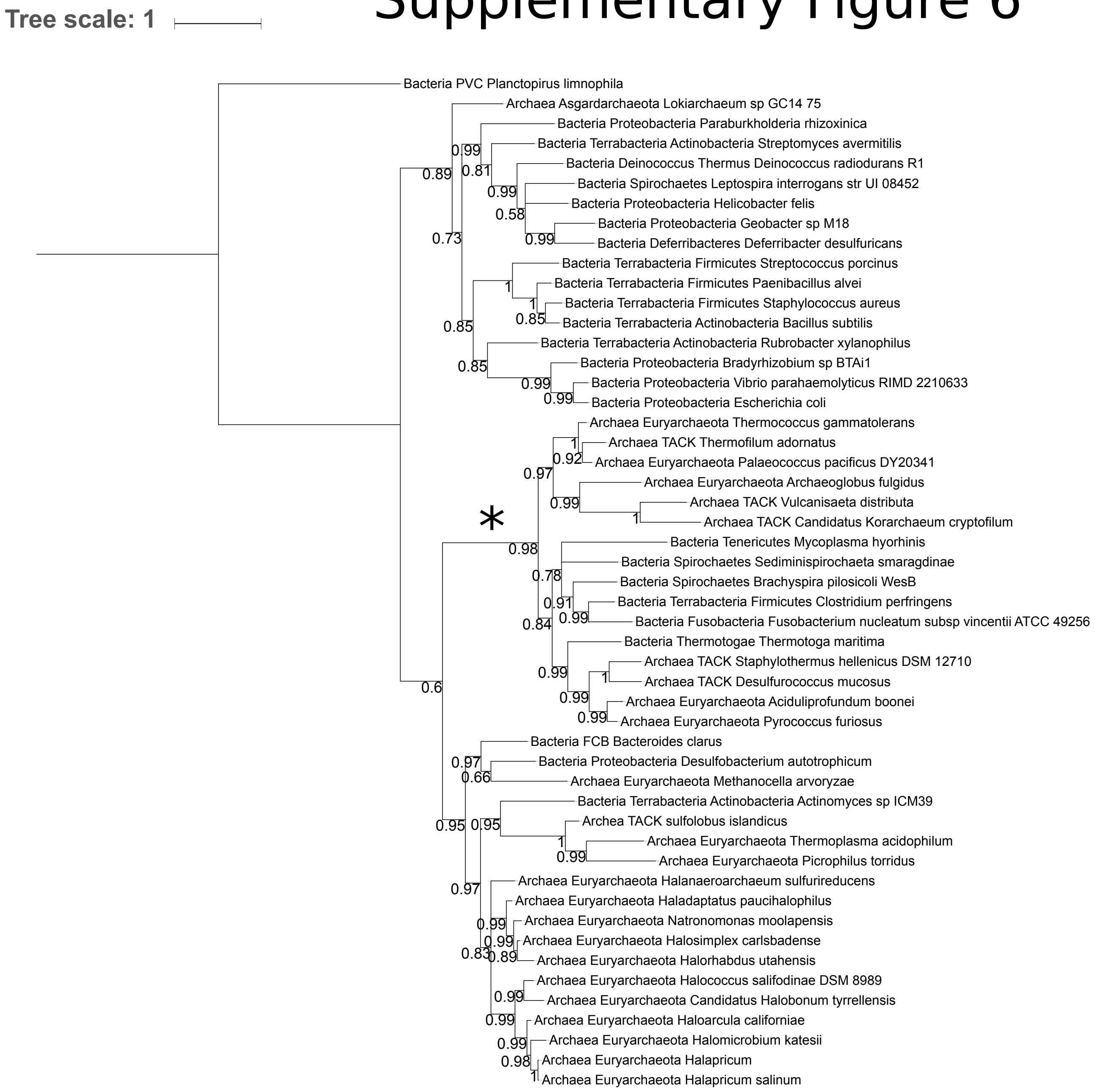

Supplementary Figure 7

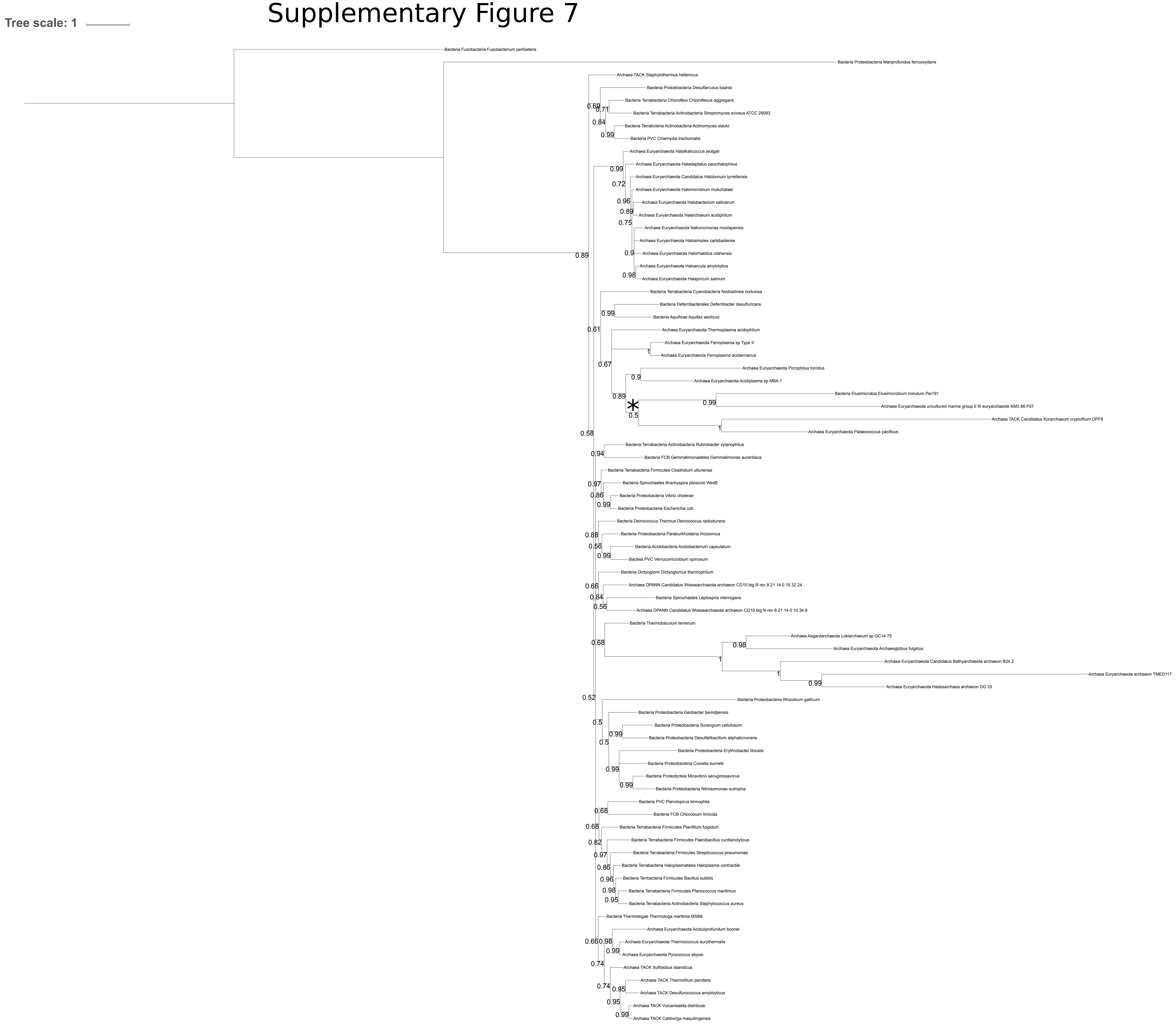

Supplementary Figure 8

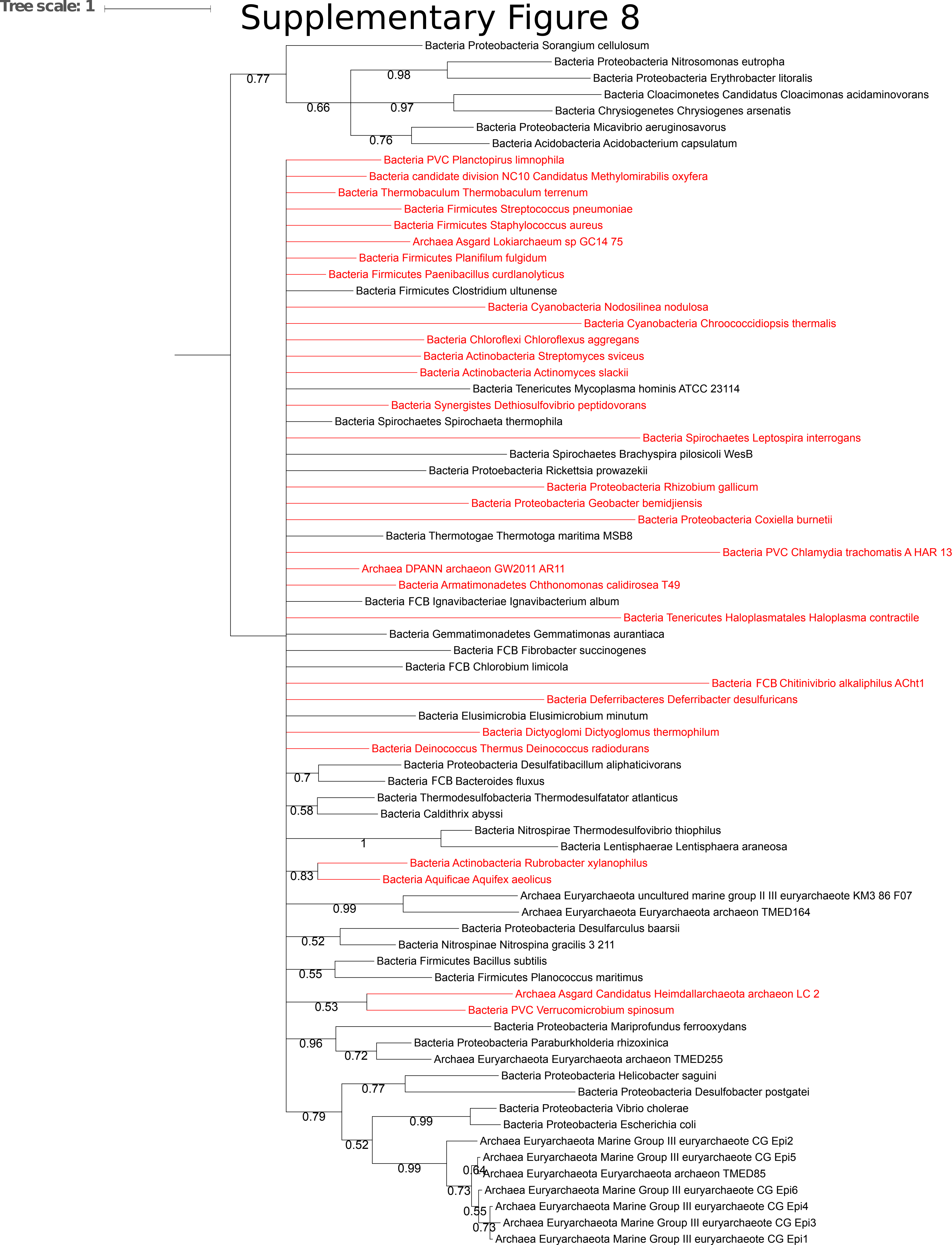

Supplementary Figure 9

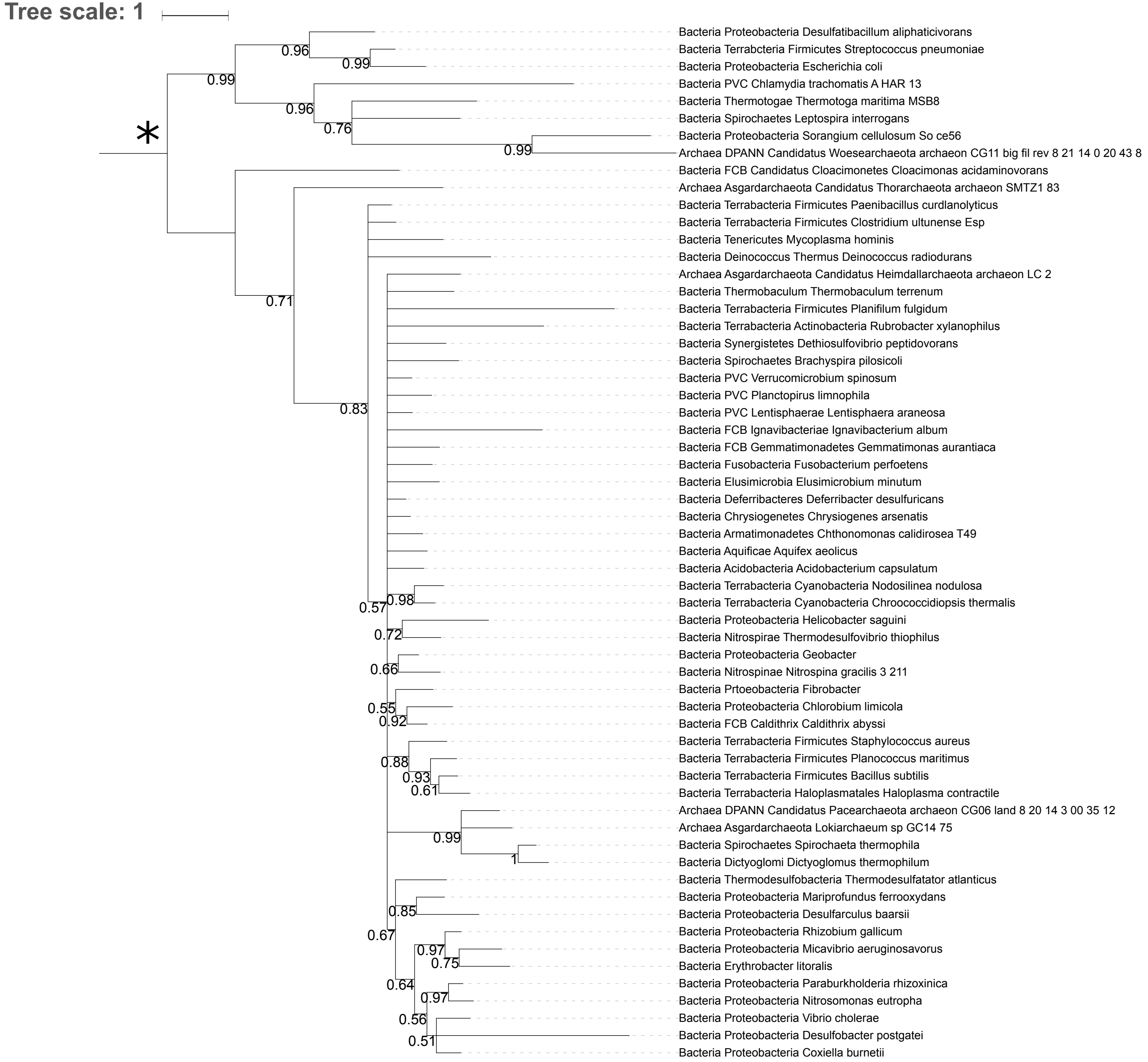

Supplementary Figure 10

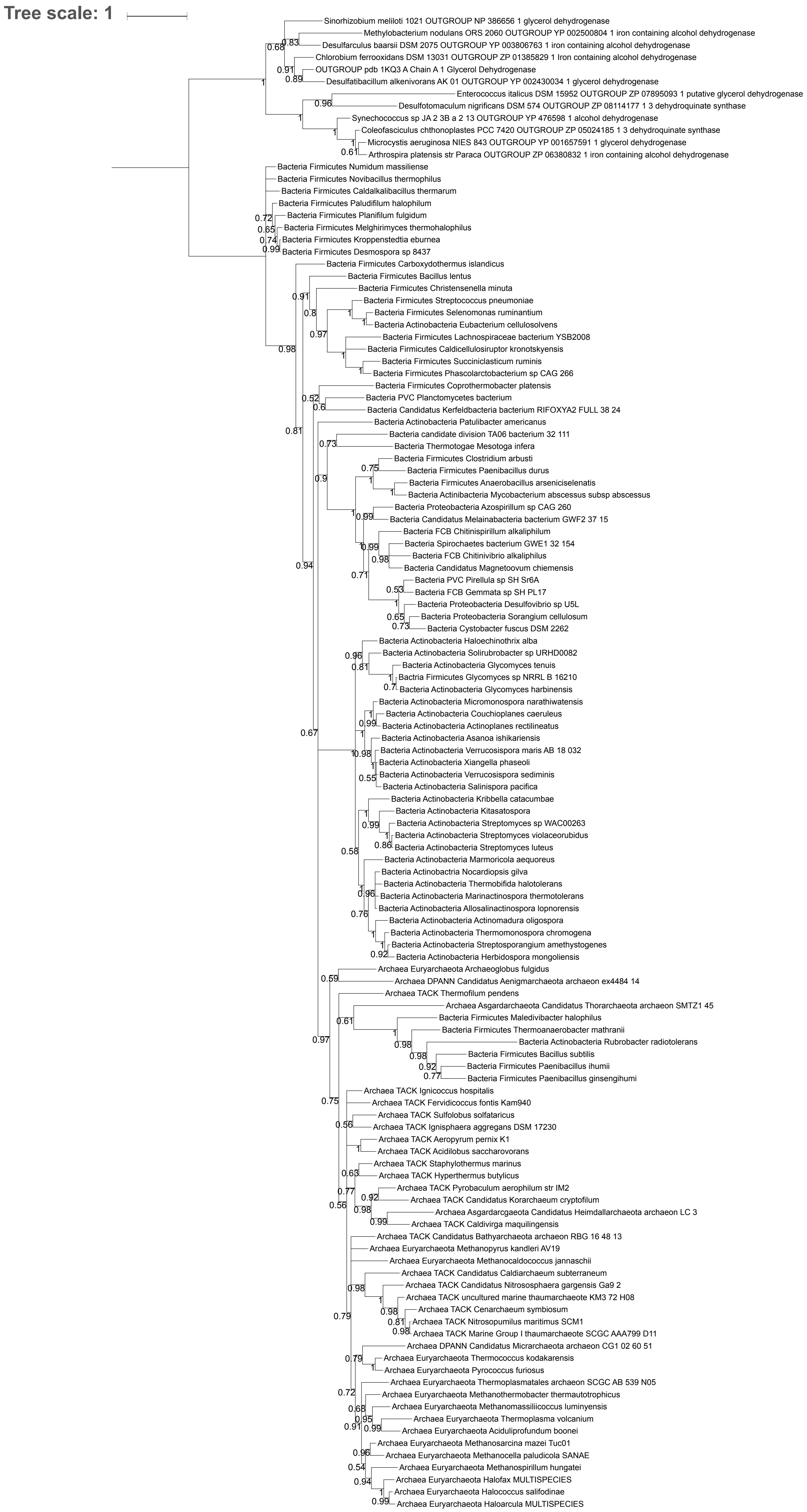

Supplementary Figure 11

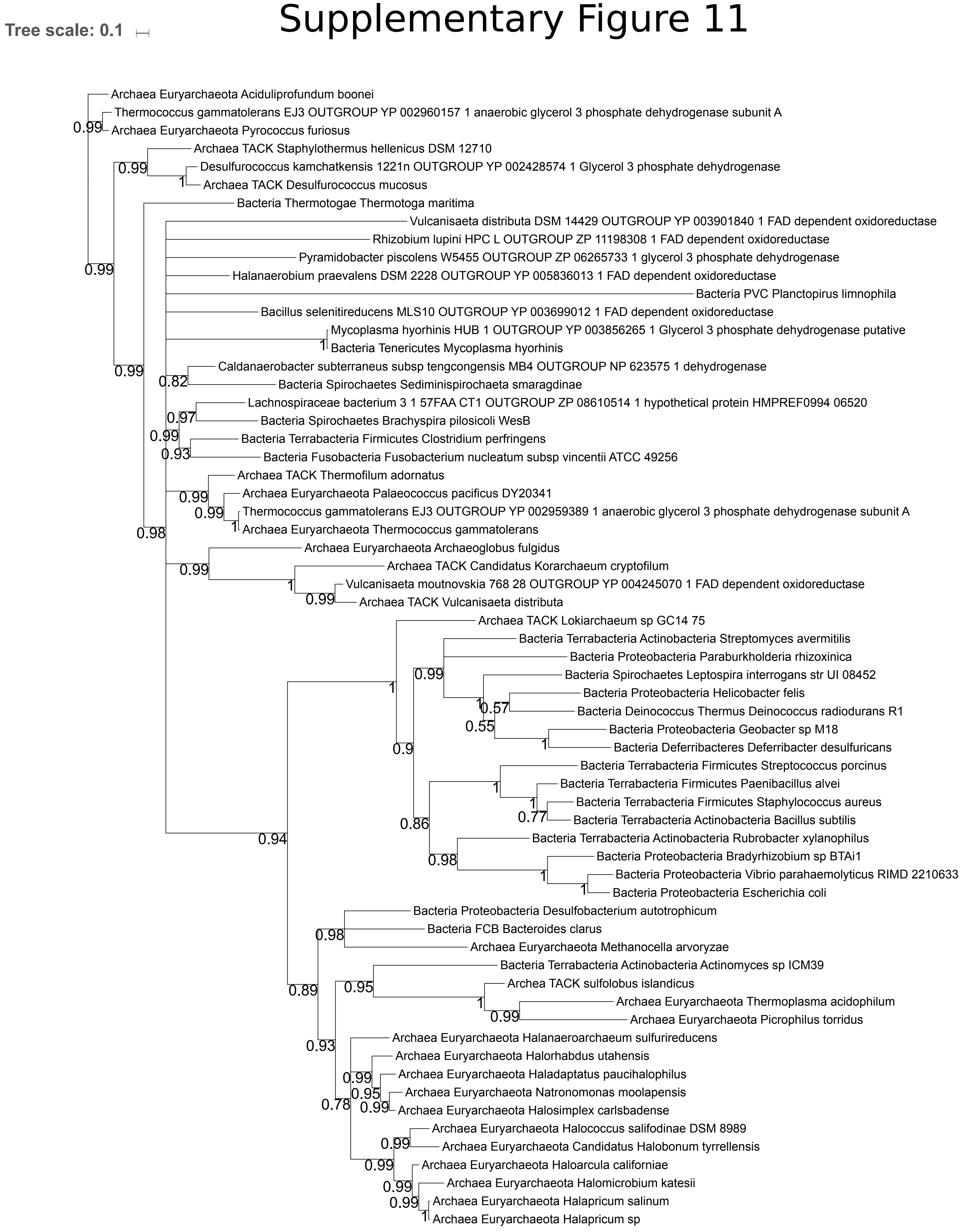

Supplementary Figure 12

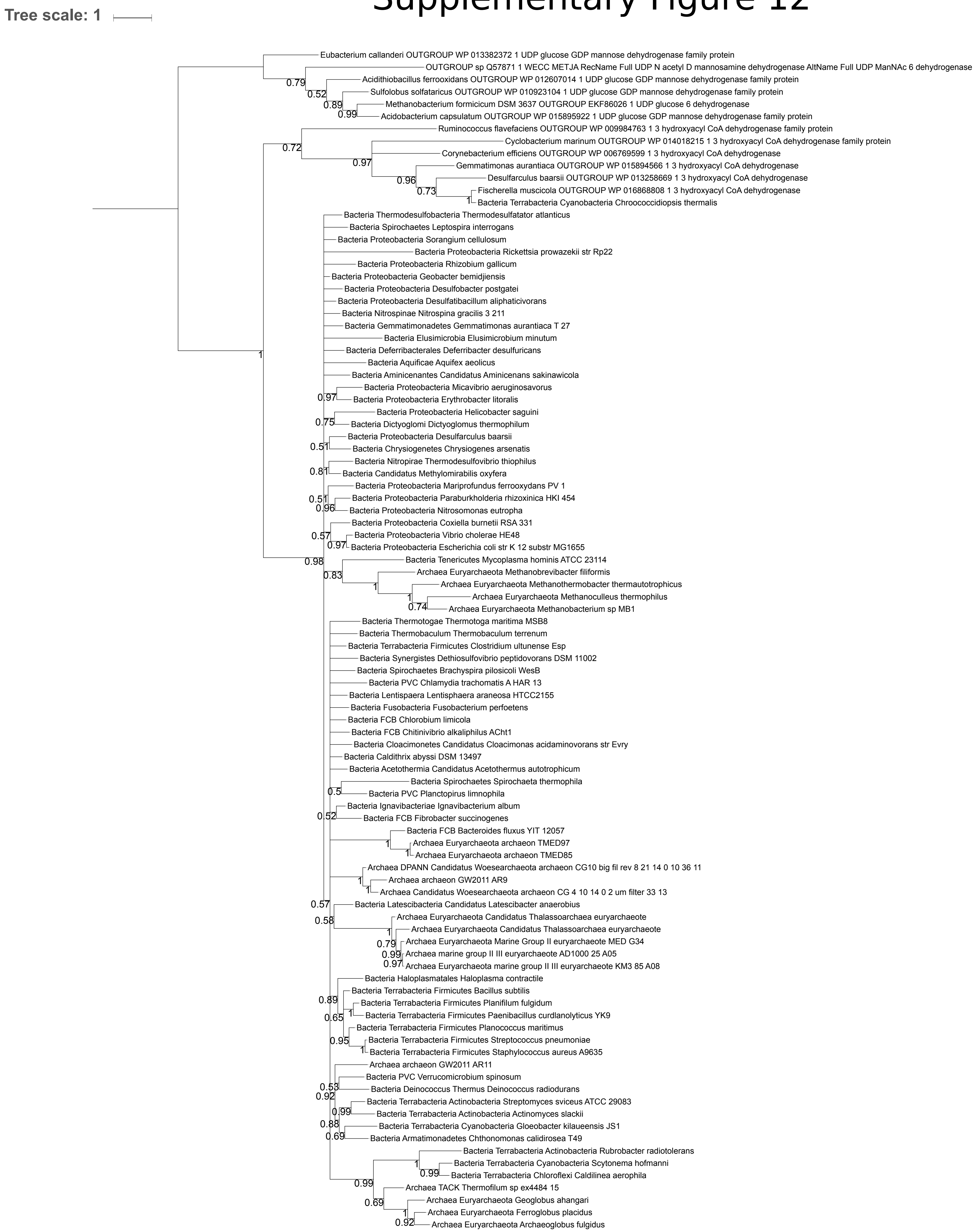

Supplementary Figure 13

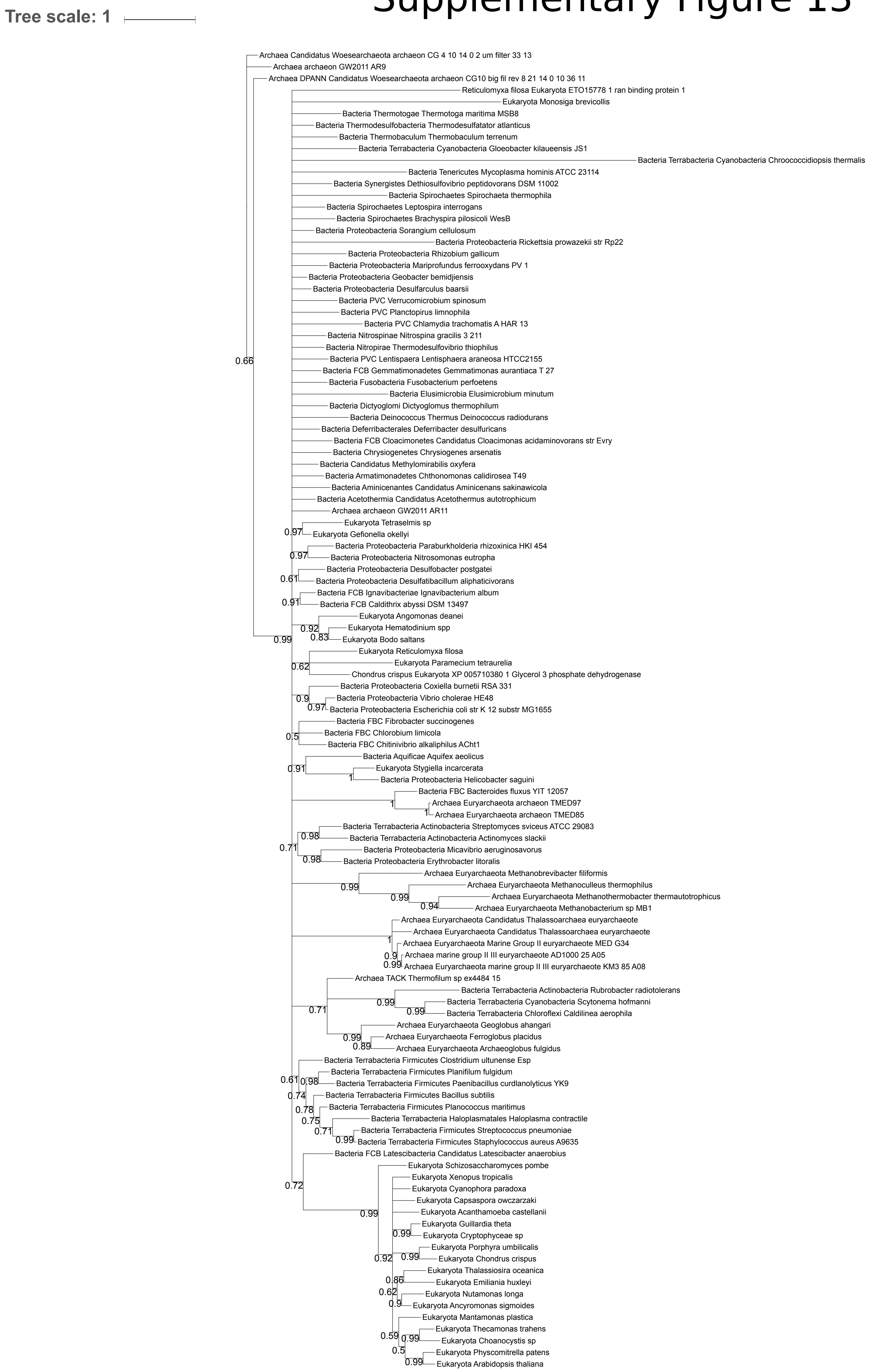

Supplementary Figure 14

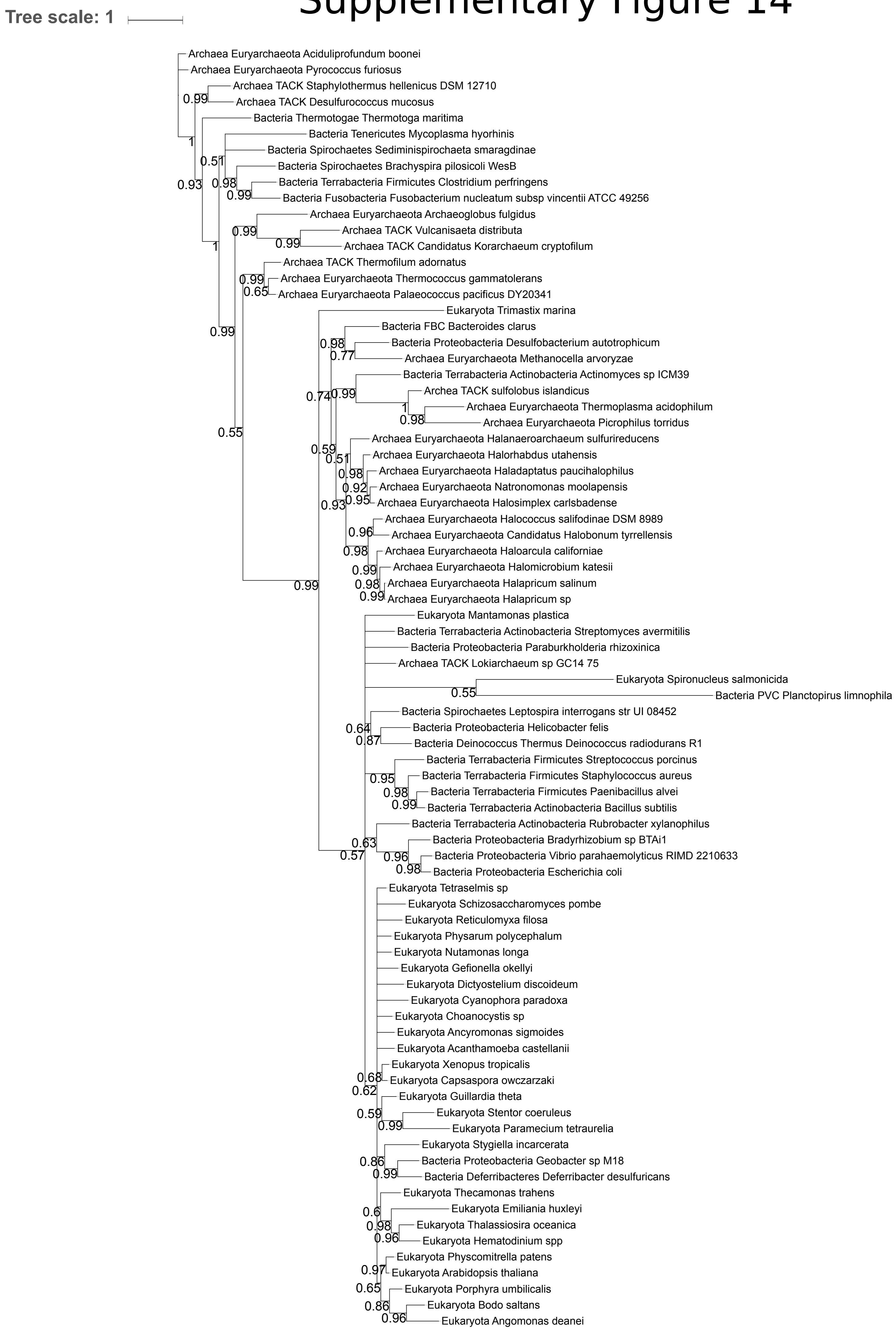

Supplementary Figure 15

Tree scale: 1

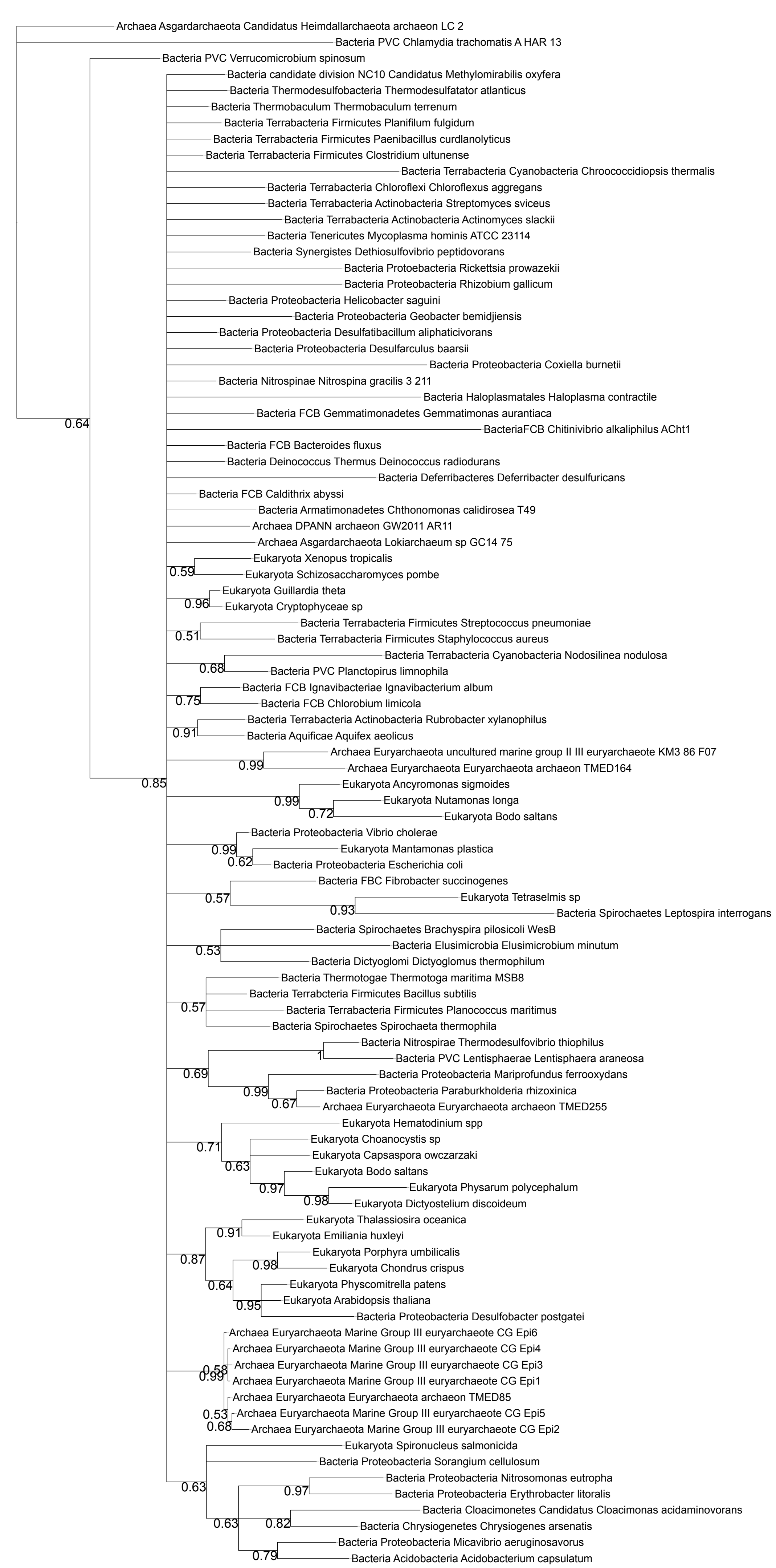

Tree scale: 1

Supplementary Figure 16

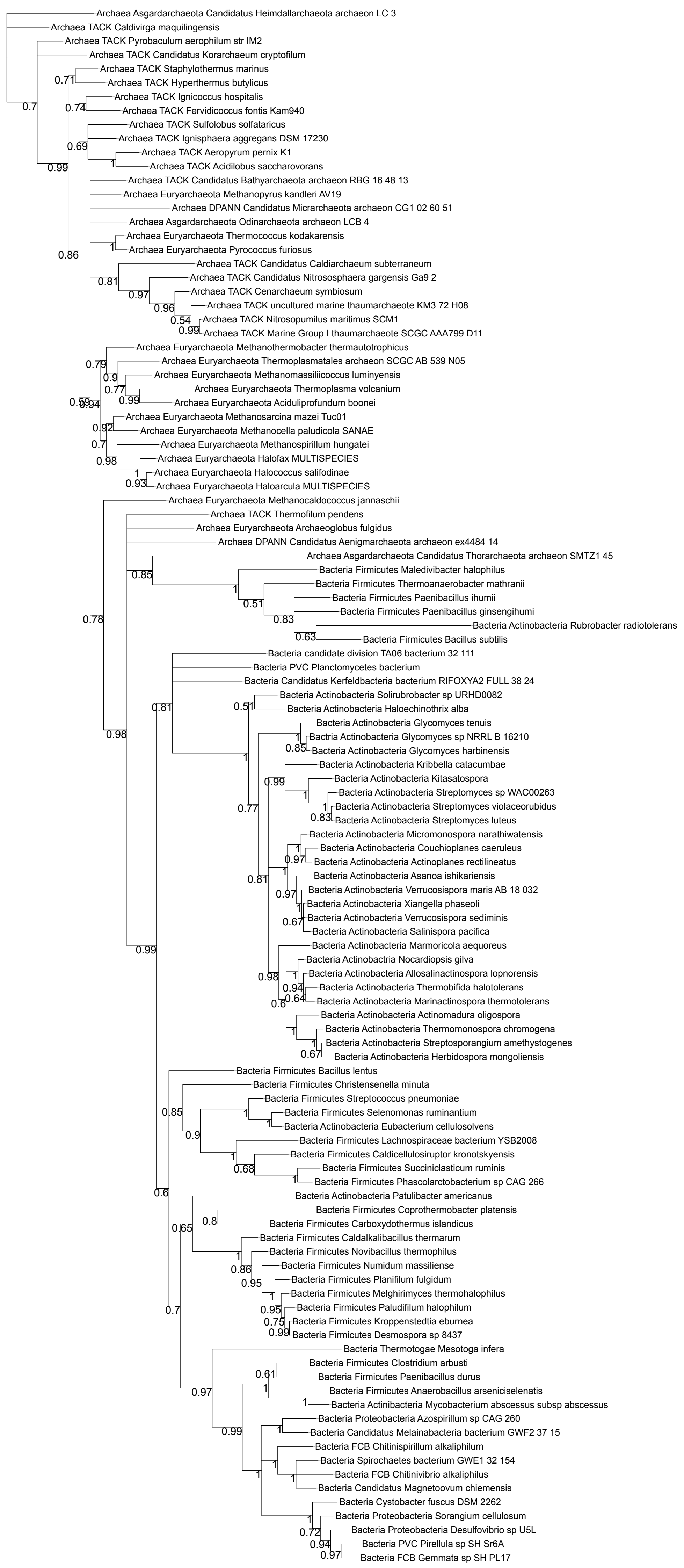

Supplementary Figure 17

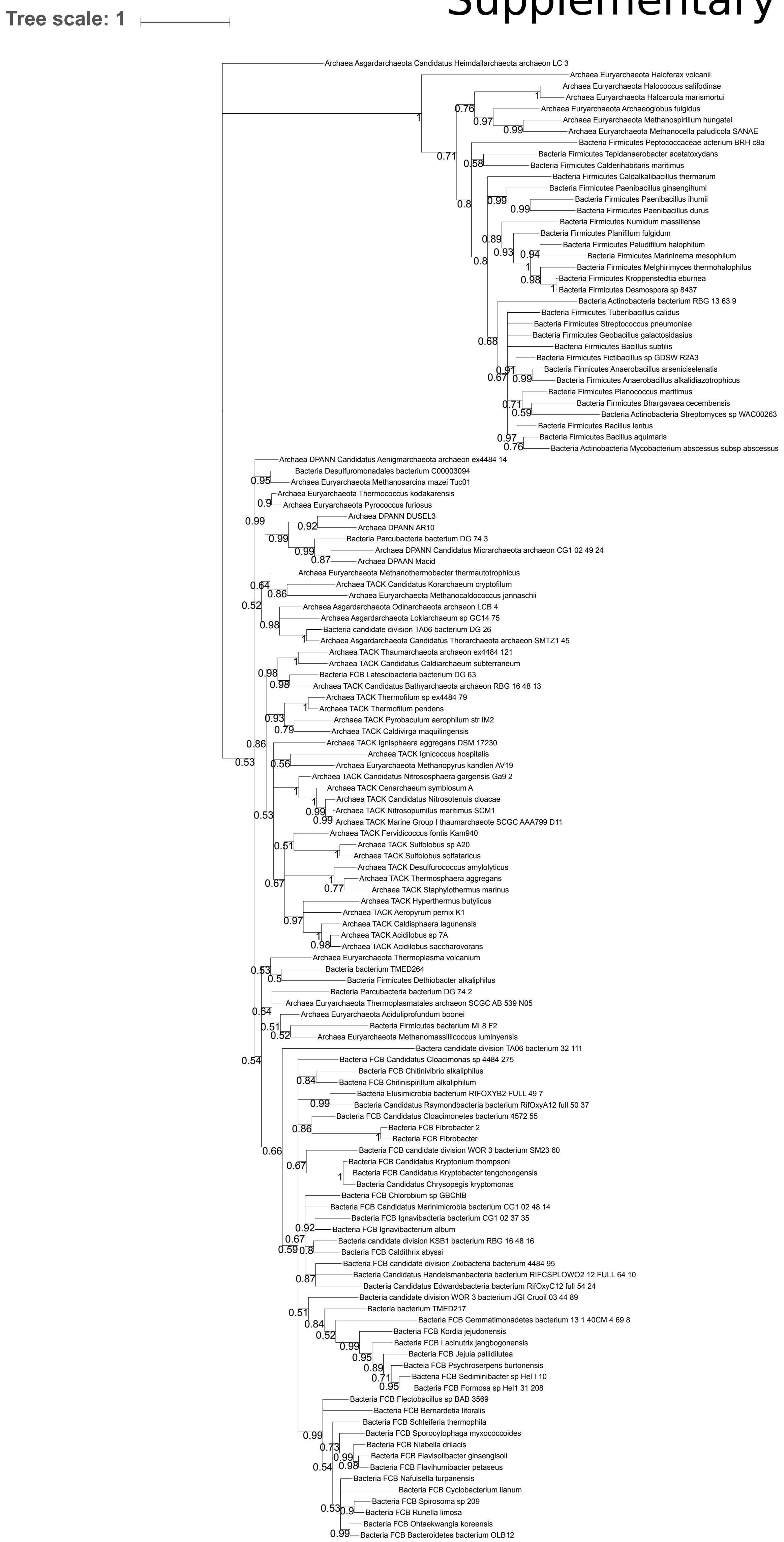

Supplementary Figure 18

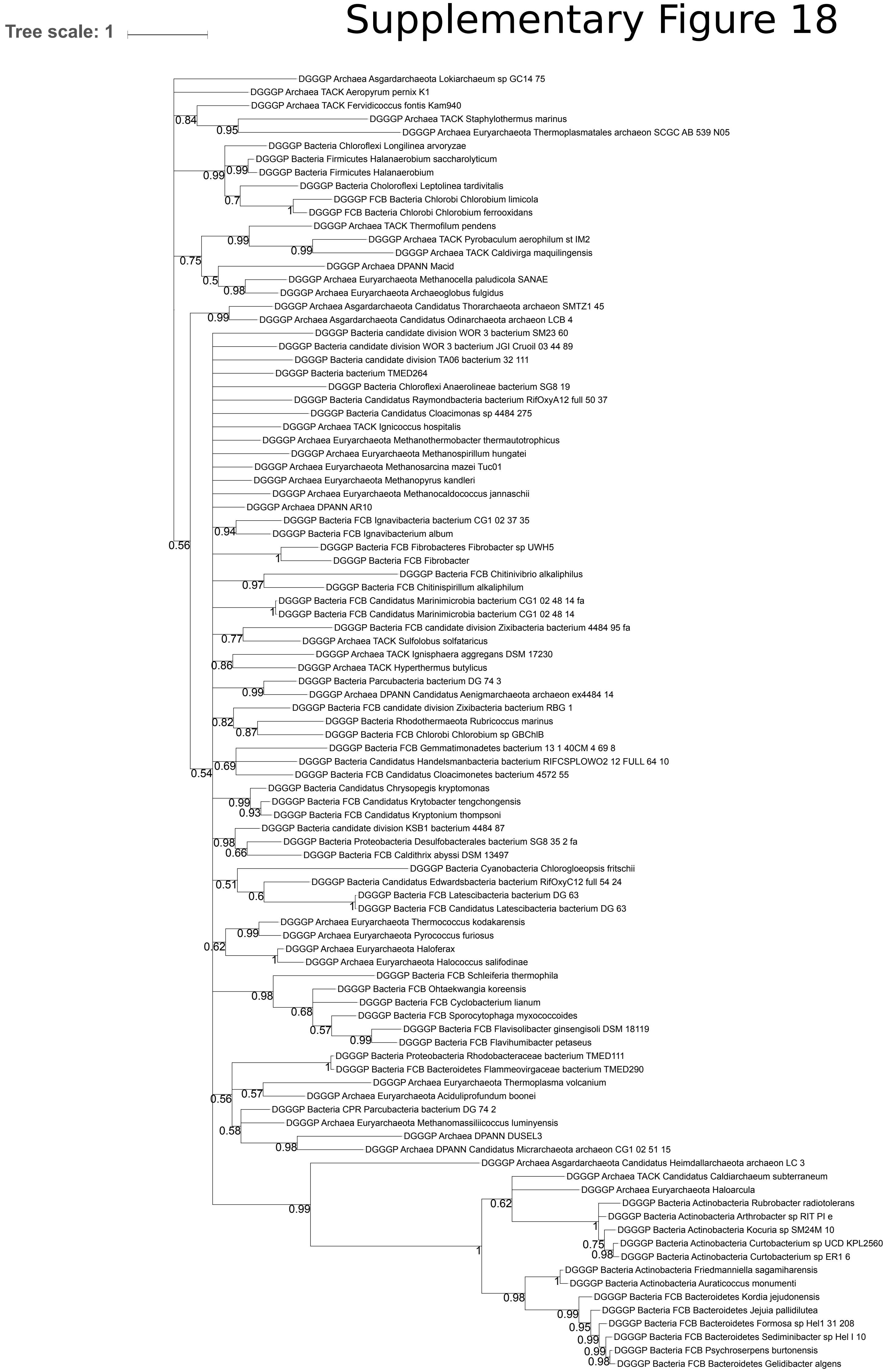

Supplementary Figure 19

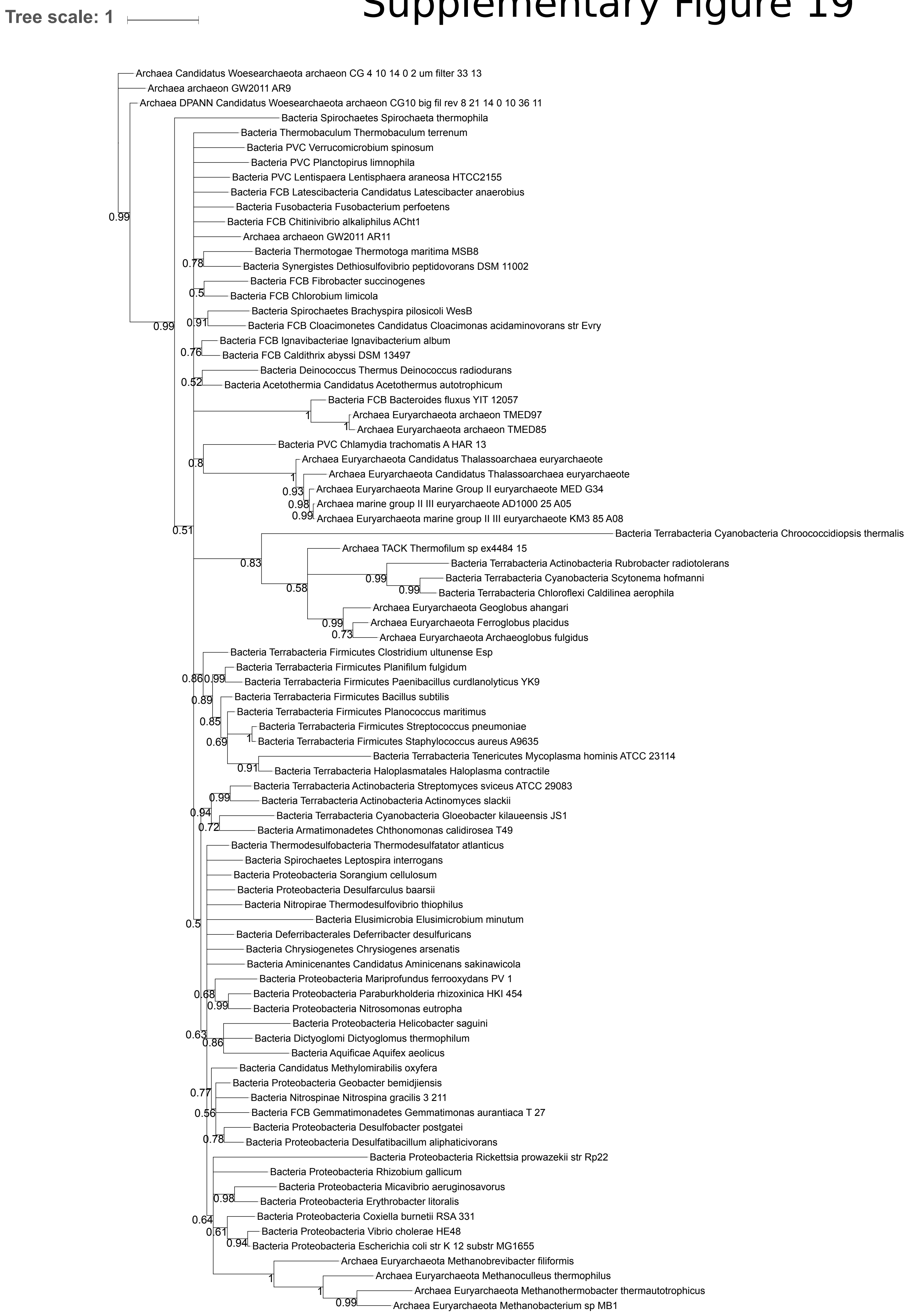

Supplementary Figure 20

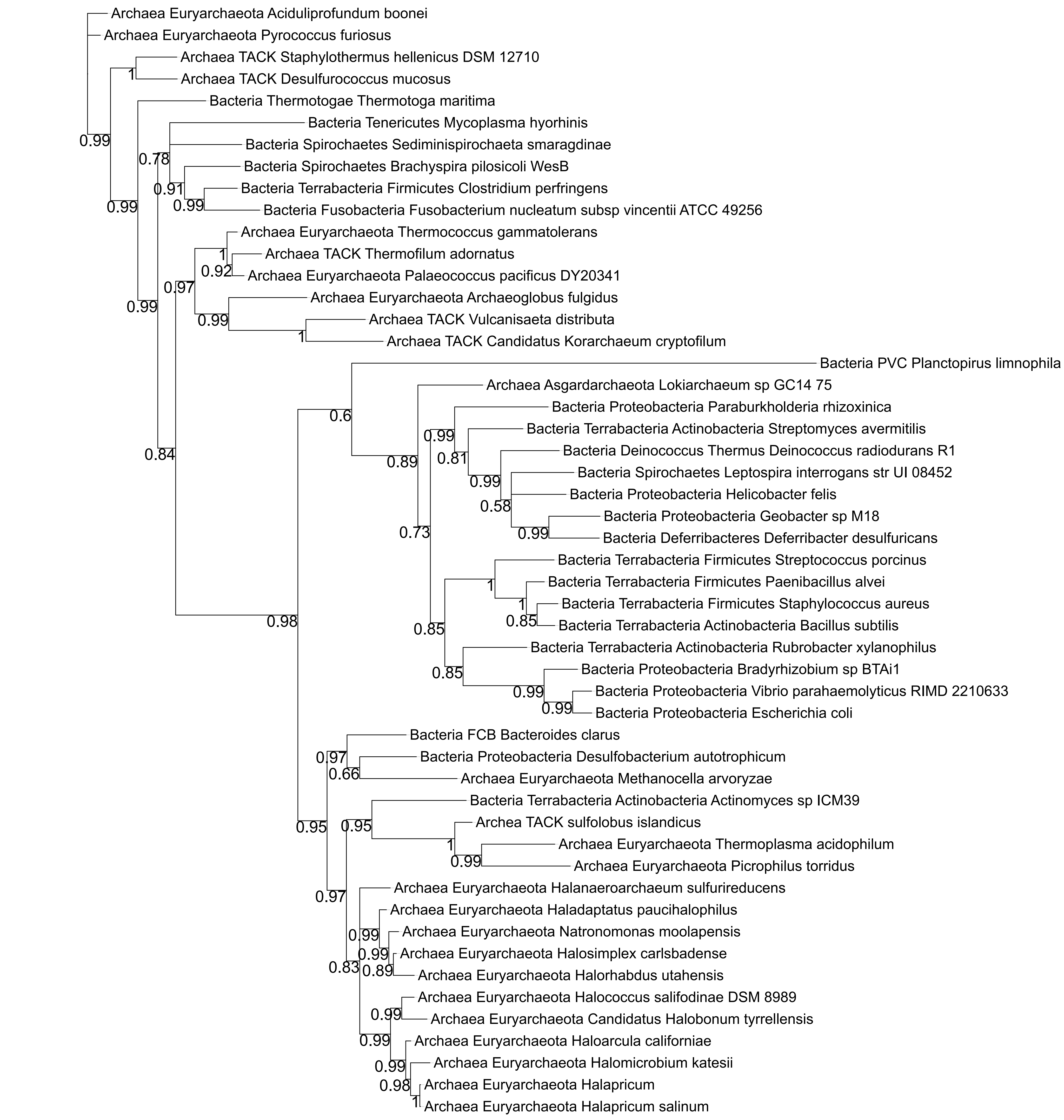

Supplementary Figure 21

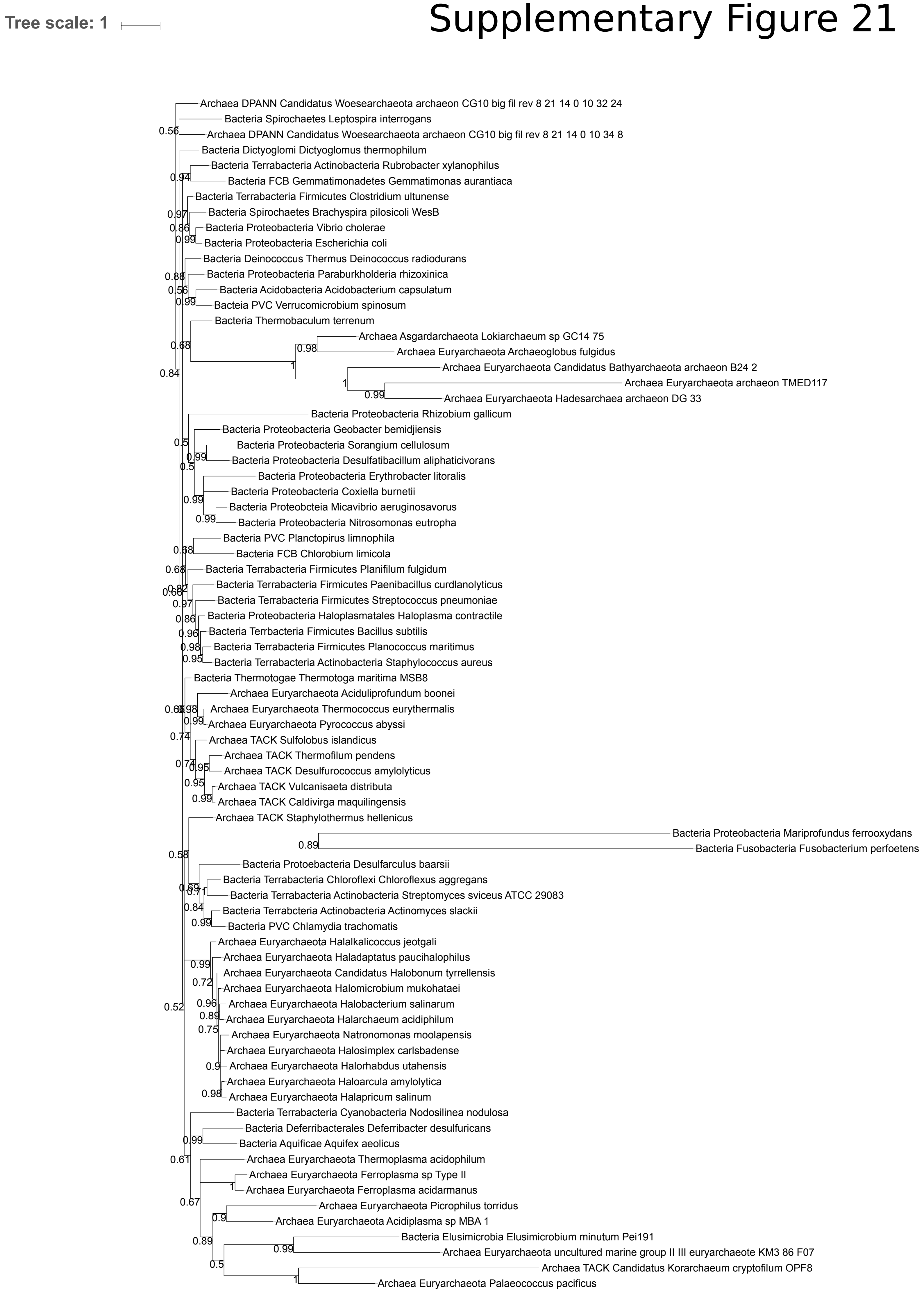

Supplementary Figure 22

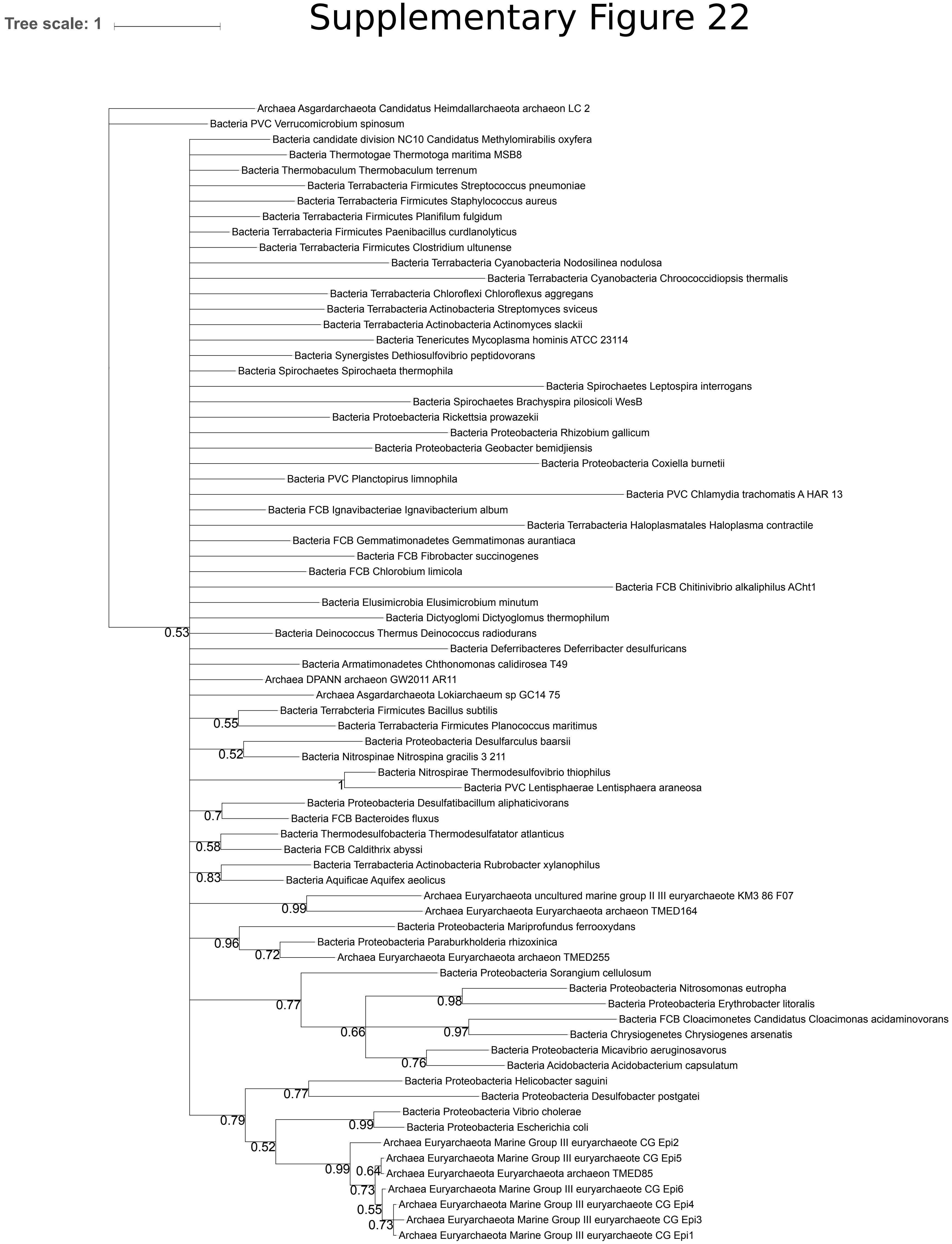

Supplementary Figure 23

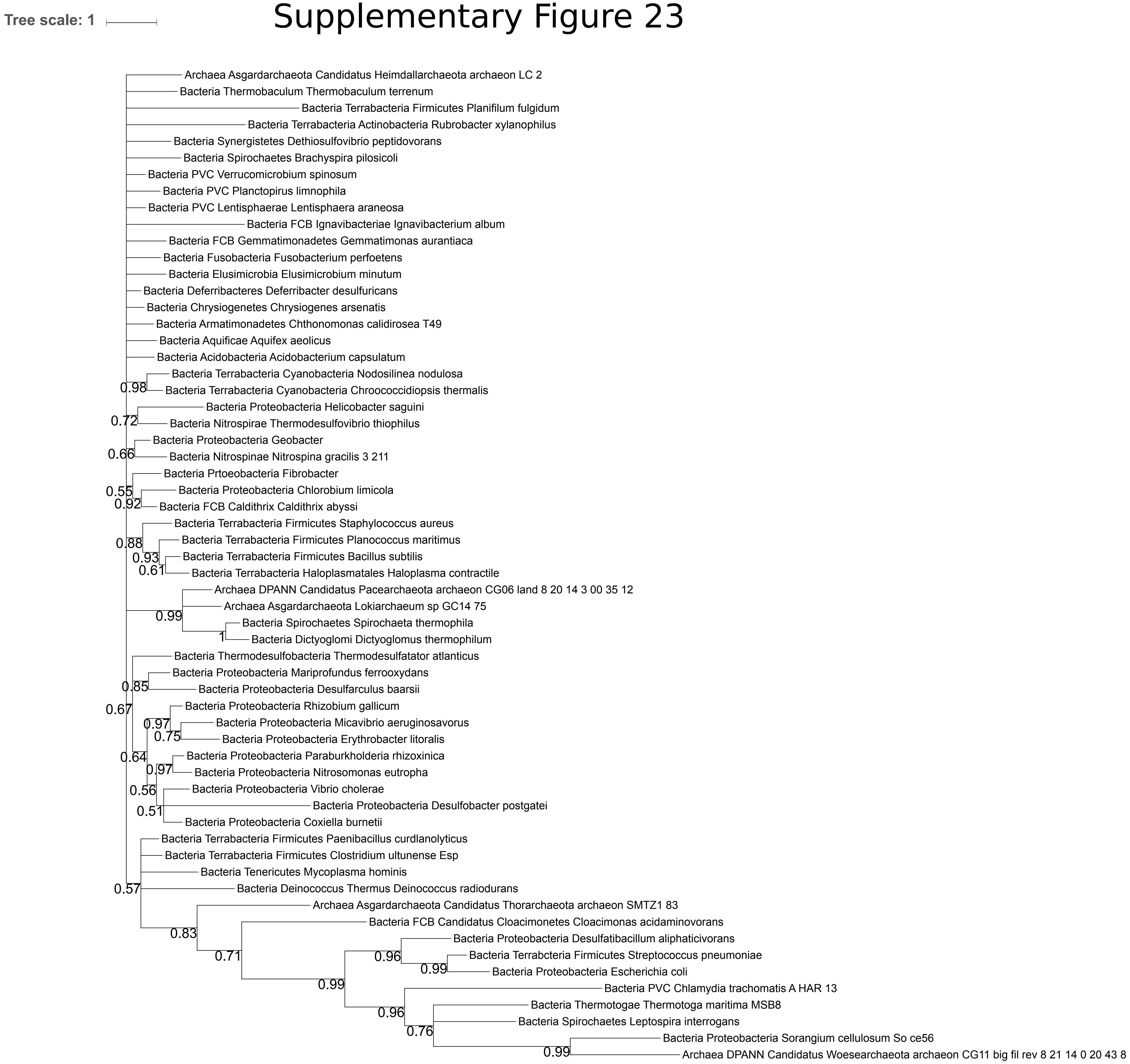

Supplementary Figure 24

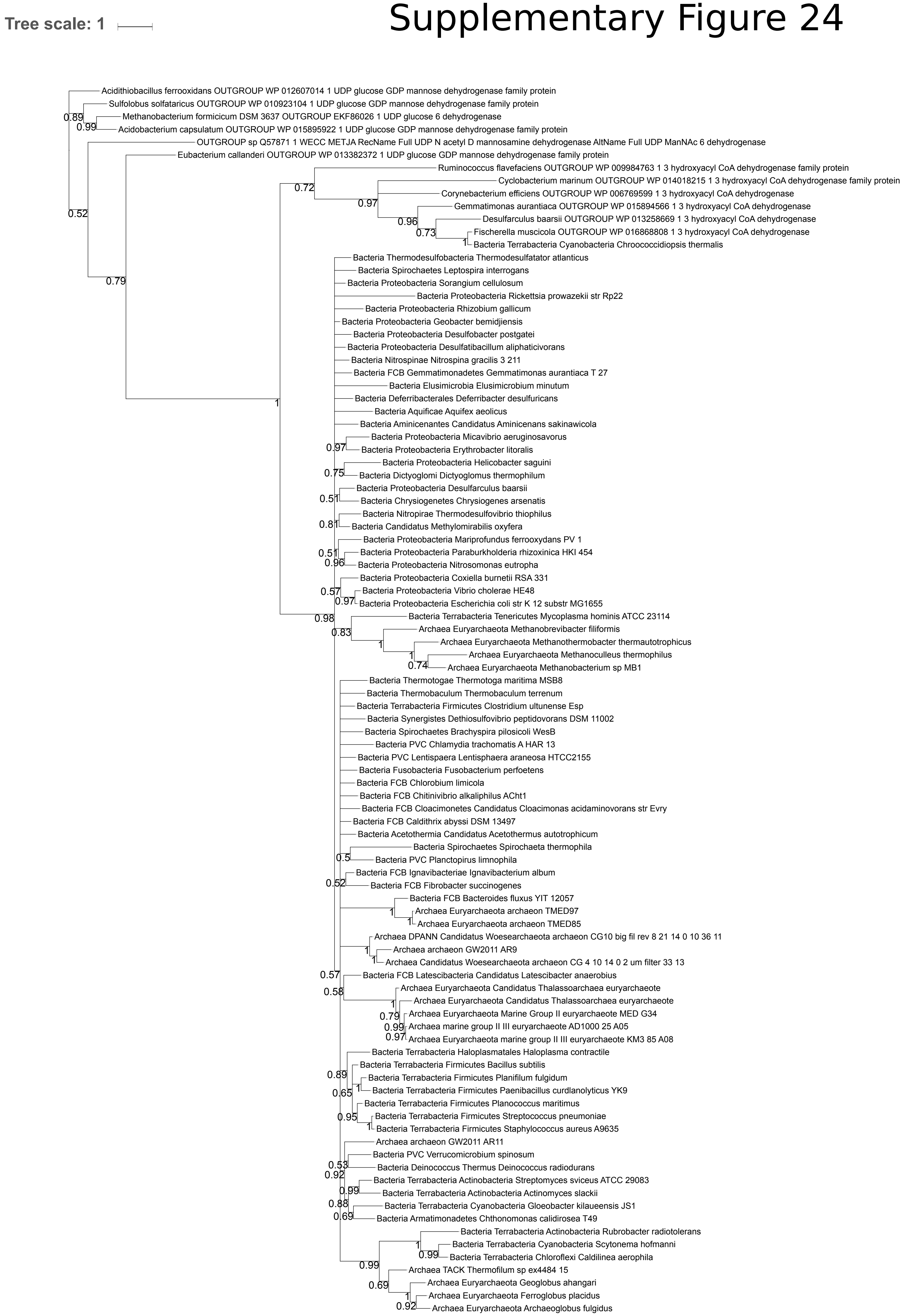

Supplementary Figure 25

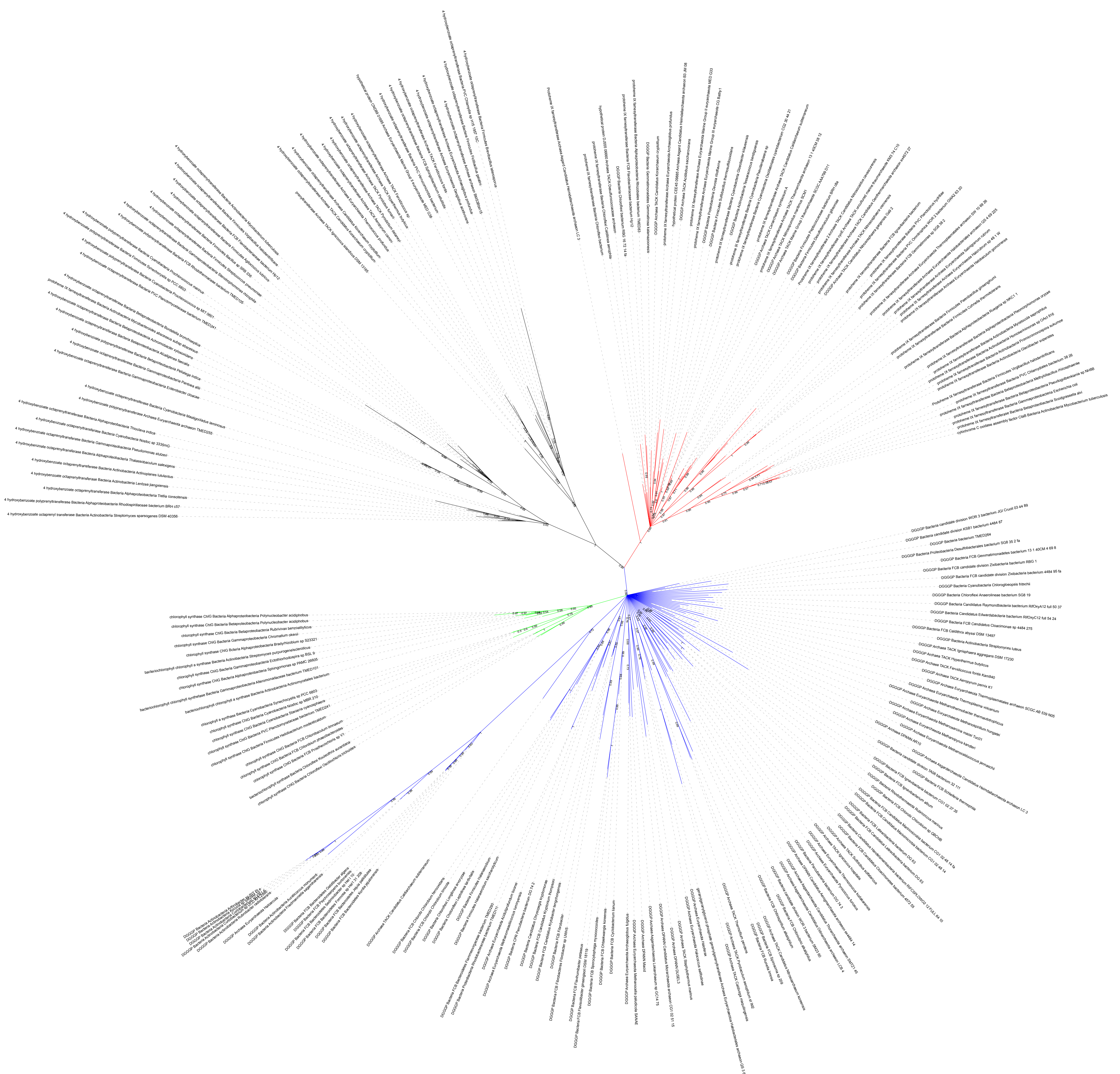

Supplementary Figure 26

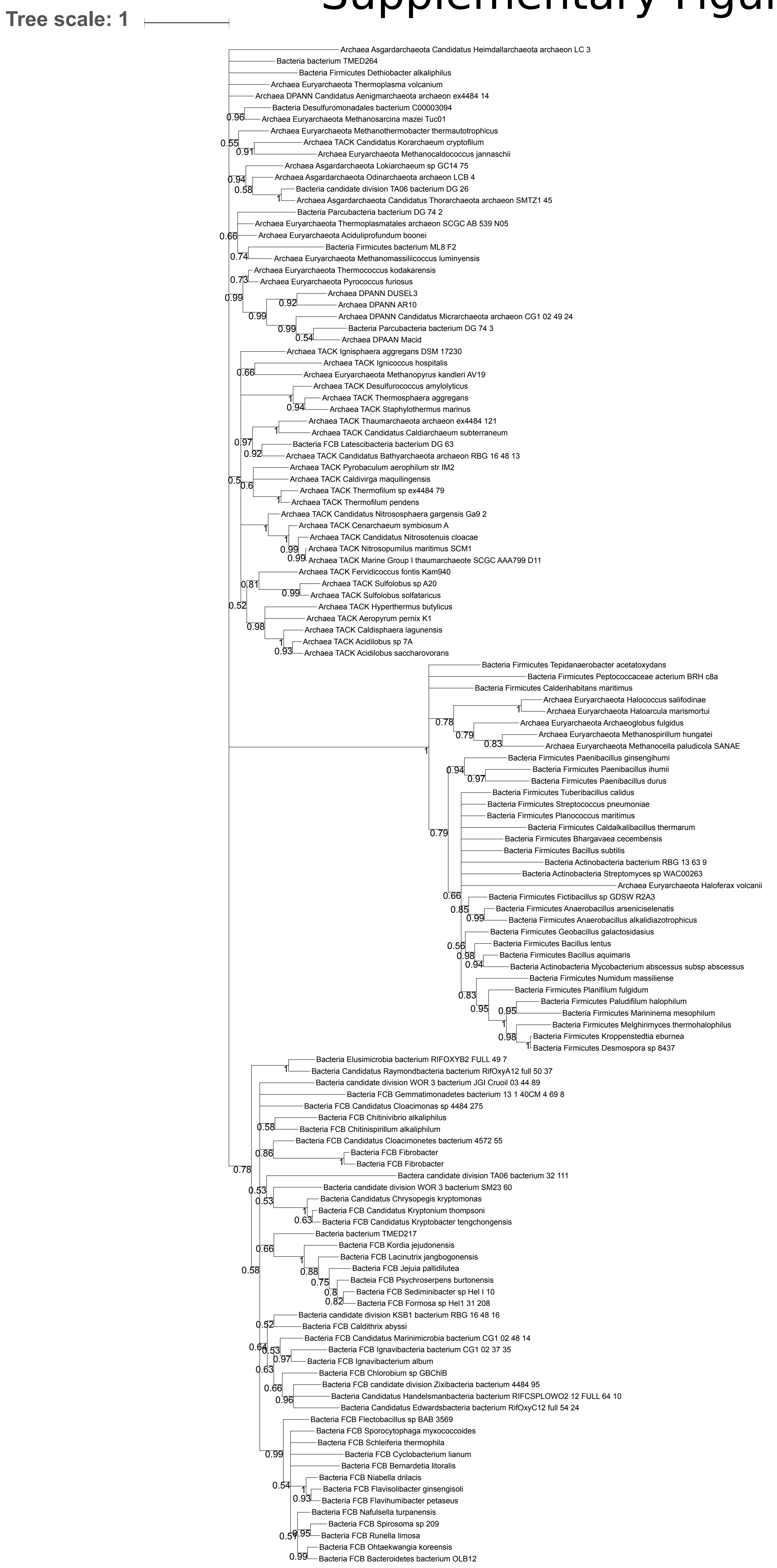

Supplementary Figure 27

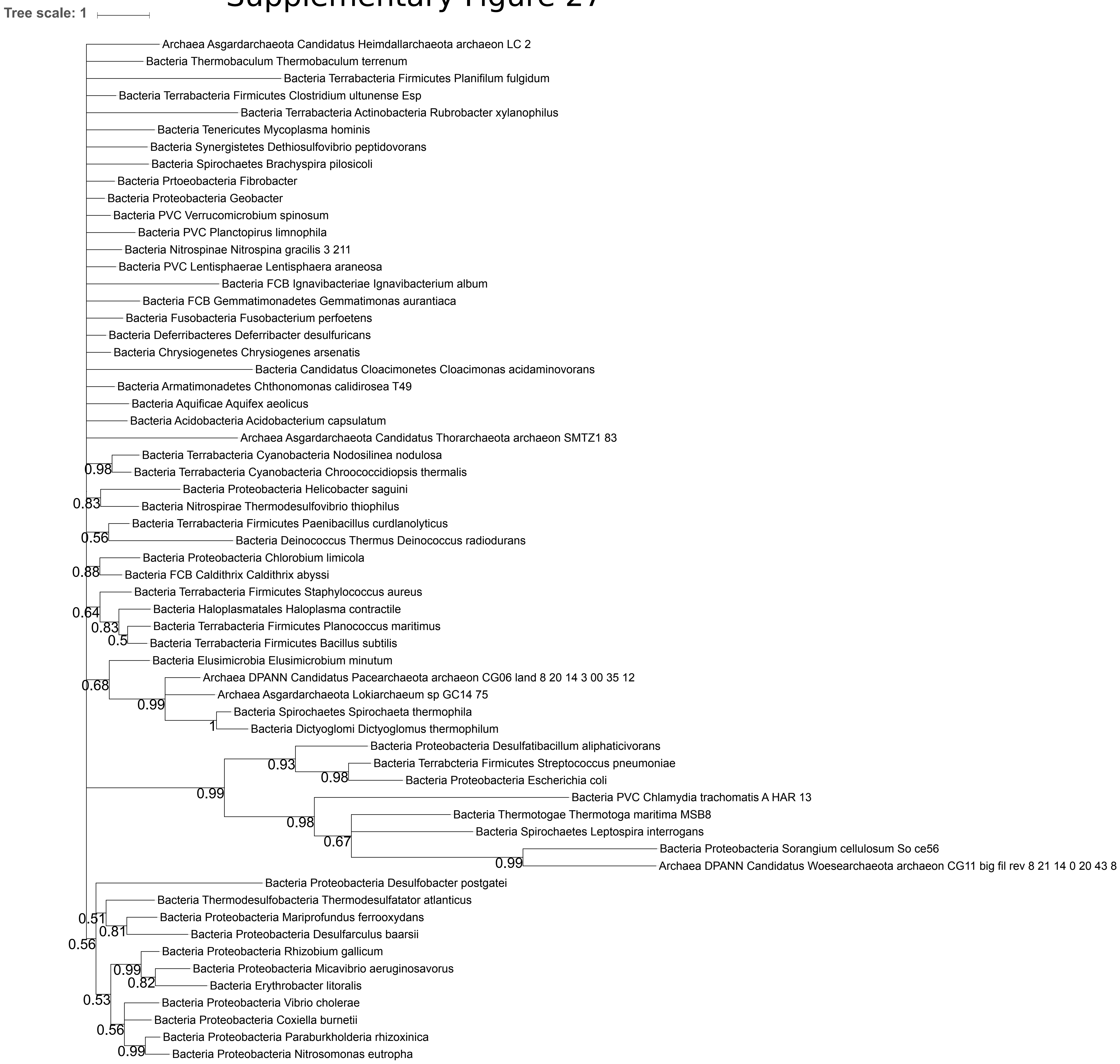

Supplementary Figure 28

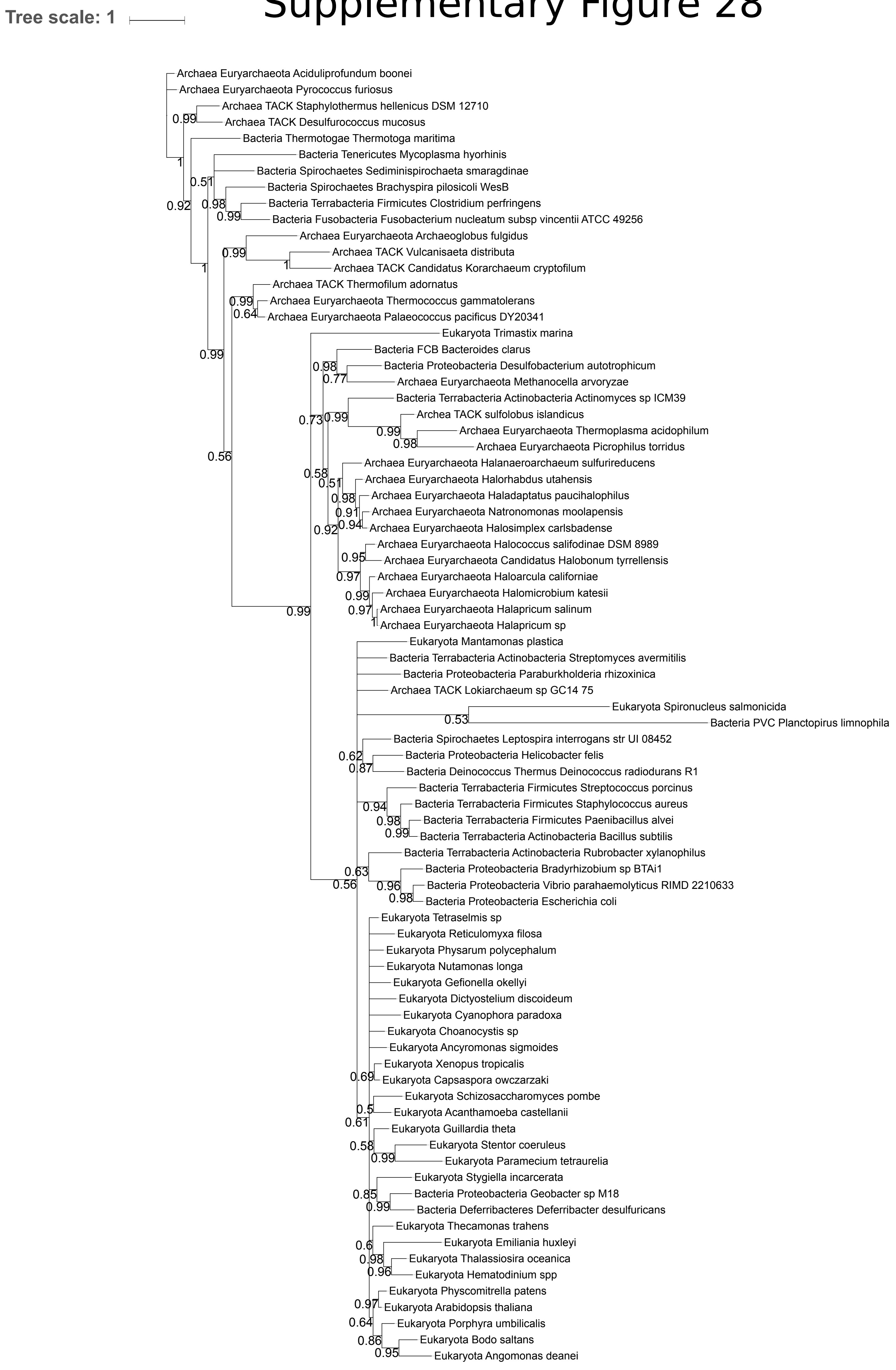

Supplementary Figure 29

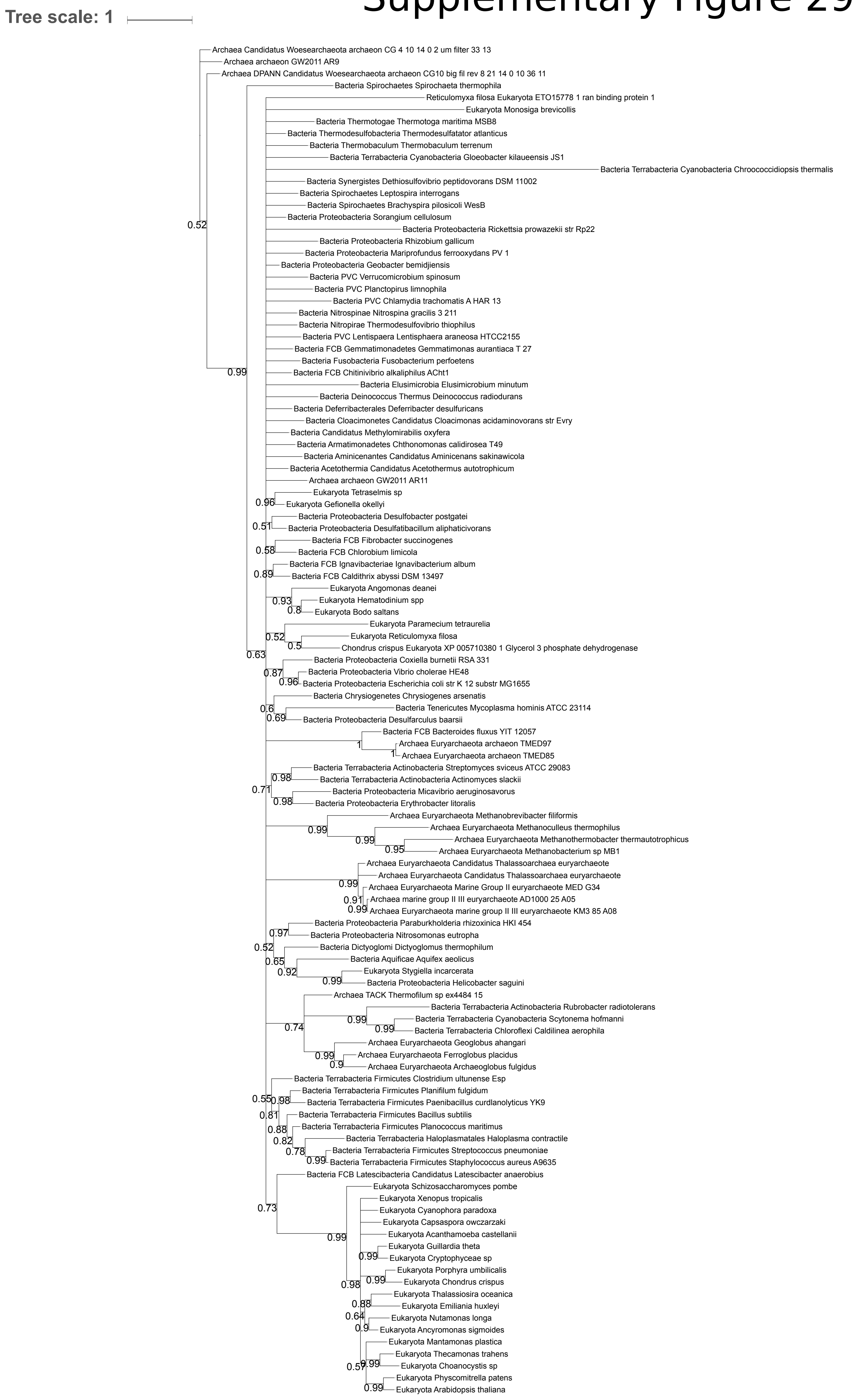

Supplementary Figure 30

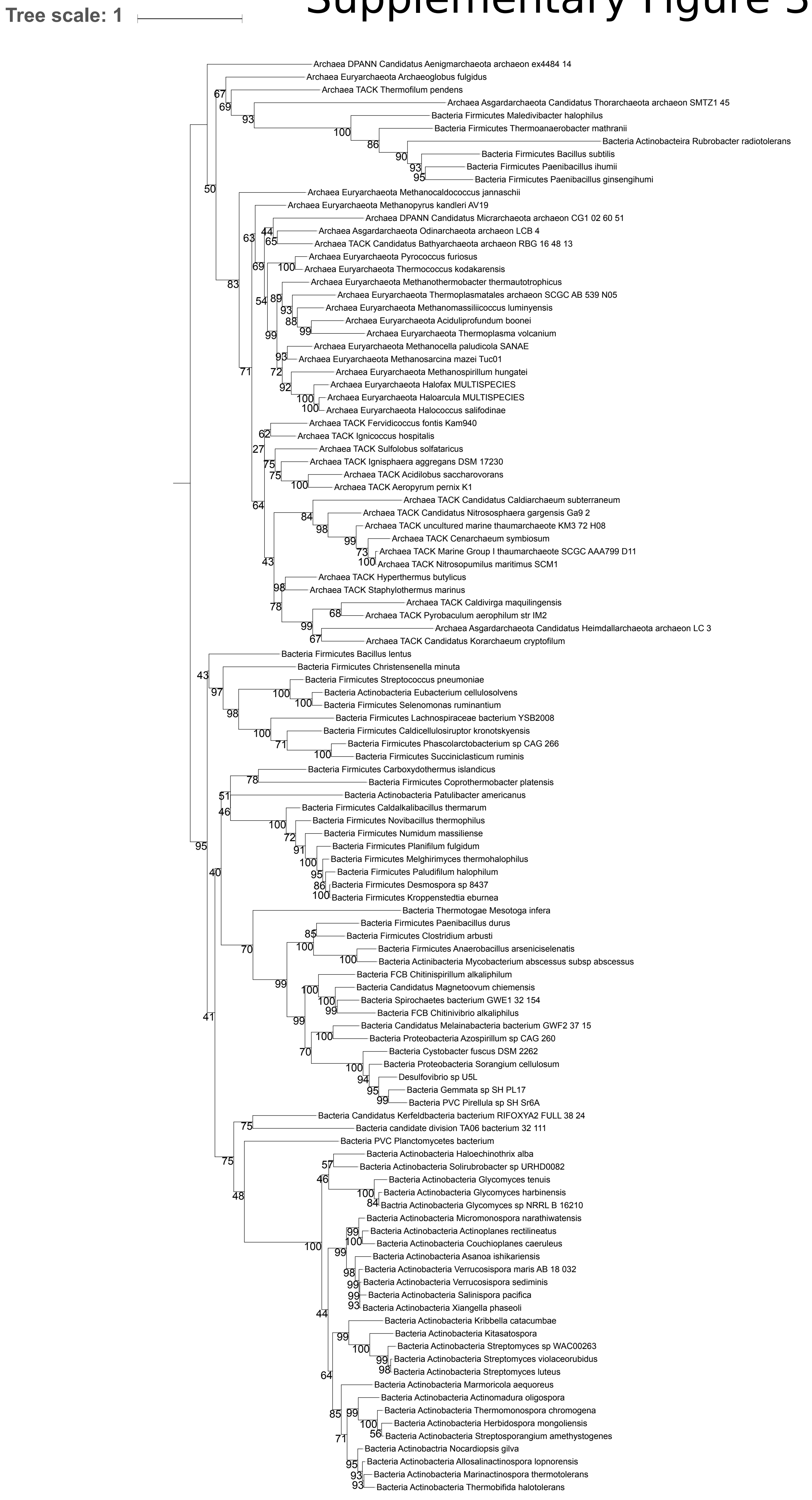

Supplementary Figure 31

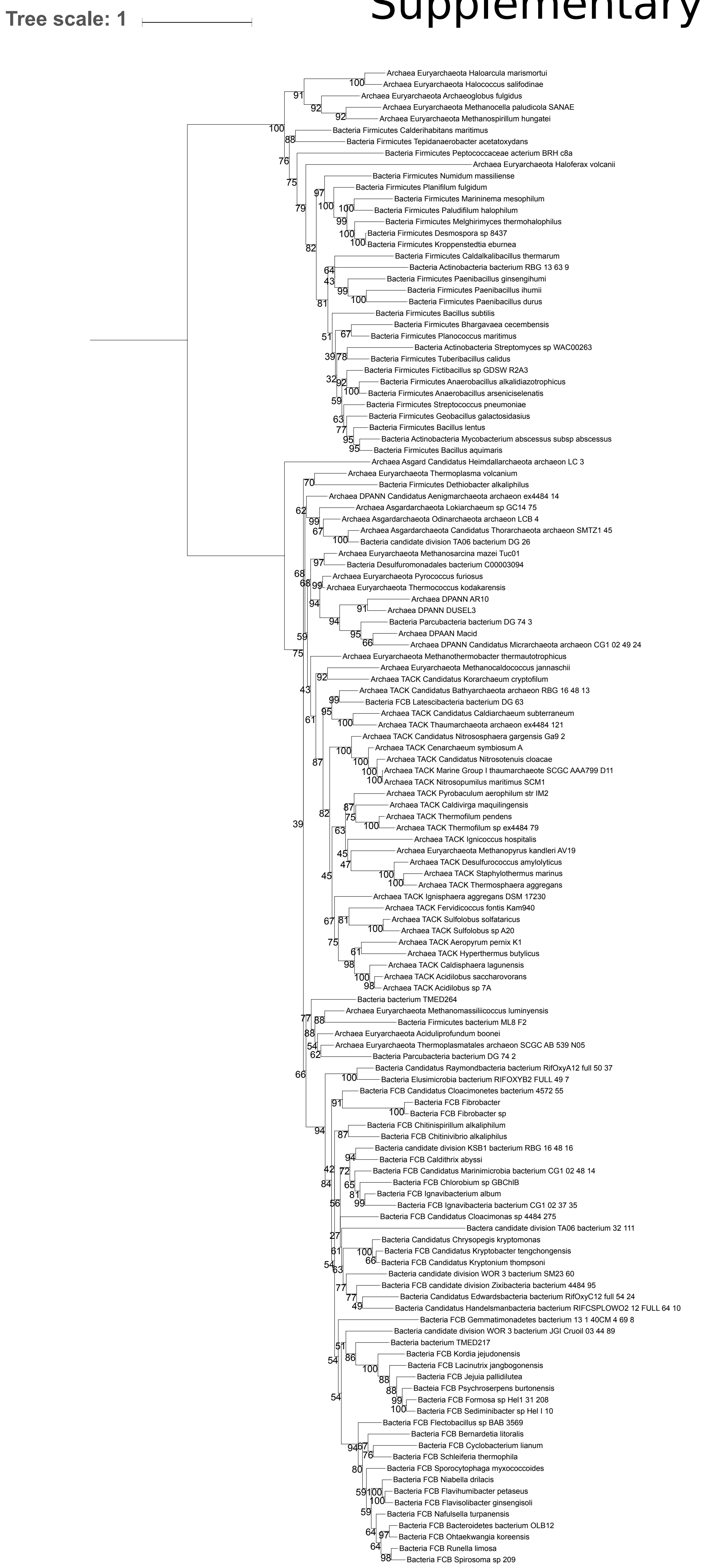

Supplementary Figure 32

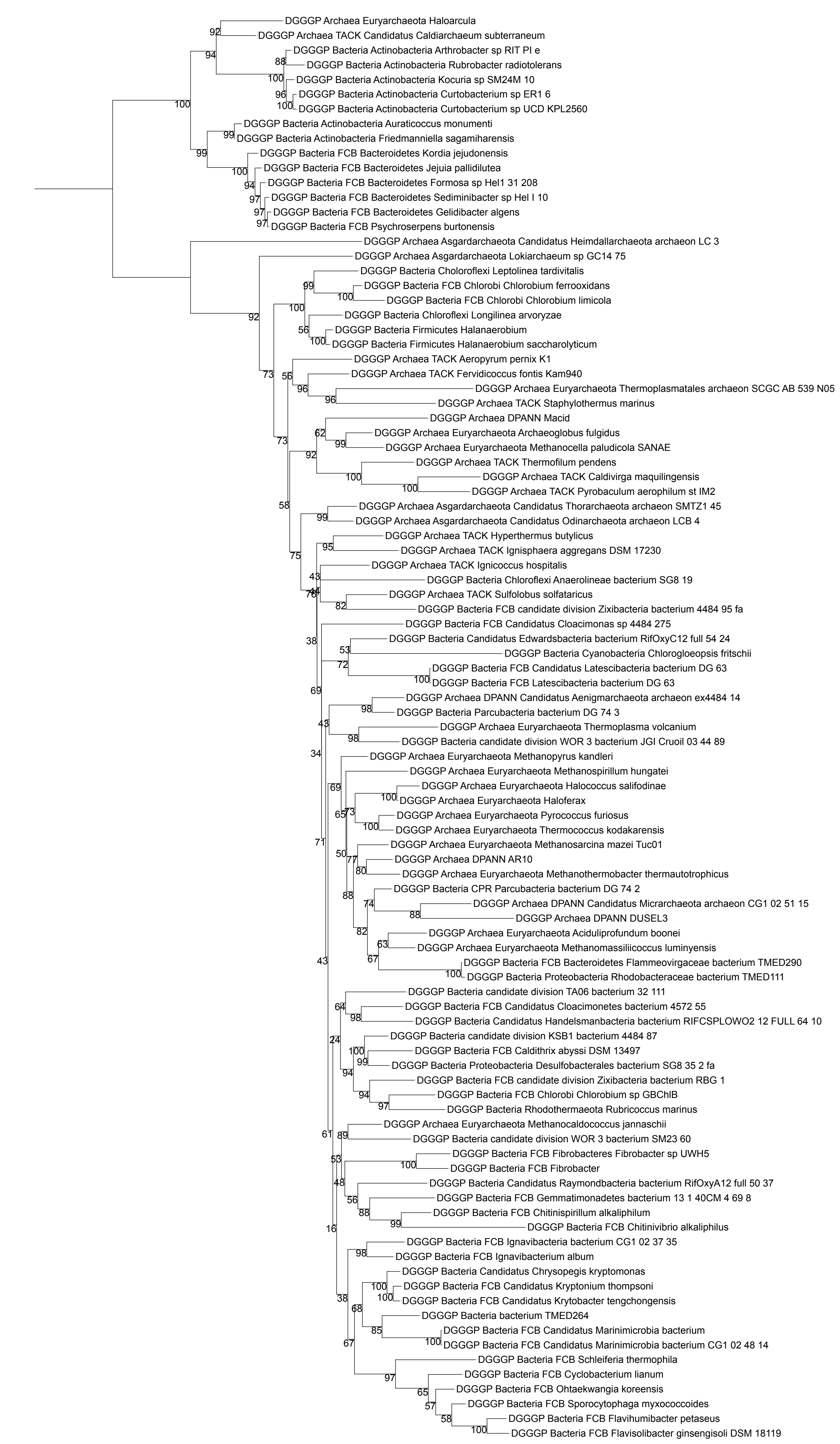

Supplementary Figure 33

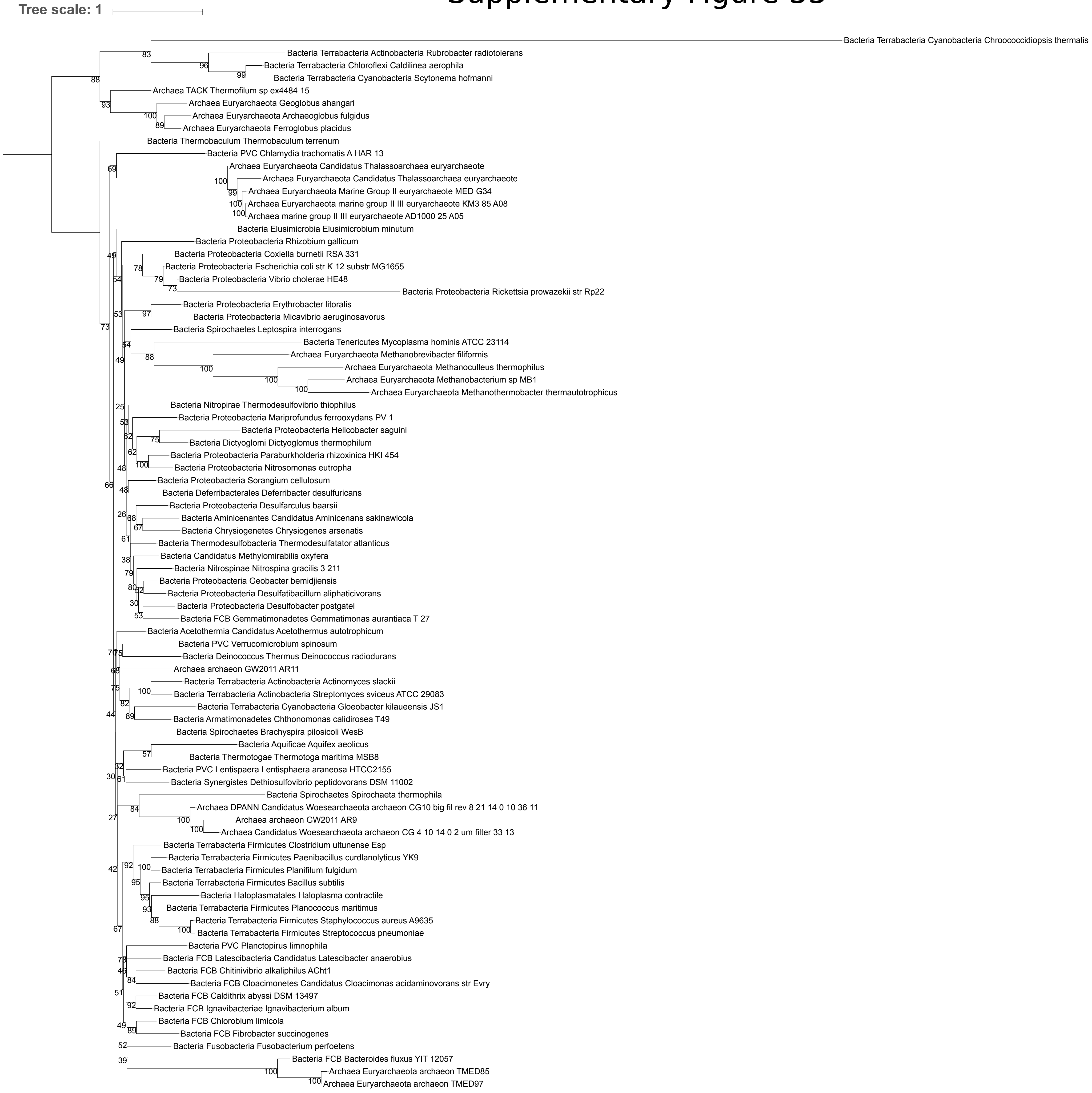

Supplementary Figure 34

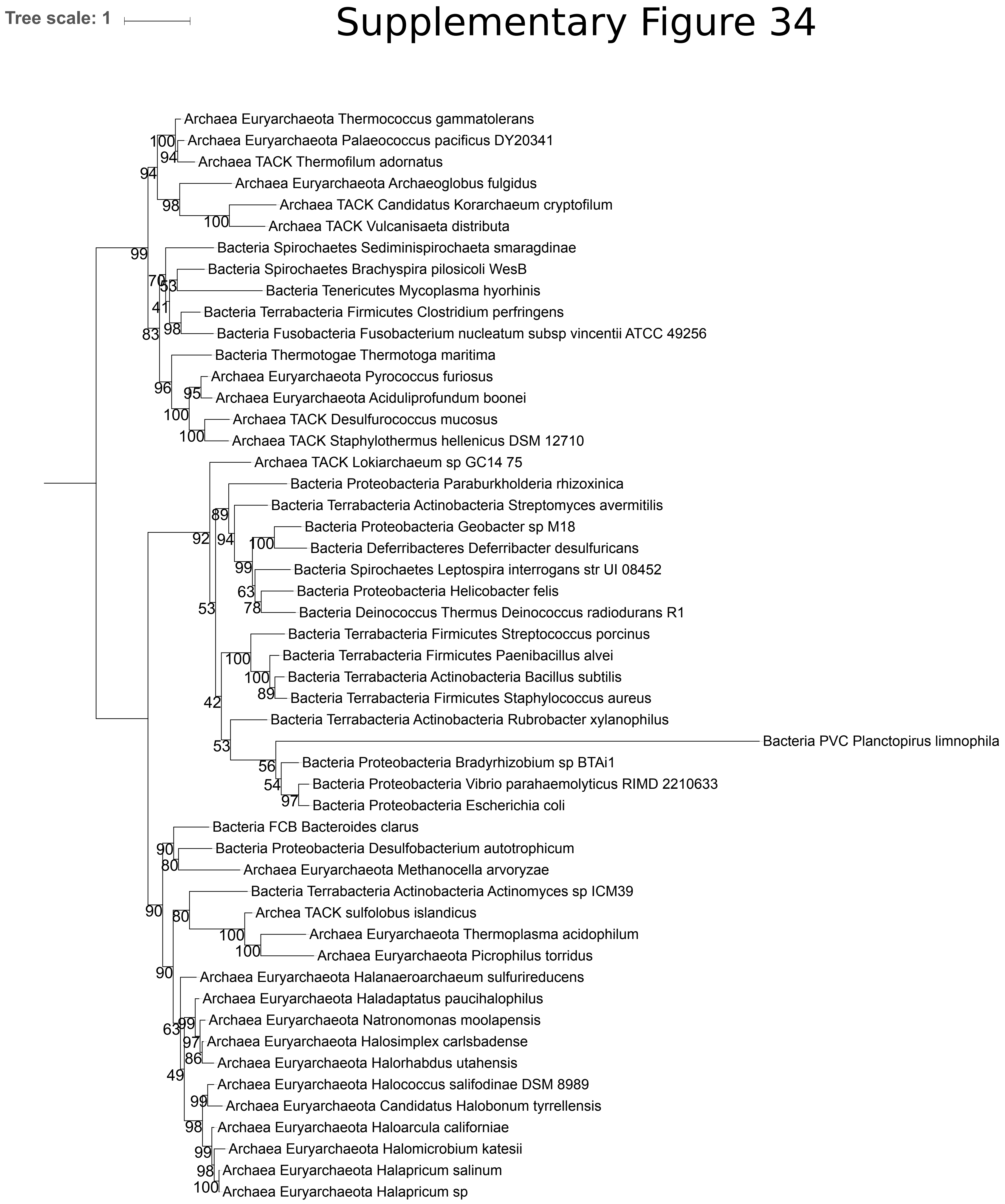

Supplementary Figure 35

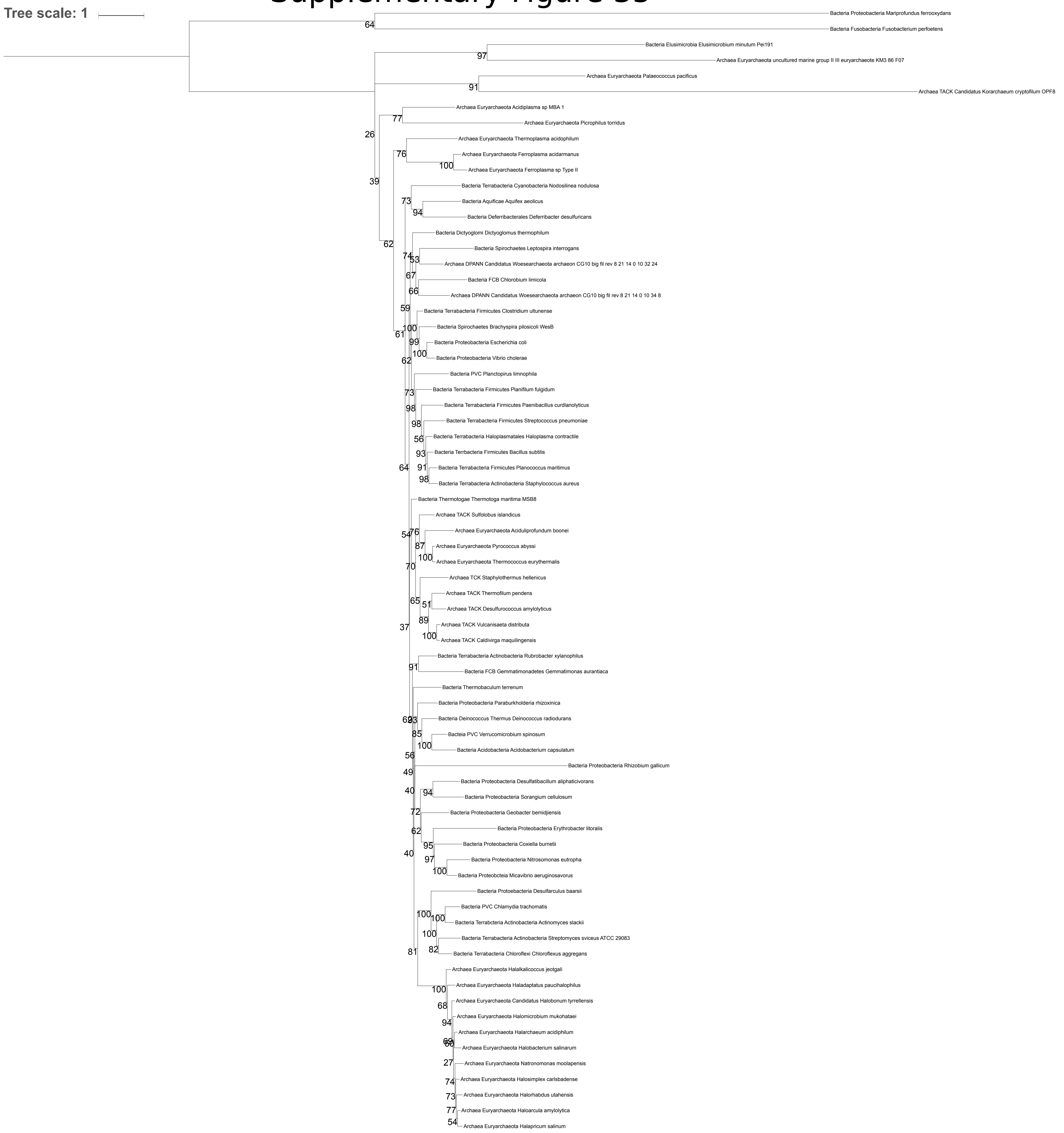

Supplementary Figure 36

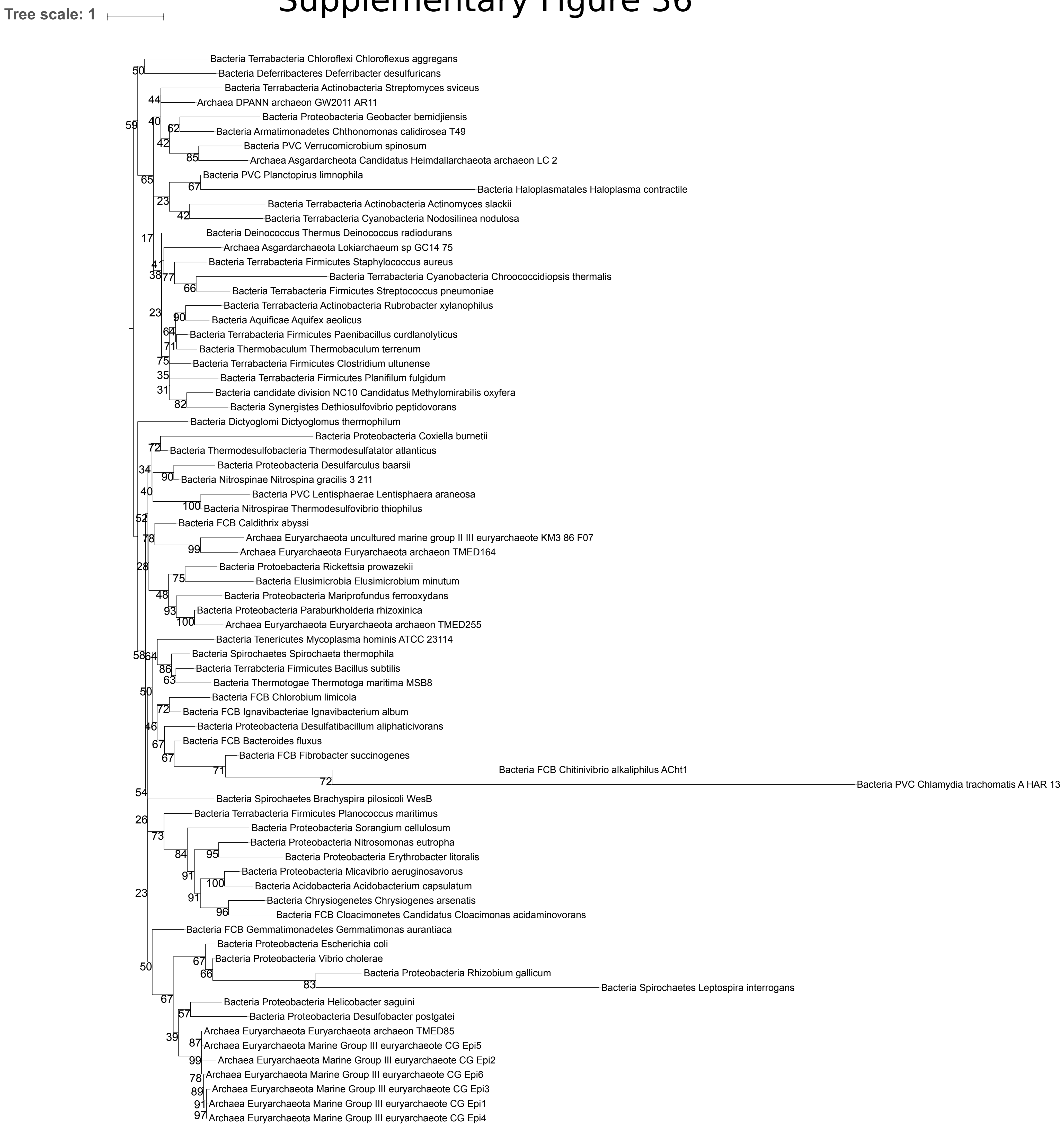

Supplementary Figure 37

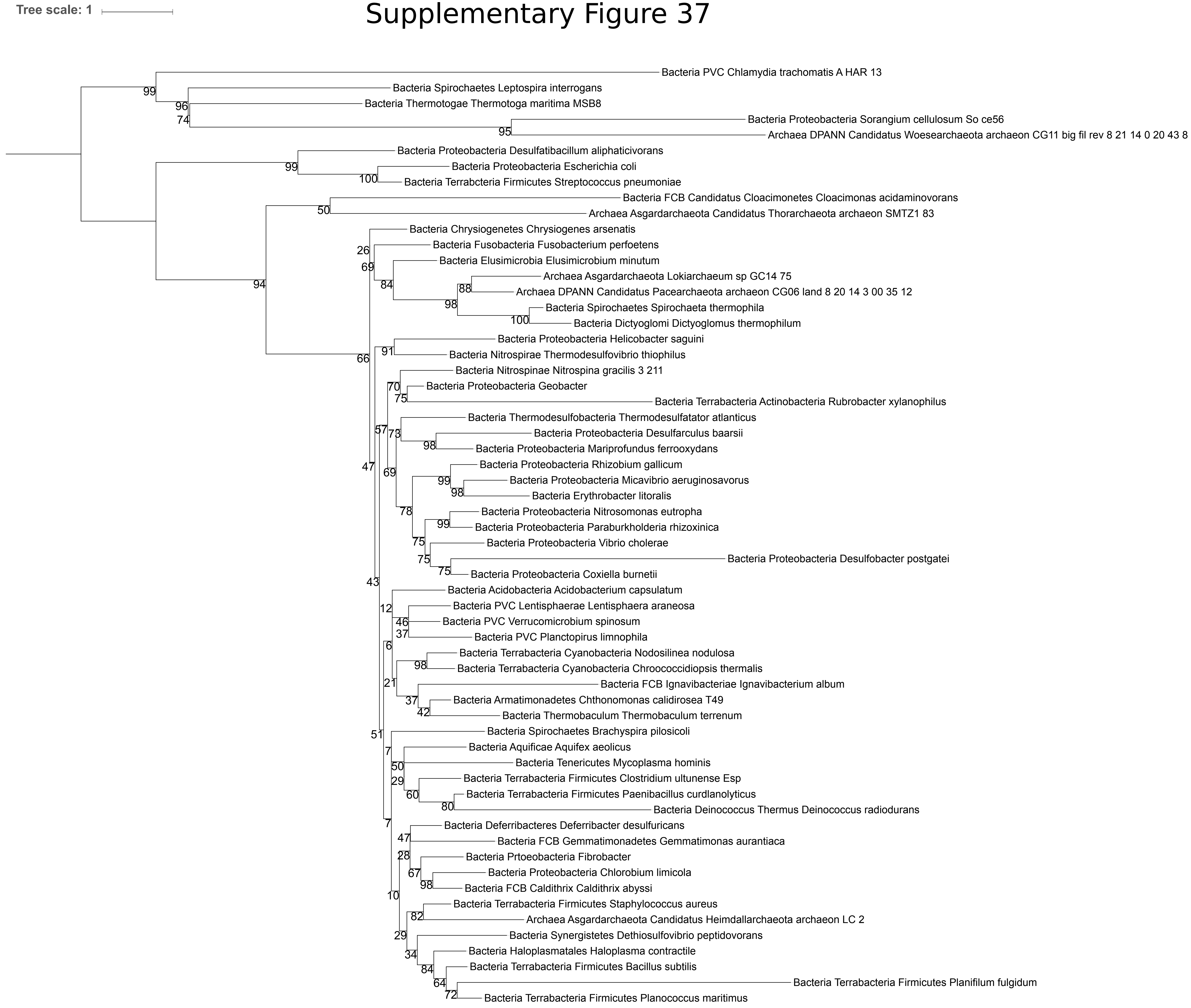

Supplementary Figure 38

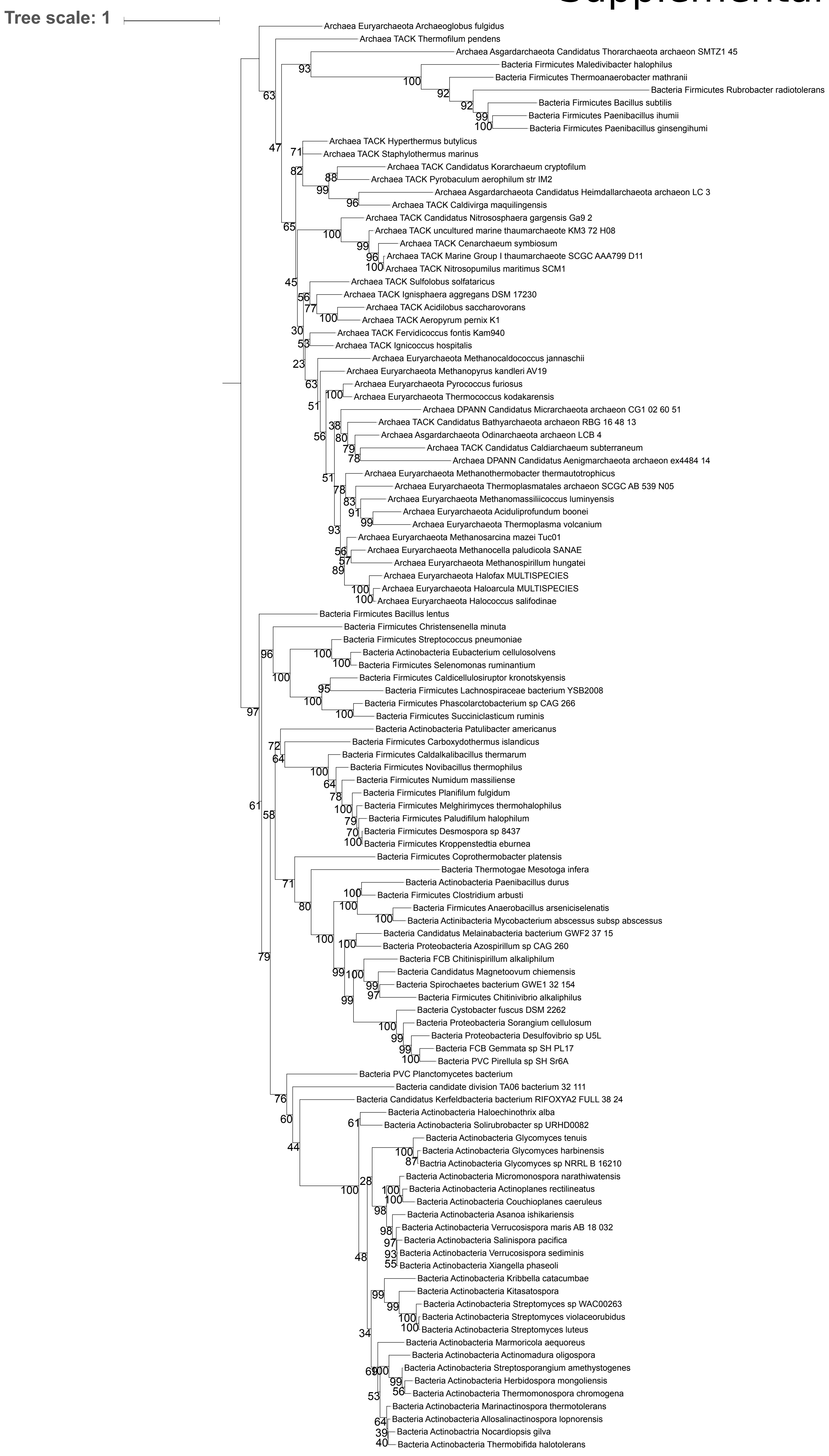

Supplementary Figure 39

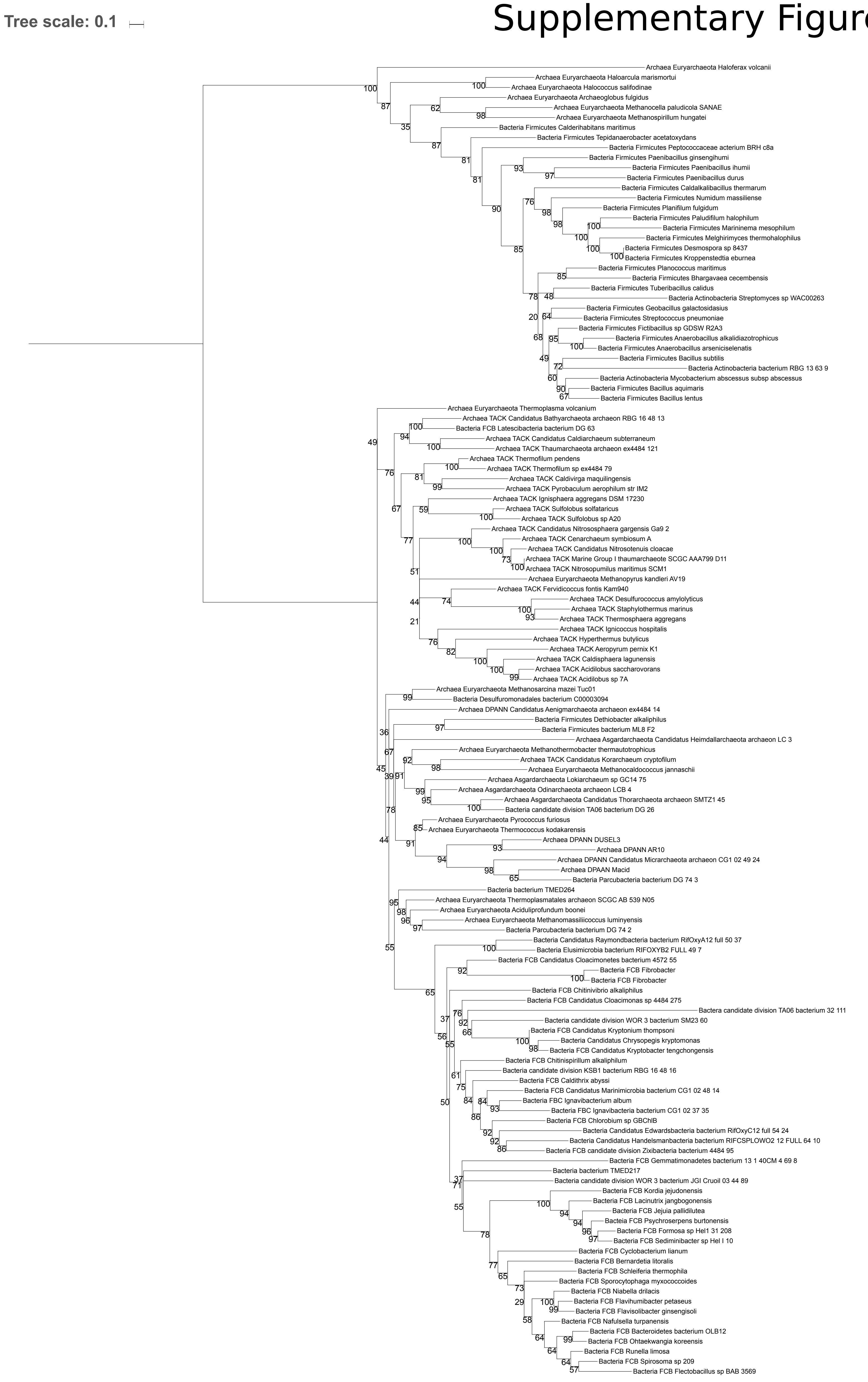

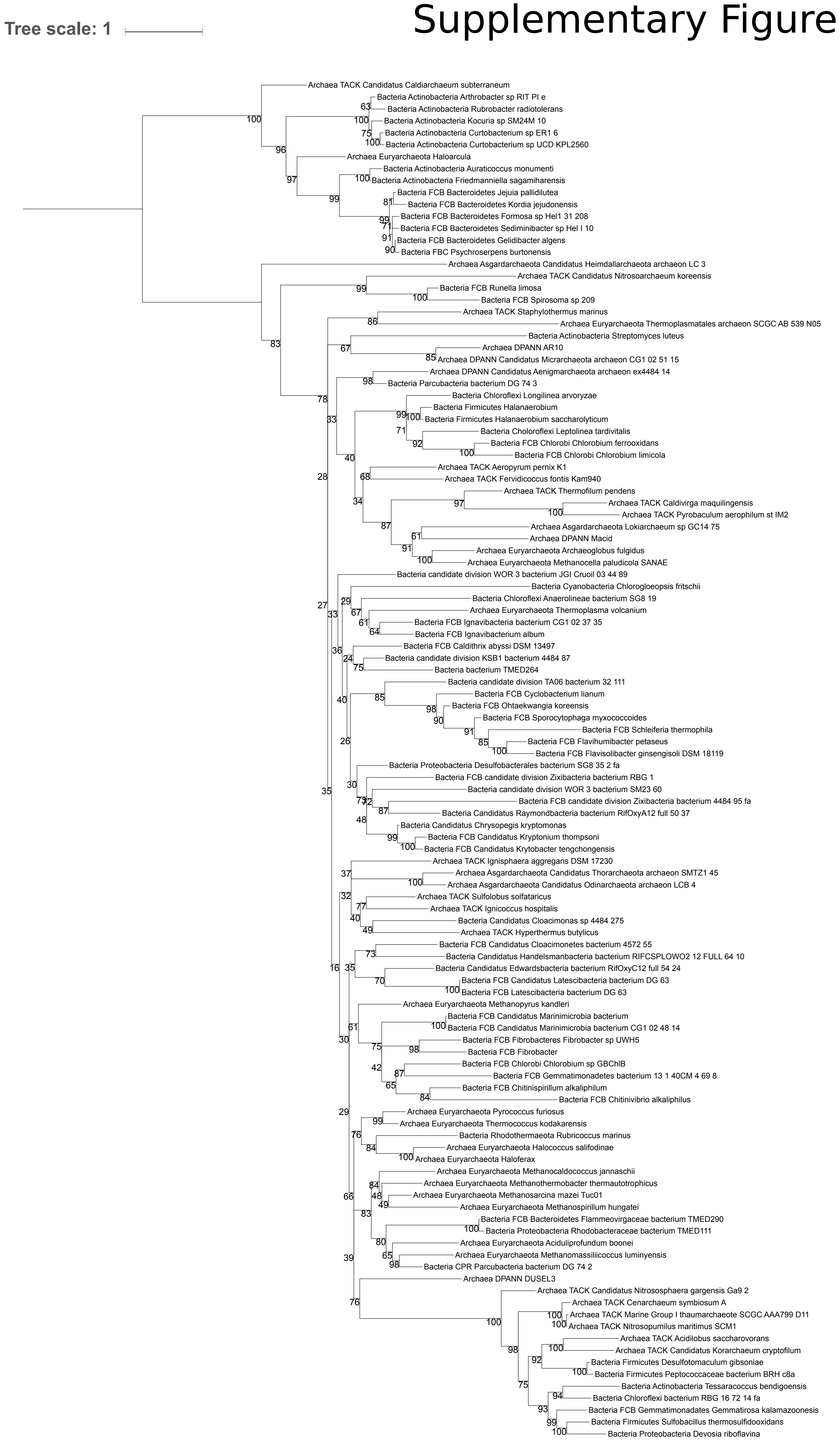

Supplementary Figure 40

Supplementary Figure 41

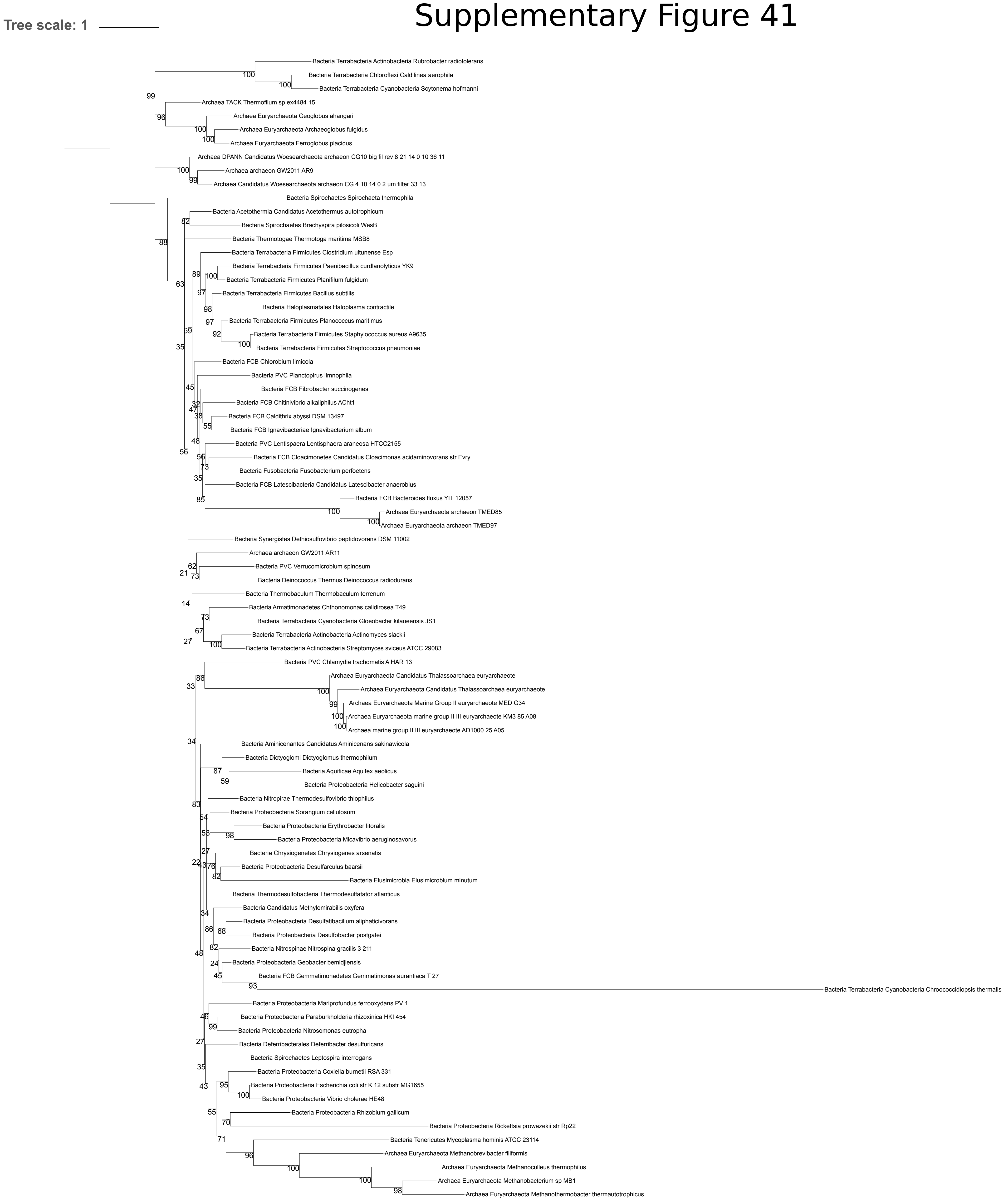

Supplementary Figure 42

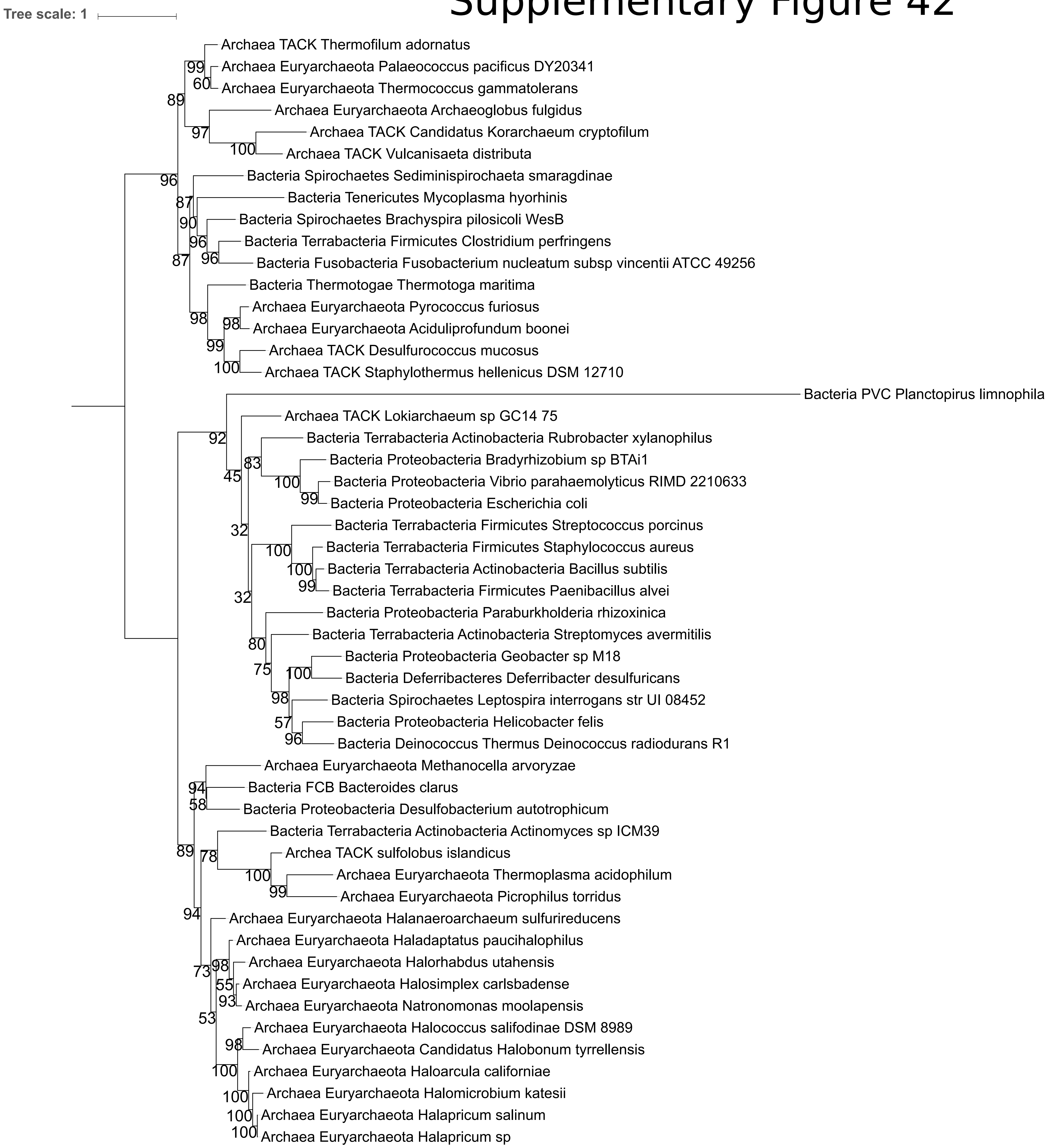

Supplementary Figure 43

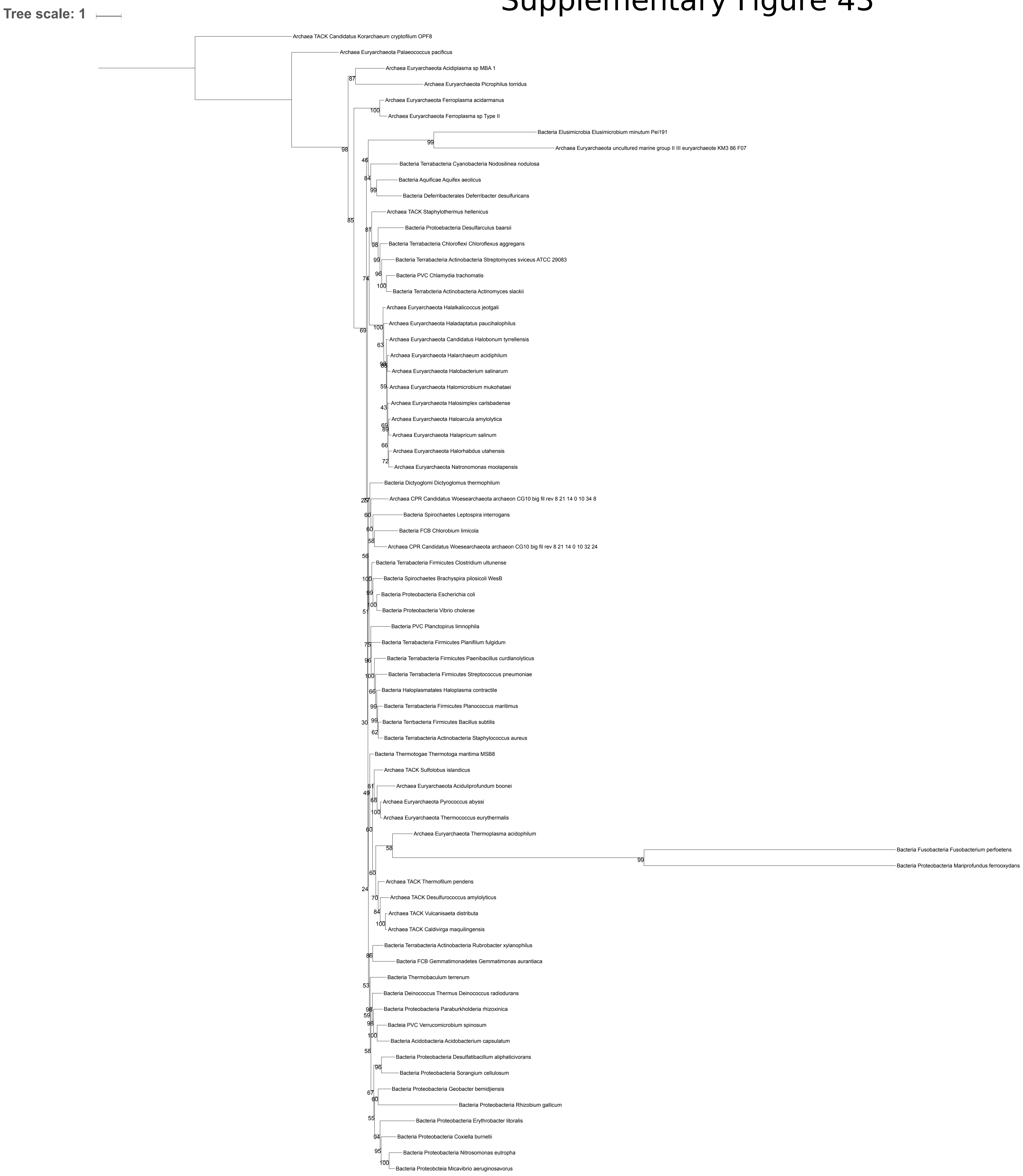

Supplementary Figure 44

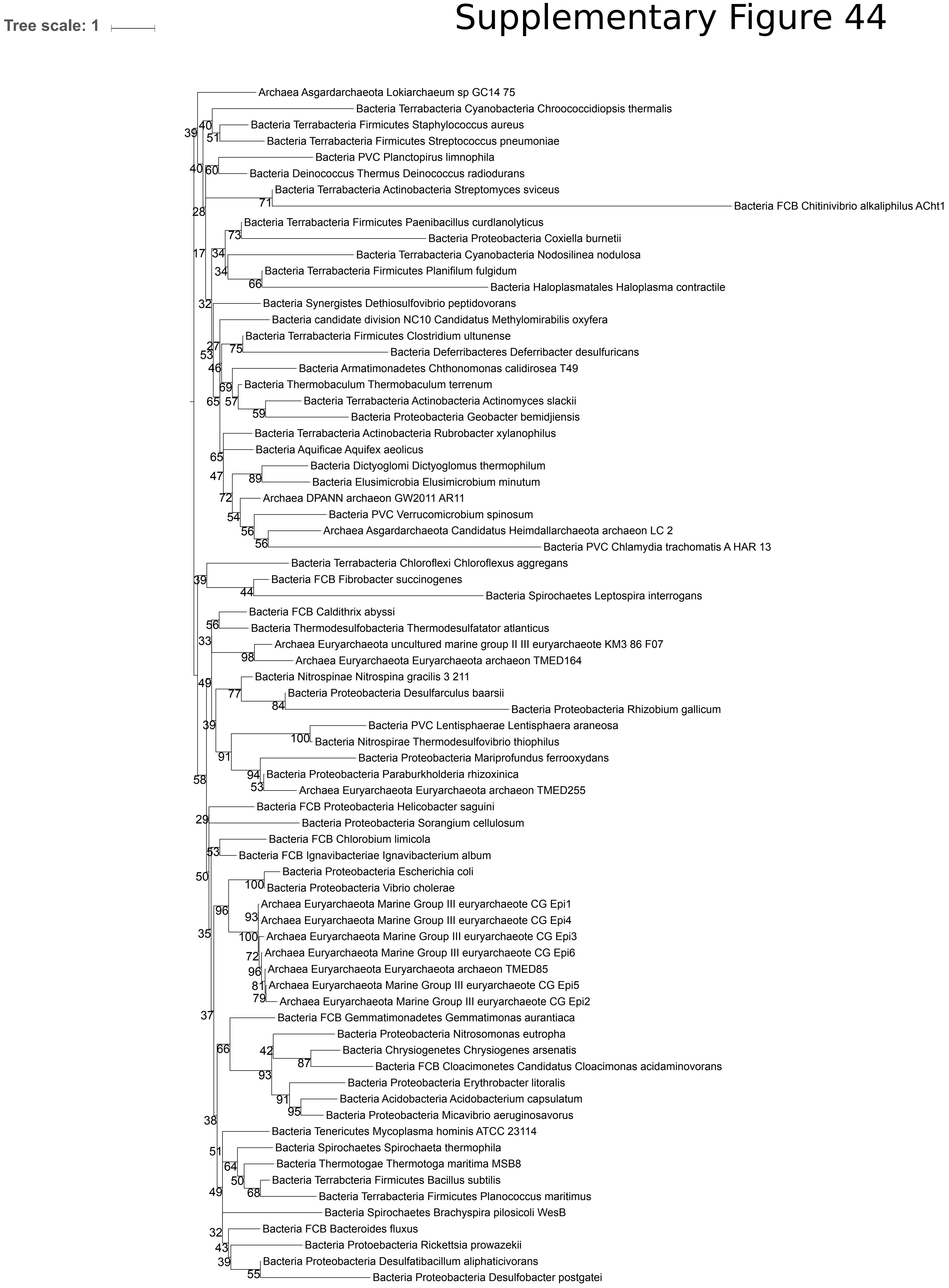

Supplementary Figure 45

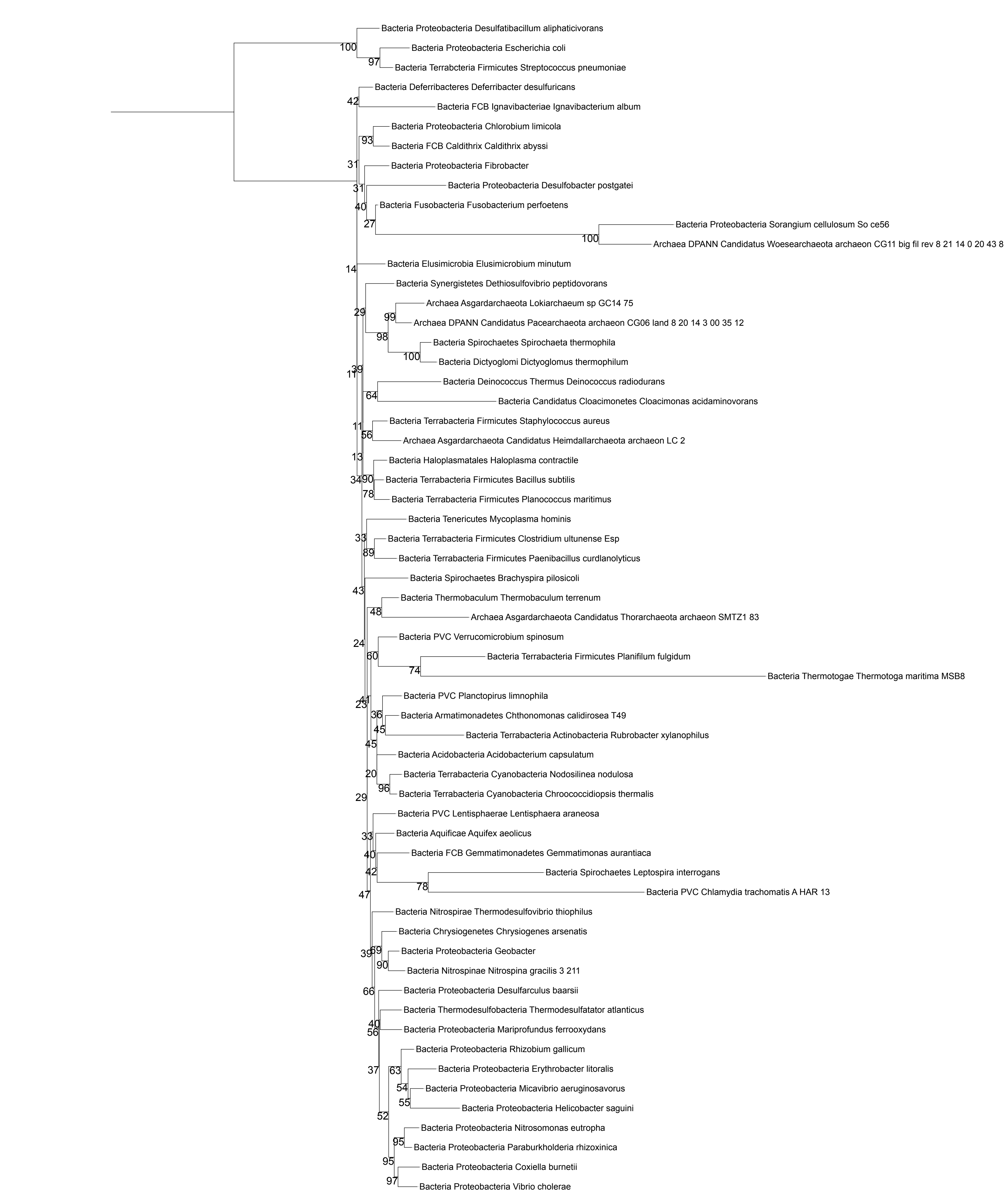

Supplementary Figure 46

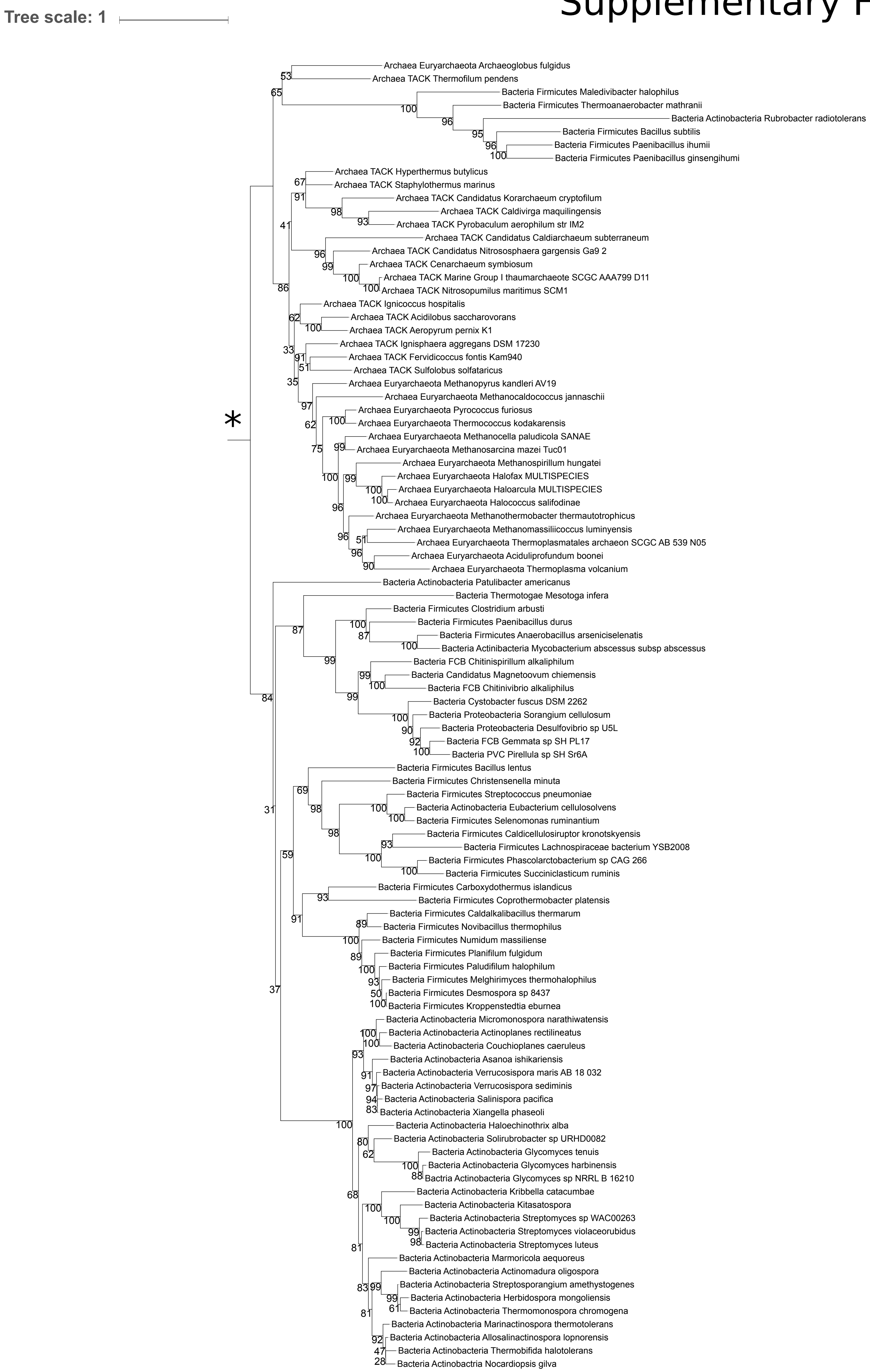

Supplementary Figure 47

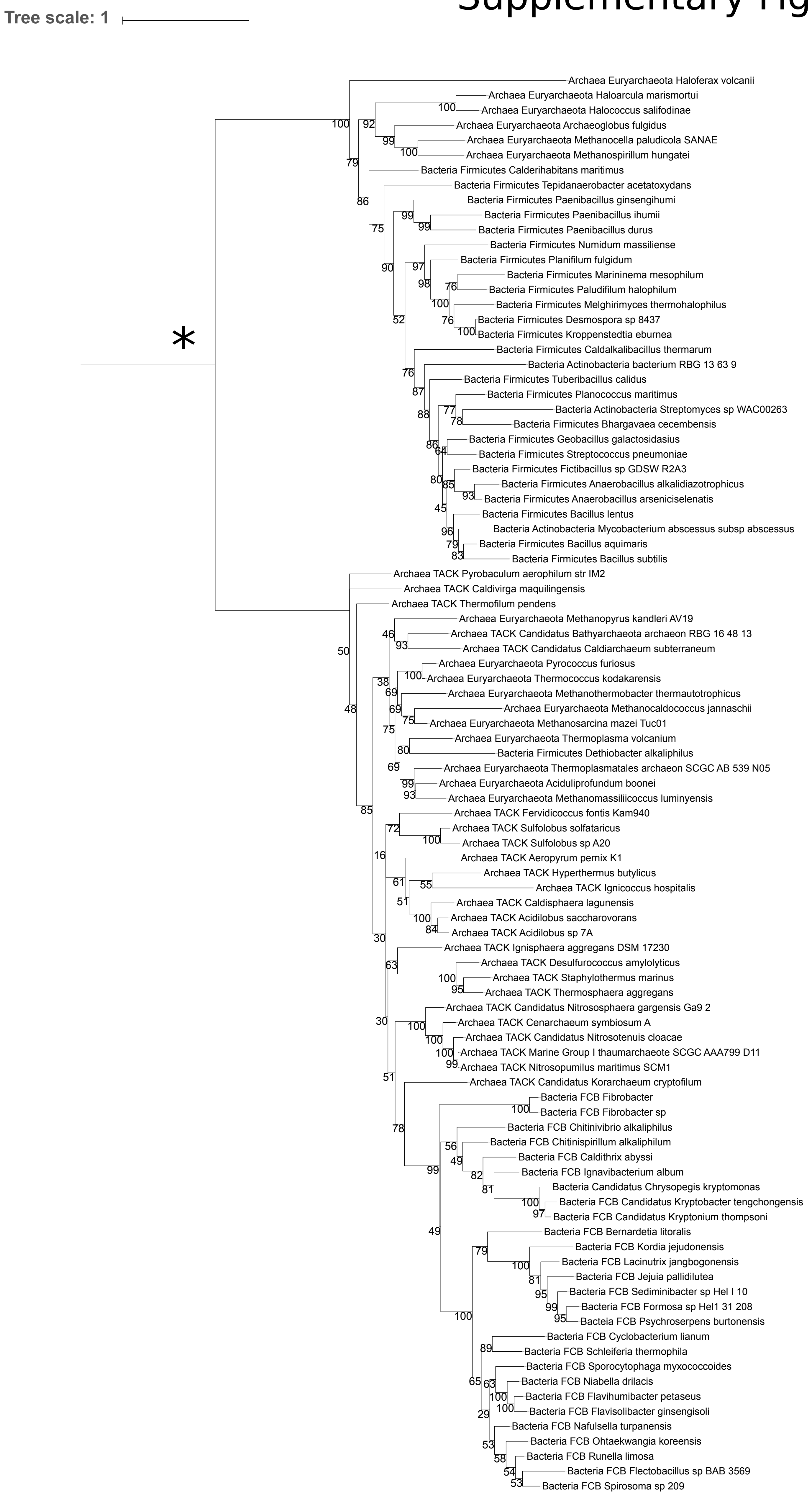

Supplementary Figure 48

Tree scale: 0.1

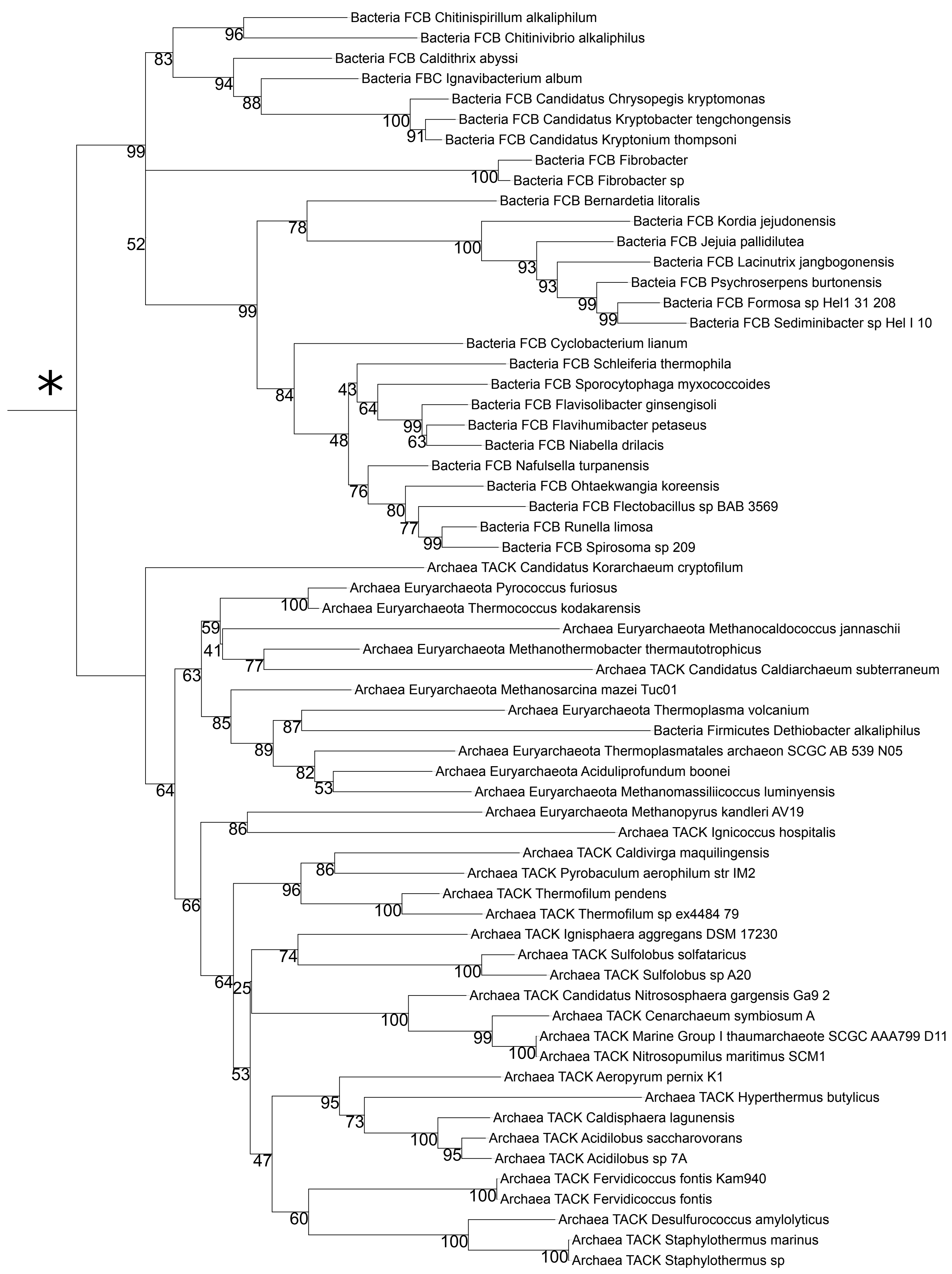

Supplementary Figure 49

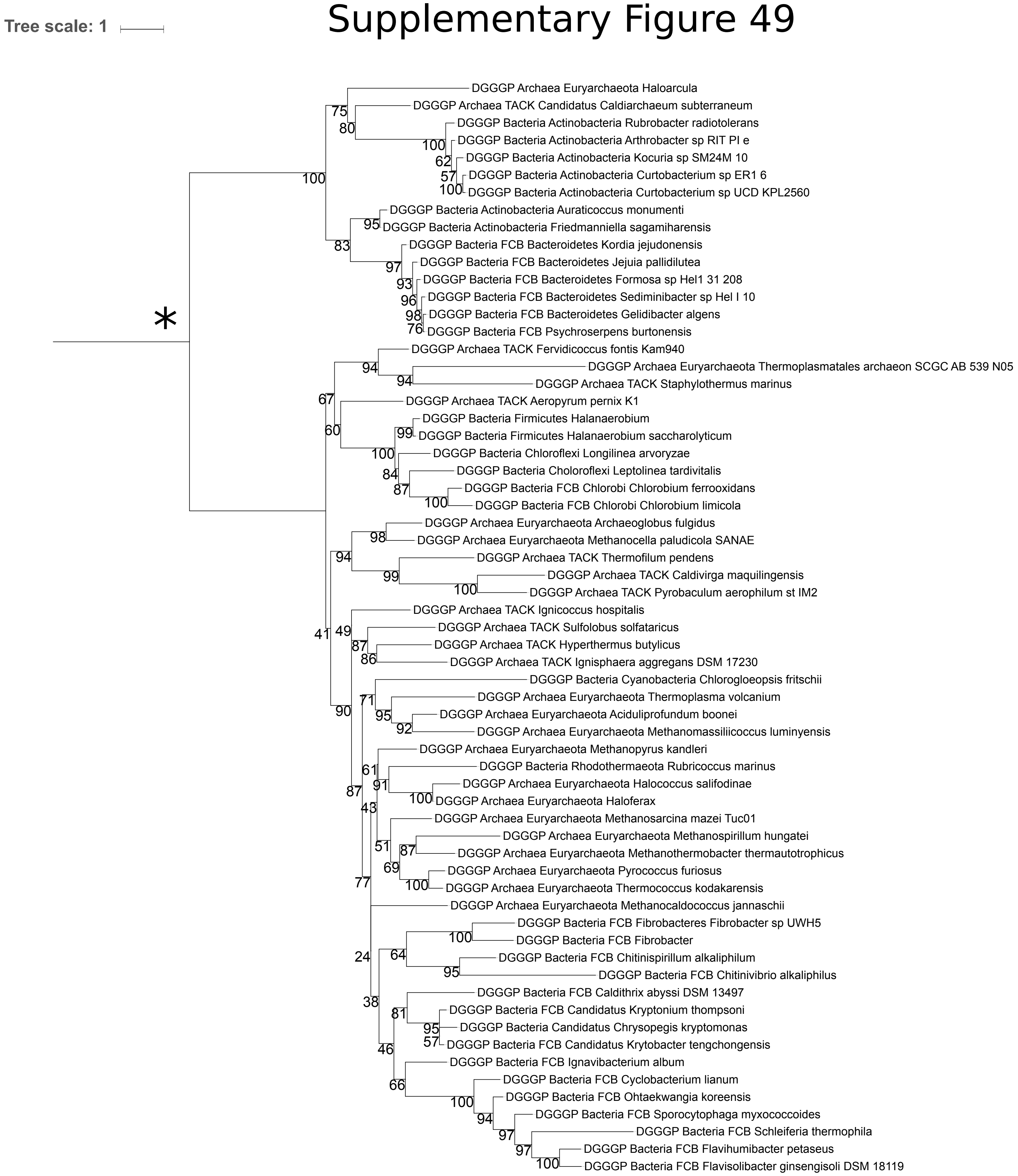

Supplementary Figure 50

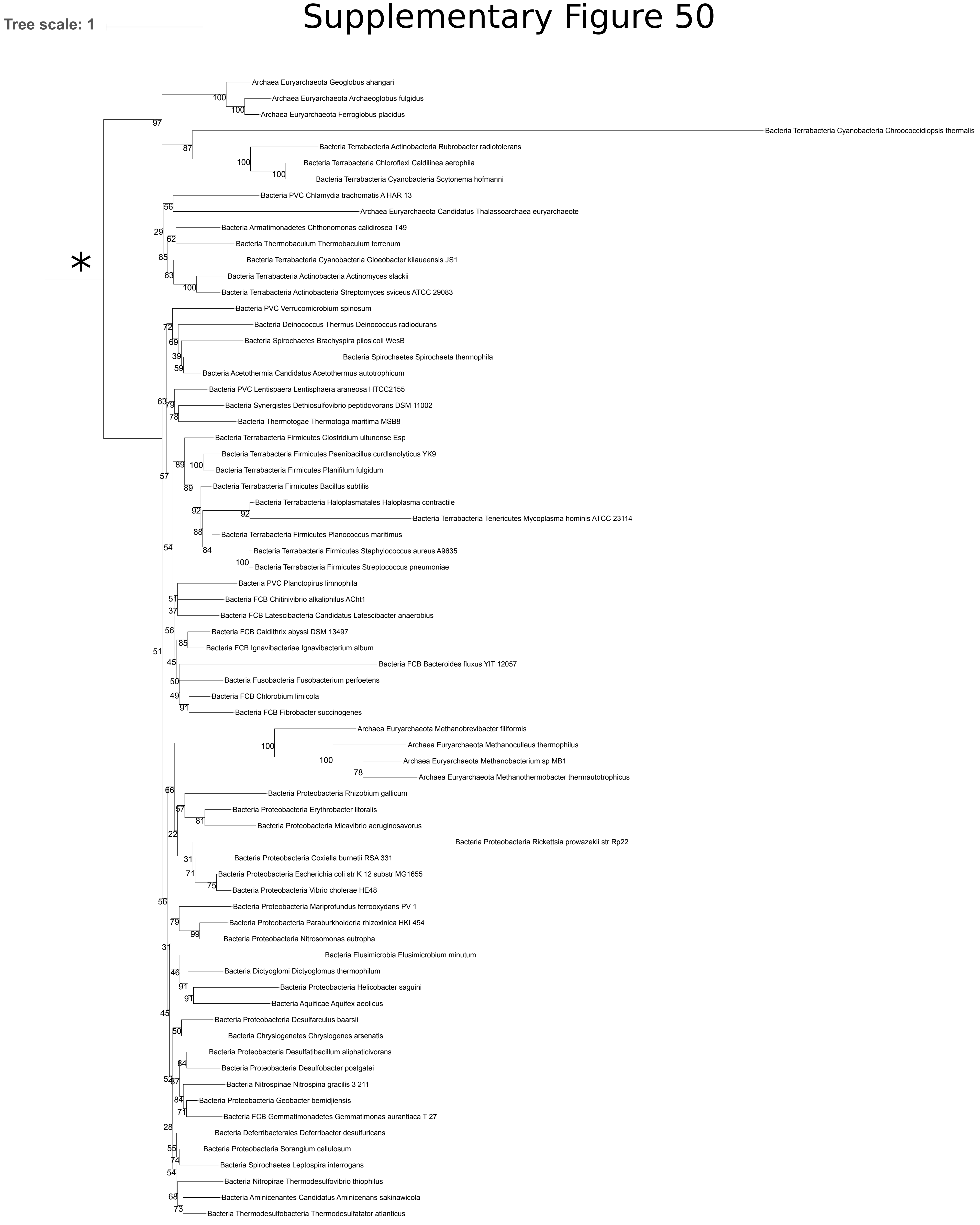

Supplementary Figure 51

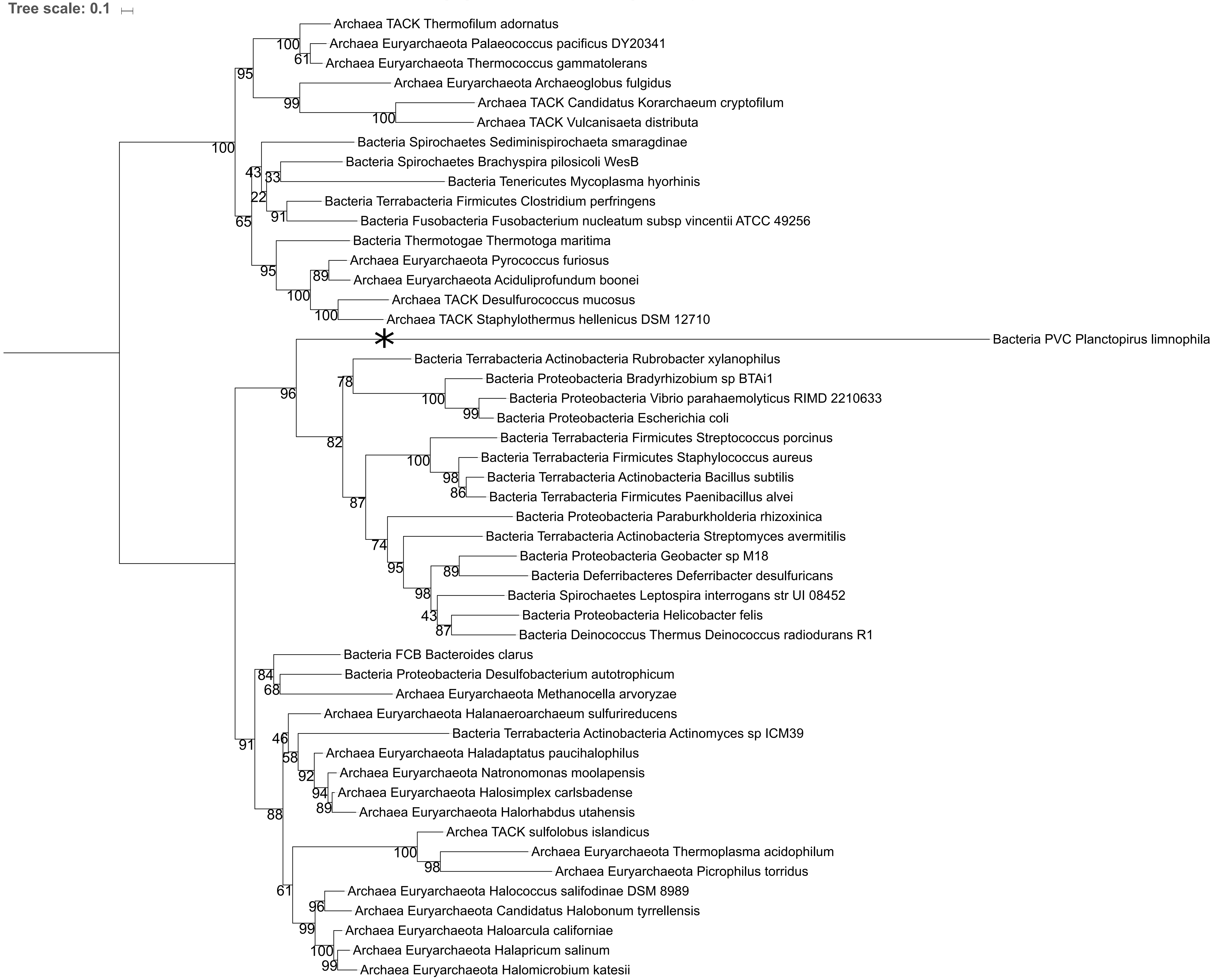

Supplementary Figure 52

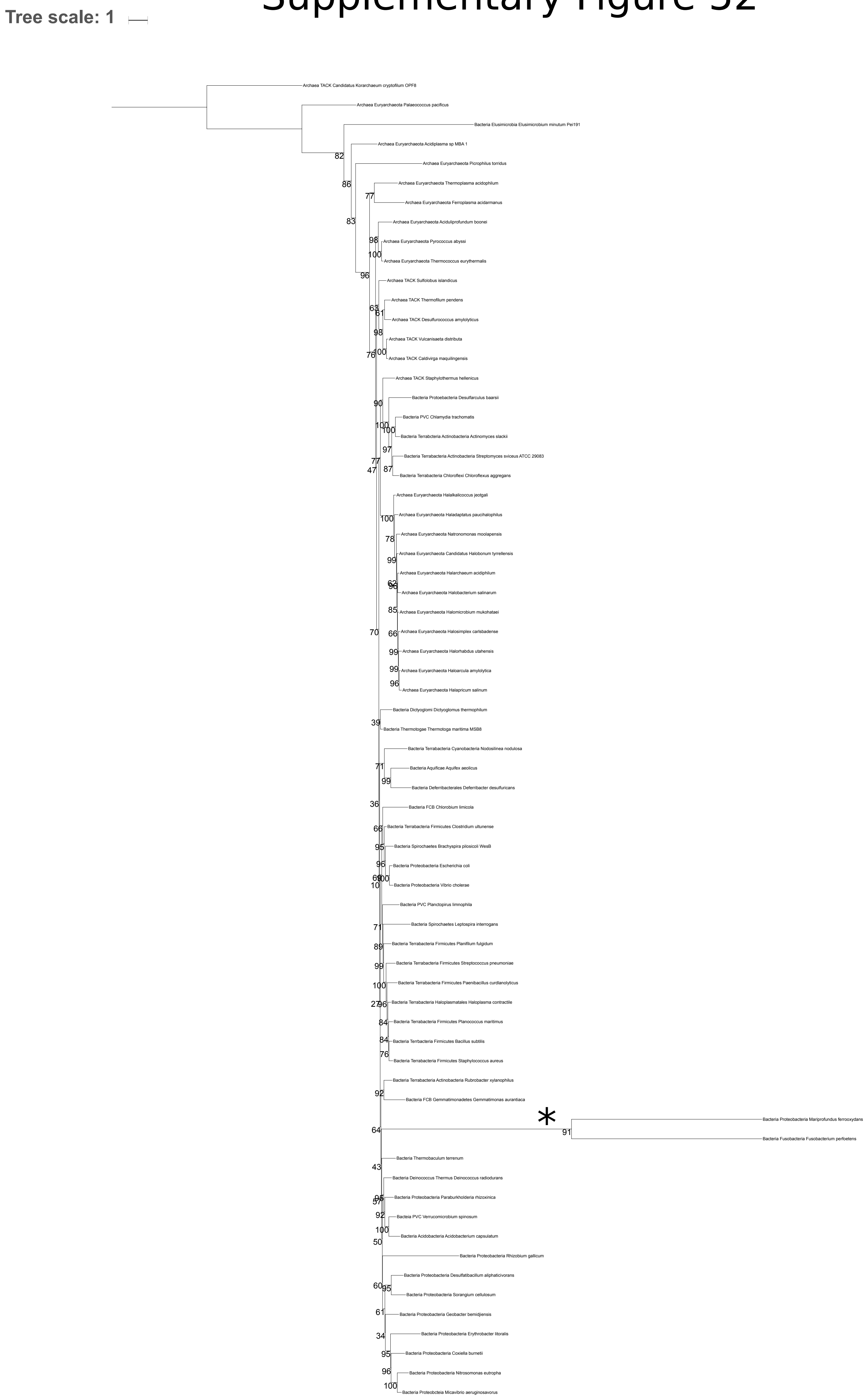

Supplement: Supplementary Data [file evz034_supp.zip › Supplementary_figures.pdf]
